# The Burden of Primary Liver Cancer and Underlying Etiologies From 1990 to 2015 at the Global, Regional, and National Level: Results From the Global Burden of Disease Study 2015

**DOI:** 10.1001/jamaoncol.2017.3055

**Published:** 2017-10-05

**Authors:** Global Burden of Disease Liver Cancer Collaboration, Tomi Akinyemiju, Semaw Abera, Muktar Ahmed, Noore Alam, Mulubirhan Assefa Alemayohu, Christine Allen, Rajaa Al-Raddadi, Nelson Alvis-Guzman, Yaw Amoako, Al Artaman, Tadesse Awoke Ayele, Aleksandra Barac, Isabela Bensenor, Adugnaw Berhane, Zulfiqar Bhutta, Jacqueline Castillo-Rivas, Abdulaal Chitheer, Jee-Young Choi, Benjamin Cowie, Lalit Dandona, Rakhi Dandona, Subhojit Dey, Daniel Dicker, Huyen Phuc, Donatus U. Ekwueme, Maysaa El Sayed Zaki, Florian Fischer, Thomas Fürst, Jamie Hancock, Simon I. Hay, Peter Hotez, Sun Ha Jee, Amir Kasaeian, Yousef Khader, Young-Ho Khang, G Anil Kumar, Michael Kutz, Heidi Larson, Alan Lopez, Raimundas Lunevicius, Reza Malekzadeh, Colm McAlinden, Toni Meier, Walter Mendoza, Ali Mokdad, Maziar Moradi-Lakeh, Gabriele Nagel, Quyen Nguyen, Grant Nguyen, Felix Ogbo, George Patton, David M. Pereira, Farshad Pourmalek, Mostafa Qorbani, Amir Radfar, Gholamreza Roshandel, Joshua A Salomon, Juan Sanabria, Benn Sartorius, Maheswar Satpathy, Monika Sawhney, Sadaf Sepanlou, Katya Shackelford, Hirbo Shore, Jiandong Sun, Desalegn Tadese Mengistu, Roman Topór-Mądry, Bach Tran, Kingsley Nnanna Ukwaja, Vasiliy Vlassov, Stein Emil Vollset, Theo Vos, Tolassa Wakayo, Elisabete Weiderpass, Andrea Werdecker, Naohiro Yonemoto, Mustafa Younis, Chuanhua Yu, Zoubida Zaidi, Liguo Zhu, Christopher J. L. Murray, Mohsen Naghavi, Christina Fitzmaurice

**Affiliations:** 1 School of Public Health, Birmingham, University of Alabama at Birmingham; 2 Mekelle University, School of Public Health, College of Health Sciences, Mekelle, Tigray, Ethiopia; 3 University of Hohenheim, Institute of Biological Chemistry and Nutrition, Stuttgart, Baden Württemberg, Germany; 4 Jimma University Institute of Health, Department of Epidemiology, Jimma, Oromiya, Ethiopia; 5 Department of Health, Queensland Government, Herston, QLD, Australia; 6 University of Queensland, School of Public Health, Herston, QLD, Australia; 7 Mekelle University Epidemiology, Mekelle, TNRS, Ethiopia; 8 University of Washington, Institute for Health Metrics and Evaluation, Seattle; 9 Ministry of Health Research Department, Jeddah, Saudi Arabia; 10 Universidad de Cartagena, Grupo de Investigación en Economía de la Salud, Cartagena, Bolivar, Colombia; 11 Komfo Anokye Teaching Hospital, Department of Medicine, Bantama, Ghana; 12 University of Manitoba, Community Health Sciences, Winnipeg, Manitoba, Canada; 13 University of Gondar, Epidemiology and Biostatistics, Gondar, Ethiopia; 14 Clinical Center of Serbia, Clinic for Infectious and Tropic Diseases, Belgrade, Serbia; 15 Hospital Universitário, University of São Paulo Division of Internal Medicine, São Paulo, São Paulo, Brazil; 16 Debre Berhan University, College of Health Sciences, Debre Berhan, Amhara, Ethiopia; 17 Aga Khan University, Centre of Excellence in Women & Child, Karachi, Sindh, Pakistan; 18 The Hospital for Sick Children, Centre for Global Child Health, Toronto, Ontario, Canada; 19 Caja Costarricense de Seguro Social, Dirección Actuarial y Economica, San Jose, San Jose, Costa Rica; 20 Iraq MOH FETP, MOH, Baghdad, Iraq; 21 Seoul National University, College of Medicine Medical Library, Seoul, South Korea; 22 Doherty Institute, WHO Collaborating Centre for Viral Hepatitis, Melbourne, Victoria, Australia; 23 Public Health Foundation of India, Research, Gurgaon, NCR, India; 24 Indian Institute of Public Health-Delhi, Environmental and Occupational Health, Gurgaon, Haryana, India; 25 Duy Tan University, Institute for Global Health Innovations, Da Nang, Vietnam; 26 Division of Cancer Prevention and Control, Centers for Disease Control and Prevention, Atlanta, Georgia; 27 Clinical Pathology Department, Mansoura Faculty of Medicine, Mansoura, Egypt; 28 Bielefeld University, School of Public Health, Bielefeld, Germany; 29 Swiss Tropical and Public Health Institute, Epidemiology and Public Health, Basel, Switzerland; 30 University of Basel, Switzerland; 31 Imperial College London, School of Public Health, London, England; 32 Baylor College of Medicine, National School of Tropical Medicine, Houston, Texas; 33 Sabin Vaccine Institute & Texas Children's Hospital Center for Vaccine Development, Houston; 34 Graduate School of Public Health, Yonsei University, Epidemiology and Health Promotion, Seoul, South Korea; 35 Tehran University of Medical Sciences, Hematology-Oncology and Stem Cell Transplantation Research Center, Tehran, Tehran, Iran; 36 Jordan University of Science and Technology, Public Health, Irbid, Irbid, Jordan; 37 Seoul National University College of Medicine, Institute of Health Policy and Management, Seoul, Seoul Metropolitan City, South Korea; 38 Public Health Foundation of India Research, Gurgaon (NCR), Haryana, India; 39 Department Infectious Disease Epidemiology, London School of Hygiene & Tropical Medicine, London, England; 40 University of Melbourne, Melbourne School of Population and Global Health, Melbourne, VIC, Australia; 41 Aintree University Hospital NHS Foundation Trust, General Surgery Department, Liverpool, England; 42 School of Medicine, University of Liverpool, Liverpool, England; 43 Tehran University of Medical Sciences, Digestive Diseases Research Institute, Tehran, Tehran, Iran; 44 University Hospitals Bristol, Department of Medicine, Bristol, England; 45 Martin Luther University Halle-Wittenberg, Institute for Agricultural and Nutritional Sciences, Halle (Saale), Germany; 46 UNFPA Peru Country Office, Lima, Peru; 47 Iran University of Medical Sciences, Gastrointestinal and Liver Disease Research Center, Tehran, Tehran, Iran; 48 Iran University of Medical Sciences, Preventive Medicine and Public Health Research Center, Tehran, Tehran, Iran; 49 Ulm University, Institute of Epidemiology and Medical Biometry, Ulm, Germany; 50 Duy Tan University, Institute for Global Health Innovations, Da Nang, Vietnam; 51 Western Sydney University, Centre for Health Research, School of Medicine, Penrith, NSW, Australia; 52 Ingham Institute for Applied Medical Research, Liverpool, NSW, Australia; 53 University of Melbourne, Paediatrics, Melbourne, Victoria, Australia; 54 REQUIMTE/LAQV, Laboratório de Farmacognosia, Departamento de Química, Faculdade de Farmácia, Universidade do Porto, Porto, Portugal; 55 Department of Urology, University of British Columbia, Vancouver, British Columbia, Canada; 56 Alborz University of Medical Sciences, Noncommunicable Diseases Research Center, Karaj, Alborz, Iran; 57 A. T. Still University, College of Graduate Health Studies, Mesa, Arizona; 58 Golestan University of Medical Sciences, Golestan Research Center of Gastroenterology and Hepatology, Gorgan, Iran; 59 Harvard T.H. Chan School of Public Health, Department of Global Health and Population, Boston, Massachusetts; 60 Marshall University School of Medicine, Surgery, Huntington, West Virginia; 61 Case Western Reserve University, Nutrition and Preventive Medicine, Ohio; 62 University of KwaZulu-Natal, Public Health Medicine, Durban, KwaZulu-Natal, South Africa; 63 Utkal University, Centre for Advanced Study in Psychology, Bhubaneswar, Odisha, India; 64 AIIMS New Delhi, JPN Apex Trauma Centre, New Delhi, Delhi, India; 65 Marshall University Public Health, Huntington, West Virginia; 66 Tehran University of Medical Sciences, Digestive Diseases Research Institute, Tehran, Tehran, Iran; 67 Haramaya University School of Public Health, Harari, Harari, Ethiopia; 68 Queensland University of Technology, School of Public Health and Social Work, Brisbane, Queensland, Australia; 69 Mekelle University, Institute of Biomedical Sciences, Mekelle, Tigrai, Ethiopia; 70 Faculty of Health Sciences Jagiellonian University Medical College, Institute of Public Health, Kraków, Poland; 71 Faculty of Health Sciences Wroclaw Medical University, Wroclaw, Poland; 72 Hanoi Medical University, Institute for Preventive Medicine and Public Health, Hanoi, Vietnam; 73 Johns Hopkins University, Bloomberg School of Public Health, Baltimore, Maryland; 74 Federal Teaching Hospital, Department of Medicine, Abakaliki, Ebonyi State, Nigeria; 75 Department of Health Care Administration and Economy, National Research University Higher School of Economics, Moscow, Russia; 76 Norwegian Institute of Public Health, Centre for Disease Burden, Bergen, Norway; 77 University of Bergen, Department of Global Public Health and Primary Care, Bergen, Norway; 78 Jimma University, Population and Family Health, Oromia, Ethiopia; 79 Cancer Registry of Norway, Institute of Population Based Cancer Research, Oslo, Norway; 80 University of Tromsø, The Arctic University of Norway, Department of Community Medicine, Faculty of Health Sciences, Tromsø, Norway; 81 Department of Medical Epidemiology and Biostatistics, Karolinska Institutet, Stockholm, Sweden; 82 Federal Institute for Population Research, Competence Center Mortality-Follow-Up of the National Cohort, Wiesbaden, Hesse, Germany; 83 Kyoto University, School of Public Health Biostatistics, Sakyo, Kyoto, Japan; 84 Jackson State University, Health Policy & Management, Jackson, Mississippi; 85 Harvard Asia Aging Center, Harvard Medical School, Boston, Massachuetts; 86 Department of Epidemiology and Biostatistics, Wuhan University, Wuhan, Hubei Province, China; 87 Department of Epidemiology, University Hospital of Setif, Setif, Algeria; 88 University Ferhat Abbas, Faculty of Medicine, Setif, Algeria; 89 Jiangsu Provincial Center for Disease Control and Prevention, Major Project Execution Office, Nanjing, Jiangsu, China; 90 Division of Hematology, Department of Medicine, University of Washington, Seattle

## Abstract

**Importance:**

Liver cancer is among the leading causes of cancer deaths globally. The most common causes for liver cancer include hepatitis B virus (HBV) and hepatitis C virus (HCV) infection and alcohol use.

**Objective:**

To report results of the Global Burden of Disease (GBD) 2015 study on primary liver cancer incidence, mortality, and disability-adjusted life-years (DALYs) for 195 countries or territories from 1990 to 2015, and present global, regional, and national estimates on the burden of liver cancer attributable to HBV, HCV, alcohol, and an “other” group that encompasses residual causes.

**Design, Settings, and Participants:**

Mortality was estimated using vital registration and cancer registry data in an ensemble modeling approach. Single-cause mortality estimates were adjusted for all-cause mortality. Incidence was derived from mortality estimates and the mortality-to-incidence ratio. Through a systematic literature review, data on the proportions of liver cancer due to HBV, HCV, alcohol, and other causes were identified. Years of life lost were calculated by multiplying each death by a standard life expectancy. Prevalence was estimated using mortality-to-incidence ratio as surrogate for survival. Total prevalence was divided into 4 sequelae that were multiplied by disability weights to derive years lived with disability (YLDs). DALYs were the sum of years of life lost and YLDs.

**Main Outcomes and Measures:**

Liver cancer mortality, incidence, YLDs, years of life lost, DALYs by etiology, age, sex, country, and year.

**Results:**

There were 854 000 incident cases of liver cancer and 810 000 deaths globally in 2015, contributing to 20 578 000 DALYs. Cases of incident liver cancer increased by 75% between 1990 and 2015, of which 47% can be explained by changing population age structures, 35% by population growth, and −8% to changing age-specific incidence rates. The male-to-female ratio for age-standardized liver cancer mortality was 2.8. Globally, HBV accounted for 265 000 liver cancer deaths (33%), alcohol for 245 000 (30%), HCV for 167 000 (21%), and other causes for 133 000 (16%) deaths, with substantial variation between countries in the underlying etiologies.

**Conclusions and Relevance:**

Liver cancer is among the leading causes of cancer deaths in many countries. Causes of liver cancer differ widely among populations. Our results show that most cases of liver cancer can be prevented through vaccination, antiviral treatment, safe blood transfusion and injection practices, as well as interventions to reduce excessive alcohol use. In line with the Sustainable Development Goals, the identification and elimination of risk factors for liver cancer will be required to achieve a sustained reduction in liver cancer burden. The GBD study can be used to guide these prevention efforts.

## Key Points

**Question:**

What is the burden of liver cancer globally, what are the major risk factors for liver cancer across countries, regions, and at the global level and how did these change between 1990 and 2015?

**Findings:**

There were 854 000 incident liver cancer cases and 810 000 deaths globally in 2015, contributing to 20 578 000 disability-adjusted life-years. Hepatitis B virus infection accounted for 265 000 liver cancer deaths (33%), alcohol for 245 000 (30%), hepatitis C virus infection for 167 000 (21%), and other causes for 133 000 (16%) deaths.

**Meaning:**

Most cases of liver cancer can be prevented through vaccination, antiviral treatment, safe blood transfusion and injection practices, as well as interventions to reduce excessive alcohol use.

## Teaser

This data analysis of the Global Burden of Disease 2015 study on primary liver cancer reports incidence, mortality, and disability-adjusted life-years for 195 countries or territories from 1990 to 2015, and presents global, regional, and national estimates on the burden of liver cancer.

## Introduction

Liver cancer was the fourth leading cause of cancer death in 2015 after lung, colorectal, and stomach cancer.^1^ The most common type of primary liver cancer globally is hepatocellular carcinoma, followed by cholangiocarcinoma.^2^ Liver cancer burden varies markedly by sex and geographic region due to risk factor exposure.^3^ Major risk factors include infections (hepatitis B virus [HBV], hepatitis C virus [HCV], liver flukes in endemic areas), behavioral factors (alcohol, tobacco), metabolic factors (excess body fatness), and aflatoxins.^4^

As part of the Sustainable Development Goals and World Health Organization strategies for noncommunicable diseases and viral hepatitis, primary prevention targets include eliminating viral hepatitis as a major public health threat by 2030, reducing the harmful use of alcohol and tobacco, and controlling diabetes and obesity.^6^ Because of the lag between risk factor exposure and development of liver cancer, even best-case scenarios of these prevention approaches are unlikely to reduce the number of patients with liver cancer that health care systems have to accommodate in the foreseeable future. The analysis of liver cancer as part of the Global Burden of Disease (GBD) 2015 study therefore serves 2 main purposes: first, to provide detailed information on liver cancer etiologies and their trends over time, without which targeted prevention strategies are impossible to design and to evaluate; and second, to promote strategic investments into research and clinical resources.

Prior studies analyzing liver cancer burden have either focused on single countries or regions, single years, or a subset of the most common etiologies like HBV and HCV.^8^ To our knowledge, no prior study has provided estimates for all countries, over time, covering the main risk factors for liver cancer. In this study we report results of the GBD 2015 study on primary liver cancer incidence, mortality, and disability-adjusted life-years (DALYs) for 195 countries or territories from 1990 to 2015 by sex, as well as on the burden of liver cancer attributable to HBV, HCV, alcohol, and a remaining “other” group that encompasses residual causes.

## Methods

General methods for the GBD 2015 study have been published previously.^1^ Herein, we present methods pertaining to the liver cancer estimation. Descriptions of the estimation process are available in the eAppendix in Supplement 1 (eFigure 1, eFigure 2, and eTable 1).

The estimation process starts with liver cancer mortality, which we estimated using vital registration system data and cancer registry incidence data that were transformed to mortality estimates using separately modeled mortality-to-incidence ratios.^16^ Data were processed to adjust for aggregated causes, age groups, or uninformative causes of death.^1^ Liver cancer mortality was modeled by developing a large set of plausible models using different model types and combinations of covariates, that were tested using out-of-sample predictive validity (eTable 3 and eTable 4 in Supplement 1).^17^ The 2.5% and 97.5% quantiles from 1000 draws of the posterior distribution were used to generate 95% uncertainty intervals (UI). Liver cancer mortality was scaled with other causes of death to sum to 100% of the demographic estimates of all-cause mortality.^1^ Years of life lost were calculated by multiplying each death by the standard life expectancy.^1^ To generate mortality estimates for 4 liver cancer etiologies, proportions of liver cancer due to different causes were identified in a systematic review (eTable 5 in Supplement 1). Cases were attributed to HBV, HCV, alcohol, and other causes, which include remaining etiologies like liver flukes, nonalcoholic steatohepatitis, and aflatoxins. To estimate proportions for all locations, by sex, and over time, models were generated using DisMod-MR 2.1, a Bayesian meta-regression model (eAppendix in Supplement 1).^18^ Liver cancer mortality estimates were split into etiologies using the modeled proportions. Liver cancer incidence was estimated by dividing mortality by mortality-to-incidence ratios. Survival was estimated based on a theoretical best and worst liver cancer survival and a scaling factor derived from age-standardized mortality-to-incidence ratios.^16^ Prevalence was calculated using incidence and survival estimates and divided into 4 phases reflecting changing disability during: (1) diagnosis and treatment; (2) remission; (3) disseminated; and (4) terminal phase. Prevalence for each phase was multiplied by distinct disability weights to generate years lived with disability (eTable 6 in Supplement 1).^19^ The sum of years of life lost and years lived with disability represents DALYs. One DALY can be interpreted as 1 lost year of “healthy life.”

To group countries with similar development status, a Sociodemographic Index (SDI) was used, which combines total fertility rate, average educational attainment in the population over age 15, and measures of income per capita (eFigure 3 and eTable 7 in the Supplement 1).^1^

To assess the contribution of demographic vs epidemiological changes, we decomposed trends into 3 components—population aging, growth, and change in age-specific rates.^1^ Rates are reported as mean per 100 000 person-years with 95% UI in parentheses. Age-standardized rates were computed using the GBD population standard.^1^

## Results

### Liver Cancer Burden

There were 854 000 incident liver cancer cases and 810 000 deaths globally in 2015, contributing to 20 578 000 DALYs (Table; Supplement 2). Liver cancer was the sixth most common-incident cancer worldwide and the fourth most common cause of cancer death (eFigure 4 in Supplement 1). Eighty-eight percent of incident liver cancer cases and 86% of liver cancer deaths occurred in middle-SDI, high-middle–SDI, and high-SDI countries compared with low-middle–SDI and low-SDI countries. Age-standardized incidence rates (ASIR) were the highest in middle-SDI countries, followed by low-SDI countries (Table).

**Table.** Liver Cancer Incident Cases, Age-Standardized Incidence Rate, Deaths, Age-Standardized Mortality Rate, DALYs, and Age-Standardized DALYs, by Sociodemographic Quintile, Sex, and Region, 2015

Characteristic	Incident Cases, No. × 10^3 ^(95% UI)	ASIR per 100 000, No. (95% UI)	Deaths, No. × 10^3^ (95% UI)	ASMR per 100 000, No. (95% UI)	DALYs × 10^3^ (95% UI)	Age-Standardized DALY Rate per 100 000 (95% UI)
Overall	854 (768-961)	12.8 (11.4-14.3)	810 (750-863)	12.1 (11.2-12.9)	20 578 (18 938-21 915)	292.1 (269.1-311.0)
Sociodemographic index						
Low	33 (27-40)	14.5 (11.5-17.1)	37 (30-44)	16.6 (13.2-19.7)	1183 (953-1408)	431 (347.8-513.1)
Low-middle	71 (62-85)	7.1 (6.2-8.3)	75 (66-88)	7.5 (6.7-8.7)	2099 (1822-2533)	179.9 (157.9-213.7)
Middle	283 (240-343)	15.6 (13.2-18.8)	287 (262-317)	15.8 (14.5-17.5)	7814 (7052-8674)	391.1 (354.4-433.3)
High-middle	235 (200-281)	13.7 (11.6-16.3)	250 (222-274)	14.5 (12.9-15.8)	6355 (5730-7013)	345.5 (310.9-380.6)
High	233 (214-255)	11.7 (10.8-12.7)	161 (155-167)	7.9 (7.6-8.2)	3116 (2984-3276)	166.4 (159.1-176.1)
Sex						
Men	591 (517-691)	18.6 (16.3-21.6)	577 (524-622)	18.2 (16.6-19.6)	15 413 (13 994-16 747)	448.5 (408.1-486.2)
Women	264 (227-314)	7.5 (6.4-8.9)	234 (204-255)	6.6 (5.8-7.2)	5165 (4465-5689)	143.9 (124.5-158.4)
Region						
Asia Pacific, high income	93 (80-112)	26.4 (22.9-31.1)	55 (53-58)	15 (14.3-15.7)	969 (920-1021)	307.9 (291.1-326.9)
Central Asia	5 (5-6)	8.6 (7.3-9.4)	6 (5-6)	9.4 (8.1-10.2)	154 (132-167)	219.5 (188.7-237.6)
East Asia	394 (317-499)	24.3 (19.6-30.9)	415 (376-458)	25.5 (23.2-28.1)	11 227 (10 203-12 494)	644.1 (584.3-715.0)
South Asia	43 (36-52)	3.9 (3.3-4.9)	45 (40-50)	4.1 (3.8-4.7)	1147 (1027-1280)	91.3 (82.2-102.1)
Southeast Asia	67 (55-79)	13.9 (11.3-16.3)	71 (58-80)	14.6 (12.1-16.5)	1860 (1497-2138)	334.3 (271.5-381.6)
Australasia	3 (2-4)	6.5 (4.8-8.7)	2 (1-2)	3.8 (3.4-4.2)	30 (27-32)	79.8 (72.3-86.8)
Caribbean	3 (3-3)	6.8 (6.0-7.9)	3 (3-3)	6.8 (6.3-7.3)	61 (55-66)	138.1 (126.3-150.1)
Central Europe	13 (11-15)	7 (6.1-8.1)	10 (9-11)	5.5 (5.1-5.7)	204 (190-214)	117.4 (109.1-122.9)
Eastern Europe	18 (15-24)	5.8 (4.8-7.4)	14 (13-15)	4.3 (4.0-4.6)	306 (280-329)	99.9 (92.2-107.0)
Western Europe	69 (60-83)	9 (7.9-10.4)	49 (46-51)	6.1 (5.8-6.5)	826 (772-868)	118.8 (111.3-124.9)
Andean Latin America	3 (2-4)	7.3 (5.8-8.7)	3 (3-4)	7.8 (6.4-8.7)	67 (55-75)	149.7 (122.8-167.4)
Central Latin America	12 (11-14)	6.7 (6.2-7.8)	13 (12-13)	7.3 (7.0-7.6)	268 (259-278)	138.6 (134.0-143.7)
Southern Latin America	4 (3-4)	4.9 (4.3-5.7)	4 (3-4)	5.1 (4.7-5.5)	69 (64-73)	98.6 (91.8-105.3)
Tropical Latin America	10 (8-12)	5.4 (4.6-6.8)	11 (10-12)	5.8 (5.4-6.7)	238 (220-277)	119.8 (110.8-139.0)
North Africa and Middle East	21 (18-23)	6.3 (5.5-6.9)	24 (21-26)	7.1 (6.3-7.8)	616 (502-683)	159.3 (133.2-176.0)
North America, high income	40 (33-52)	7.8 (6.5-9.7)	26 (24-26)	5 (4.8-5.2)	538 (512-560)	108.7 (103.5-113.1)
Oceania	1 (0-1)	10.8 (7.7-15.4)	1 (0-1)	11.4 (8.3-15.6)	20 (13-29)	276.5 (189.7-399.0)
Central Sub-Saharan Africa	7 (5-11)	16.9 (10.6-25.9)	8 (5-13)	19.9 (12.6-30.3)	234 (140-373)	460.1 (278.1-723.4)
East Sub-Saharan Africa	17 (13-21)	10.3 (7.9-12.8)	19 (14-24)	11.9 (9.0-15.0)	575 (433-750)	306.3 (231.1-396.2)
Southern Sub-Saharan Africa	4 (3-5)	8.6 (7.0-10.6)	4 (4-5)	9.5 (7.9-11.4)	114 (94-145)	218.3 (180.6-272.1)
West Sub-Saharan Africa	29 (22-39)	16.9 (13.0-22.4)	31 (24-41)	18.1 (14.1-23.4)	1055 (804-1422)	483.9 (372.3-640.7)

Abbreviations: ASIR, age-standardized incidence rate; ASMR, age-standardized mortality rate; DALY, disability-adjusted life-year; UI, uncertainty interval.

The highest burden of liver cancer incident cases, deaths, and DALYs was observed in East Asia. High-income Asia Pacific had the second most incident cases but only the third highest number of deaths and the fourth highest number of DALYs. Within high-income Asia Pacific, Japan was the driver behind this finding with 75% of incident cases of which 67% were due to HCV. Western Europe ranked third for liver cancer incident cases, fourth for liver cancer deaths, and fifth for total DALYs. Southeast Asia experienced the fourth highest number of incident liver cancer cases but ranked second for liver cancer deaths and DALYs. The highest ASIR in 2015 were in high-income Asia Pacific, followed by East Asia and Western sub-Saharan Africa (Table).

### Time Trends Between 1990 and 2015

**Figure 1.**
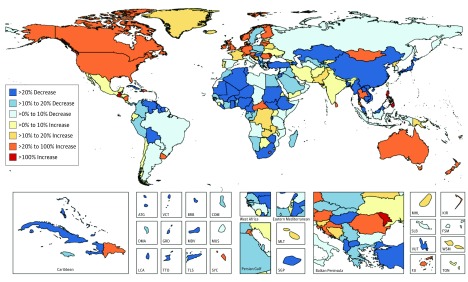
Relative Changes in Age-Standardized Liver Cancer Mortality Between 1990 and 2015 for Both Sexes in 195 Countries and Territories ATG indicates Antigua and Barbuda; BRB, Barbados; COM, Comoros; DMA, Dominica; FJI, Fiji; FSM, Federated States of Micronesia; GRD, Grenada; KIR, Kiribati; LCA, Saint Lucia; MDV, Maldives; MHL, Marshall Islands; MLT, Malta; MUS, Mauritius; TLS, Timor-Leste; TON, Tonga; TTO, Trinidad and Tobago; SGP, Singapore; SLB, Soloman Islands; SYC, Seychelles; VCT, Saint Vincent and the Grenadines; VUT, Vanuatu; and WSM, Samoa (formerly Western Samoa).

Between 1990 and 2015, liver cancer incident cases increased by 75% (eFigure 5 in Supplement 1) with changing age structures contributing 47%, population growth contributing 35%, and changing age-specific incidence rates contributing −8% to the overall increase. Because of decreases in age-specific incidence rates for HBV-related liver cancer, and liver cancer due to other causes, incident cases due to HBV would have decreased by 35% and liver cancer due to other causes by 25% between 1990 and 2015, if population size and age structure had remained the same. However, owing to demographic changes of population growth and aging, incident cases increased by 42% and 56%, respectively. For HCV-related and alcohol-related liver cancer incidence, demographic changes, as well as increases in age-specific rates, led to the overall increase of 114% and 109%, respectively (Figure 1; eFigure 5 in Supplement 1).

**Figure 2.**
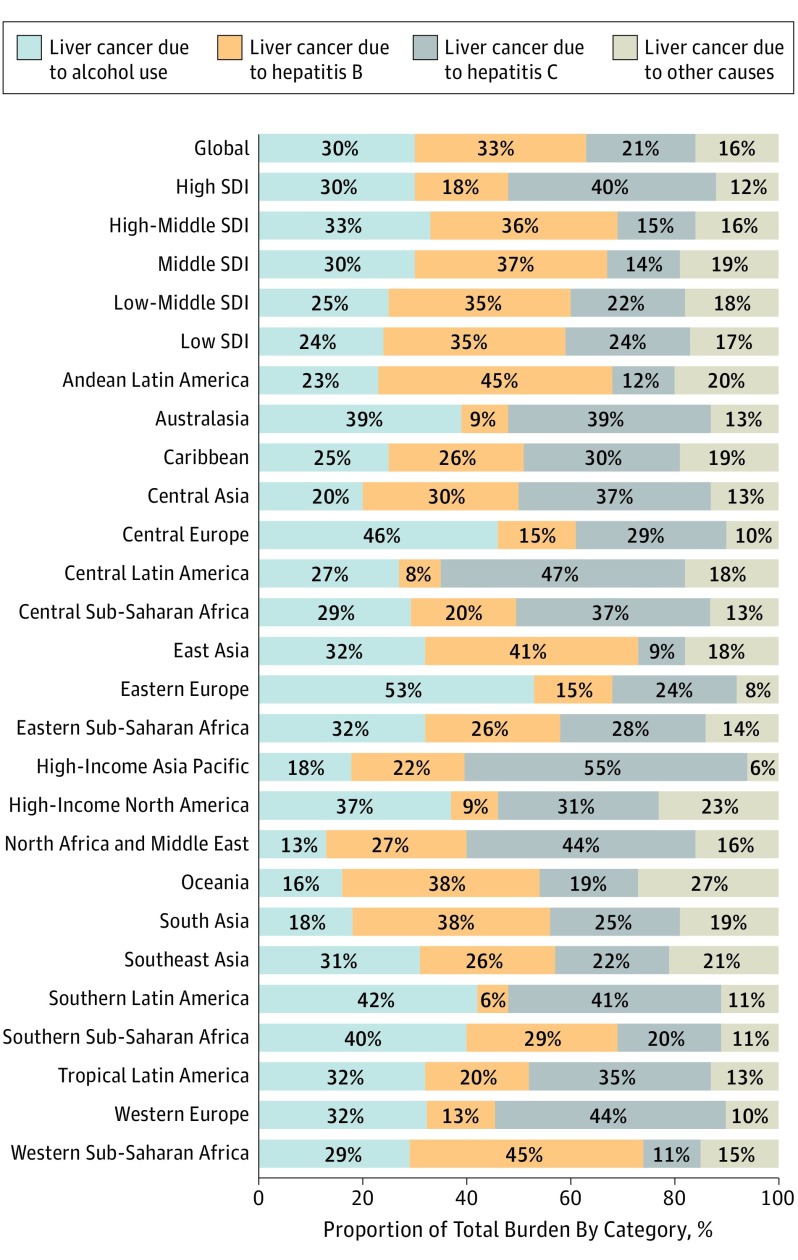
Contribution of Hepatitis B, Hepatitis C, Alcohol, and Other Causes on Absolute Liver Cancer Deaths, Both Sexes, Globally and by Region, 2015 SDI indicates sociodemographic index.

The pattern of change for ASIR between 1990 and 2015 shows a substantial increase of over 100% in many high-SDI countries like the United States, Canada, Australia, New Zealand, and most European countries, but also in the Philippines, Guatemala, Romania, and the Seychelles. At the same time, some countries with high incidence rates like China and countries in Western and Eastern sub-Saharan Africa have experienced a decrease of over 20% in ASIR (eFigure 5 in Supplement 1). Age-standardized mortality rates (ASMR) between 1990 and 2015 more than doubled in the Philippines, Moldova, and Guatemala. During the same period, ASMR declined substantially in regions with high liver cancer burden such as East Asia and Western sub-Saharan Africa (Figure 2).

### Sex Differences

Only 14% of studies used in our analysis of liver cancer etiologies reported underlying causes by sex. Using this information on sex differences, liver cancer was more common in men, with 591 000 incident cases compared with women with 264 000 cases. Similar patterns were observed for mortality (577 000 in men vs 234 000 in women) and DALYs (15 413 000 in men vs 5 165 000 in women) (Table).

At the global level, the male-to-female ratios for ASIR and ASMR rates were 2.5 and 2.8, respectively, while the male-to-female ratio was 3.1 for age-standardized DALY rates (Table). The male-to-female ratio for ASIR was highest in East Asia at 2.9 and lowest in Andean Latin America at 0.9 (eTable 8 in Supplement 1).

Marked differences at the global level exist by sex for HBV-related and alcohol-related liver cancer. In 2015, HBV caused 203 000 (95% UI, 171 000-251 000) incident liver cancer cases in men, but only 70 000 (95% UI, 57 000-86 000) cases in women. Alcohol caused 204 000 (95% UI, 177 000-240 000) liver cancer cases in men, but only 45 000 (95% UI, 38 000-54 000) cases among women in 2015 (eTable 1 in Supplement 1).

### Liver Cancer Burden by Cause Group

In 2015 at the global level, HBV was the leading cause of incident cases of liver cancer, deaths, and DALYs, followed by alcohol (eTable 9 in Supplement 1).

Between 1990 and 2015, cases of liver cancer, deaths, and DALYs increased for all cause groups globally. The highest increase in incident cases was due to HCV, followed by alcohol.

Between 1990 and 2015, ASIR for liver cancer due to HBV decreased by 18.9% (not statistically significant). During the same time, liver cancer ASIR due to HCV increased significantly by 15.7%. For liver cancer due to alcohol and other causes, ASIR did not change significantly at the global level (eTable 9 in Supplement 1).

The contribution of different etiologies to total liver cancer deaths varies markedly between countries and regions (eTable 10 in Supplement 1). At the global level in 2015, HBV was responsible for 33% of liver cancer mortality; alcohol, 30%; HCV, 21%; and other causes, 16%. Hepatitis B infection was the least common cause of liver cancer deaths in Southern Latin America at 6% and the most common in Western sub-Saharan Africa and Andean Latin America at 45%. Hepatitis C virus infection was the least common cause of liver cancer deaths in East Asia (9%) and the most common cause in the high-income Asia Pacific region (55%). The contribution of alcohol was lowest, at 13%, in North Africa and the Middle East, and highest in Eastern Europe at 53%. The etiological subgroup “other causes” was the least common cause for liver cancer deaths in high-income Asia Pacific at 6% and the most common cause in Oceania at 27%. At the country level in 2015, HBV contributed the largest proportion to liver cancer mortality in Gambia, at 60%, and the smallest in Mexico, at 4%. Hepatitis C virus infection contributed the most to liver cancer mortality in Japan, at 69%, and the least in Senegal, at 7%. Alcohol was the largest contributor to liver cancer mortality in 2015 in Belarus and the smallest contributor in Iran. Liver cancer due to other causes was the largest contributor to overall liver cancer mortality in Indonesia, at 39%, and the smallest contributor in South Korea, at 5% (eTable 10 in Supplement 1).

## Discussion

As part of the GBD 2015 study, we estimated the burden of liver cancer due to the main causes at the global, regional, and national levels to inform strategic planning of prevention programs, as well as research and health system resource allocation. Our results are in line with previous studies showing that liver cancer is among the leading causes of cancer deaths worldwide and that the liver cancer etiologies differ substantially between locations.^21^ However, whereas prior studies have focused on single aspects of liver cancer epidemiology like incidence trends in selected countries, or cross-sectional analyses of risk factors, the GBD provides a comprehensive analysis of all countries, over time, for the most important etiologies.

Analyzing time trends allows for identification of successful strategies as well as concerning patterns. At the global level, our decomposition analysis shows that liver cancer incident cases owing to HBV and other causes would have decreased between 1990 and 2015 if the demographic profile and population size had remained the same. In the same scenario, liver cancer caused by HCV and alcohol would have increased because of a rise in age-specific rates. These findings highlight 2 important issues. First, primary liver cancer prevention through HBV vaccination is starting to show successes; and second, health care systems not only have to invest in prevention but also have to plan for the increasing number of patients with liver cancer that they will face despite prevention programs.

Obvious targets for primary prevention include liver cancers due to HBV and HCV. Assuming that present HBV vaccination trends continue, between 2020 and 2050, the number of new HBV infections is estimated to drop by 70%. The reduction of chronic infections would be even larger if HBV birth dose vaccination would increase from the current level of 39% to the 2030 target of 90%.^6^ Other recommended approaches include implementation of safe injection and transfusion practices, improved diagnoses of chronic infections, and increased treatment for HBV and HCV.^6^ Our findings show that not just population aging and growth but also increasing incidence rates of liver cancer due to HCV are driving the overall rise in HCV-related liver cancer. This stresses the importance of prevention as well as accessibility and affordability of the highly effective HCV antiviral medication.^25^

For liver cancer due to alcohol, our analysis showed increasing age-specific incidence rates between 1990 and 2015, highlighting the need for strategies to decrease the harmful use of alcohol.^27^

Liver cancer due to other causes showed the smallest increase in incident cases among the 4 etiologies and decreasing age-specific rates in our decomposition analysis. This finding, however, masks etiologies that are currently included in the “other” group and that can be leading causes in certain locations, like liver flukes in Asia and Eastern Europe; aflatoxin in Asia, parts of Africa, and Latin America; and nonalcoholic steatohepatitis in South America and the Middle East.^29^

At the national level, some countries have made progress in reducing liver cancer burden over the past decades, as previously documented.^2^ China, for example, experienced a significant decrease in the ASMR of 33% (95% UI, −40.2% to −17.6%) between 1990 and 2015, possibly owing to reduced aflatoxins exposure and to some extent due to national HBV vaccination programs.^32^ Despite this decrease in ASMR, the number of deaths due to liver cancer in China increased by 33.8% between 1990 and 2015. In neighboring Mongolia no apparent progress has been made during this time frame and liver cancer remains the leading cause of cancer deaths with an increase of 171% between 1990 and 2015.^16^

Among high-SDI countries, the United States, Canada, and Australia stand out with a greater than 20% increase in liver cancer ASMR between 1990 and 2015. In the United States this is partially due to the cohort effect of high HCV prevalence among adults born between 1945 and 1965 associated with injection drug use and transfusion of unsafe blood products.^9^ Liver cancer ASMR due to alcohol, other causes, and HBV also increased in the United States between 2005 and 2015. Increasing liver cancer due to HBV in the United States and other high net-migration countries can in part be attributed to the burden of undiagnosed and untreated HBV infection in migrants from high-prevalence settings infected since early childhood.^37^ Hepatitis B testing and treatment in these populations, despite being cost-effective, has yet to be widely adopted.^38^

Even with successful primary prevention strategies, cases of liver cancer are likely to increase over the next decades owing to population aging and growth. Secondary prevention is therefore equally as important. The resource-stratified guidelines for hepatocellular carcinoma management by the National Comprehensive Cancer Network recommend screening high-risk groups with liver ultrasonography and α-fetoprotein blood tests at all levels of resources.^39^ Secondary prevention is only indicated and ethically appropriate if treatment is available. Health system planning therefore needs to focus on ensuring availability of imaging including interventional radiology, pathology, surgical and palliative care specialties. Given the disappointing results of systemic hepatocellular carcinoma treatments, research on new approaches is urgently needed. Treatment for advanced disease has been especially unsatisfactory with sorafenib being the only approved drug but leading to only modest survival benefits.^40^ Developments in immunotherapy are promising and might be feasible to use even in the setting of limited health care resources if treatment is affordable.^41^

### Limitations

The GBD estimates, as well as estimates from other groups like Globocan, depend on the quality and quantity of data used in the modeling.^8^ The wide geographic variation in the availability of high-quality cause of death and cancer registry data are reflected in the uncertainty associated with the GBD estimates. It is encouraging that despite data scarcity and different estimation methods, most Globocan estimates fall within the 95% UIs of the GBD estimates (eTable 11 in Supplement 1).

The main data quality issues for the liver cancer burden estimation are miscoding of liver metastases as primary liver cancers, underreporting of liver cancer on death certificates, and underestimation of liver cancer due to lack of diagnostic capacity.^42^ The methodological framework of the GBD tries to account for these difficulties. Redistribution of undefined causes of death or cancer to the most likely underlying cause accounts for underdiagnosis.^44^

For the etiological attribution of the liver cancer burden, HBV and HCV related cases are less prone to misclassification based on the use of objective laboratory assessments, in contrast to self-reported data for alcohol use. A caveat when comparing studies that exclusively examined viral risk factors to our study is that if more than 1 cause was reported, we apportioned coexposures to multiple causes proportionally between the causes. When information was only available for viral risk factors, the proportions of liver cancer owing to alcohol and other causes in these locations are based on covariates used in the proportion models. This can lead to underestimation of viral etiologies, as is the case for liver cancer due to HBV in Taiwan, for example, where the proportion of 27% is lower than published studies.^45^

Sex differences in the liver cancer etiologies was only available for a limited number of studies. The validity of applying these patterns to all studies is therefore unclear.

For GBD 2015 we assessed only the major liver cancer etiologies. For future iterations of the GBD, inclusion of additional etiologies as well as estimating the burden of cholangiocarcinoma and hepatocellular carcinoma separately should be considered.

## Conclusions

Liver cancer remains a major public health burden globally. The major causes for liver cancer are highly preventable or treatable. In line with the Sustainable Development Goals, the Global Health Sector Strategy on Viral Hepatitis 2016 to 2021, and the World Health Organization Global Strategy to Reduce Harmful Use of Alcohol, concerted prevention efforts will be required to achieve a sustained reduction in liver cancer. The GBD study provides the most current overview of the burden and etiology of liver cancer and can guide investments in targeted liver cancer prevention efforts.

## Notes

Supplement 1. **eAppendix.** The burden of primary liver cancer and underlying 2 etiologies from 1990 to 2015 at the global, regional, and national level: 3 results from the Global Burden of Disease Study 2015.

Supplement 2. **Web Tables.** Incidence, Mortality, and DALYs (absolute numbers and rates) by sex, 1990-2015.
