# Supplementary material for: The Burden of Primary Liver Cancer and Underlying Etiologies From 1990 to 2015 at the Global, Regional, and National Level: Results From the Global Burden of Disease Study 2015
Source: JAMA Oncol. 2017 Oct 5;3(12):1683–91. doi: 10.1001/jamaoncol.2017.3055 (PMC5824275; doi:10.1001/jamaoncol.2017.3055)
Supplement: Supplement 1. — eAppendix. The burden of primary liver cancer and underlying 2 etiologies from 1990 to 2015 at the global, regional, and national level: 3 results from the Global Burden of Disease Study 2015. [file jamaoncol-3-1683-s001.pdf]

Supplementary Online Content

Global Burden of Disease Liver Cancer Collaboration. The burden of primary liver cancer and underlying etiologies from 1990 to 2015 at the global, regional, and national level: results from the Global Burden of Disease Study 2015. Published online October 5, 2017. *JAMA Oncology*.
doi:10.1001/jamaoncol.2017.3055

eAppendix. The burden of primary liver cancer and underlying 2 etiologies from 1990 to 2015 at the global, regional, and national level: 3 results from the Global Burden of Disease Study 2015.

This supplementary material has been provided by the authors to give readers additional information about their work.

©2017 Global Burden of Disease Liver Cancer Collaboration. *JAMA Oncology*.

Web Appendix - The burden of primary liver cancer and underlying etiologies from 1990 to 2015 at the global, regional, and national level: results from the Global Burden of Disease Study 2015

©2017 Global Burden of Disease Liver Cancer Collaboration. *JAMA Oncology*.

Contents

Appendix A - Methods 4
Definition of indicator 4
Data sources 4
Cancer incidence data sources 4
Mortality to incidence ratio data sources 4
Cancer mortality data sources 4
Bias of categories of input data 4
Data analysis 5
Cancer registry data formatting 5
Mortality to incidence ratio estimation 6
Cause of death database formatting 8
CODEm models 8
CodCorrect 8
Etiology splits 8
Incidence estimation 9
Prevalence and YLD estimation 9
YLL and DALY estimation 10
Probability of cancer 10
Comparative risk assessment 10
References 11
Tables and Figures 13
See appendix: Definition of indicator 13
Appendix B – Additional results 73

©2017 Global Burden of Disease Liver Cancer Collaboration. *JAMA Oncology*.

Tables

eTable 1: GATHER checklist 13
eTable 2: Data-rich countries including number of years with 95% completeness of vital registration system data 15
eTable 3: Covariates selected for CODEm for liver cancer and expected direction of covariate 17
eTable 4: Results for CODEm model testing 18
eTable 5: References for etiology splits by country 18
eTable 6: Disability weights including uncertainty intervals 55
eTable 7: Socio-demographic Index groupings by geography, based on 2015 values 56
eTable 8: Age-standardized incidence rates with 95% uncertainty interval and male to female ratio by region, by sex, 2015 73
eTable 9: Global liver cancer incident cases, age-standardized incidence rate, deaths, age-standardized mortality rate, DALYs, and age-standardized DALYs attributable to each cause group by year and percent change (both sexes) 76
eTable 10: Contribution of hepatitis B, C, alcohol and other causes on absolute liver cancer deaths, 2015, both sexes, by location 78
eTable 11: Comparison between liver cancer deaths estimates for GBD and Globocan for the year 2012, both sexes combined 83

Figures

eFigure 1: Flowchart, liver cancer mortality estimation 70
eFigure 2: Flowchart, liver cancer incidence to DALY estimation 71
eFigure 3: Sociodemographic Index quintiles, 2015 72
eFigure 4: Liver cancer ranking by total incidence and mortality based on global level, SDI quintiles, regions, and all countries, both sexes, 2015 92
eFigure 5: Decomposition of changes in liver cancer incident cases 1990 to 2015 by cause group, both sexes 93
eFigure 6: Relative changes in age-standardized liver cancer incidence between 1990 and 2015 for both sexes in 195 countries and territories 94

©2017 Global Burden of Disease Liver Cancer Collaboration. *JAMA Oncology*.

Appendix A - Methods

Definition of indicator

In this publication estimates for liver cancer, for both sexes, for the time from 1990 to 2015 are presented for 195 countries or territories. All ICD9 and ICD10 codes pertaining to liver cancer (155-155.9 and C22, respectively) are being included in these estimates. Countries and territories reported can be found in eTable 2.

Data sources

Cancer incidence data sources

Cancer incidence was sought from individual cancer registries or aggregated databases of cancer registry data like "Cancer Incidence In Five Continents (CI5)",[1–10], EUREG,[11] or NORDCAN.[12] Data were excluded if they were not representative of the coverage population (e.g., hospital-based registries), if they did not cover all malignant neoplasms as defined in ICD9 (140-208) or ICD10 (C00-C96) (e.g. specialty cancer registry), if they did not include data for both sexes and all age groups, if the data were limited to years prior to 1980, or if the source did not provide details on the population covered. Preference was given to registries with national coverage over those with only local coverage, except those from countries where the GBD study provides subnational estimates. A list of the cancer registries included in our analysis and the years covered can be found elsewhere.[13] Additional metadata for each source are available in the online GBD citation tool http://ghdx.healthdata.org/gbd-2015-data-citations. For liver cancer 10,118 site-years were included from vital registration data and 2,419 site-years from cancer registries.

Mortality to incidence ratio data sources

Most cancer registries only report cancer incidence. However, if a cancer registry also reported cancer mortality, mortality data were extracted from the source to be used in the mortality to incidence estimation. In the case when high-quality mortality data were available but not reported by the registry, processed (post-redistribution, see below) vital registration mortality data from the cause of death database were matched to the registry's incidence data. This was the case for certain registries in the following countries: Australia, Austria, Belgium, Bulgaria, Denmark, Estonia, Finland, Hungary, Iceland, Ireland, New Zealand, Norway, South Korea, and Switzerland.

Cancer mortality data sources

A detailed description of the data sources and processing steps for the cause of death database can be found in the appendix to the paper "Global, regional, and national life expectancy, all-cause and cause-specific mortality for 249 causes of death, 1980–2015: a systematic analysis for the Global Burden of Disease Study 2015."[14]

Bias of categories of input data

Bias of the input data included for the COD database is described elsewhere.[14] Cancer registry data can be biased in multiple ways. A high proportion of ill-defined cancer cases in the registry data requires redistribution of these cases to other cancers, which introduces a potential for bias. Changes between coding systems can lead to artificial differences in disease estimates; however, we adjust for this bias by mapping the different coding systems to the GBD causes. Underreporting of cancers that require advanced diagnostic techniques (e.g., leukemia and brain, pancreatic, and liver cancers) can be an issue in cancer registries from low-income countries. On the other hand, misclassification of metastatic sites

©2017 Global Burden of Disease Liver Cancer Collaboration. *JAMA Oncology*.

as primary cancer can lead to overestimation of cancer sites that are common sites for metastases like brain or liver. Since many cancer registries are located in urban areas, the representativeness of the registry for the general population can also be problematic. The accuracy of mortality data reported in cancer registries usually depends on the quality of the vital registration system. If the vital registration system is incomplete or of poor quality, the mortality to incidence ratio can be biased to lower ratios.

Data analysis

Flowcharts describing the conceptual overview of the data processing are available in eFigure 1 and eFigure 2.

Cancer registry data formatting

Cancer registry data went through multiple processing steps before integration with the COD database. First, the original data were transformed into standardized files, which included standardization of format, categorization, and registry names (#1 in eFigure 1).
Second, some cancer registries report individual codes as well as aggregated totals (e.g., C18, C19, and C20 are reported individually, but the aggregated group of C18-C20 (colorectal cancer) is also reported in the registry data). The data processing step "subtotal recalculation" (#2 in flowchart) verified these totals and subtracted the values of any individual codes from the aggregates.
In the third step (#3 in the flowchart), cancer registry incidence data and cancer registry mortality data were mapped to GBD causes. A different map is used for incidence and for mortality data. One example is ICD10 D13.4 (Benign neoplasm of liver) and ICD9 211.5 (Benign neoplasm of liver and biliary passages), which are mapped to "liver cancer" in vital registration system data but dropped from any incidence data. The assumption is that deaths assigned to benign neoplasms are miscoded and should correctly be assigned to the invasive cancer.
In the fourth data processing step (#4 in the flowchart) cancer registry data were standardized to the GBD age groups. Age-specific incidence rates were generated using CI5, SEER, and NORDCAN data, while age-specific mortality rates were generated from the CoD data.[14] Age-specific weights were then generated by applying the age-specific rates to a given registry population that required age-splitting to produce the expected number of cases/deaths for that registry by age. The expected number of cases/deaths for each sex, age, and cancer were then normalized to 1, creating final, age-specific proportions. These proportions were then applied to the total number of cases/deaths by sex and cancer to get the age-specific number of cases/deaths.
In the rare case that the cancer registry only contained data for both sexes combined, the age-specific cases/deaths were split and reassigned to separate sexes using the same weights that are used for the age-splitting process. Starting from the expected number of deaths, proportions were generated by sex for each age (e.g., if for ages 15-19 years old there are six expected deaths for males and four expected deaths for females, then 60% of the combined-sex deaths for ages 15-19 years would be assigned to males and the remaining 40% would be assigned to females).
In the fifth step (#5 in the flowchart) data for cause entries that are aggregates of GBD causes were redistributed. Examples of these aggregated causes include some registries reporting ICD10 codes C00-C14 together as, "lip, oral cavity, and pharyngeal cancer." These groups were broken down into subcauses that could be mapped to single GBD causes. In this example, those include lip and oral cavity cancer (C00-C08), nasopharyngeal cancer (C11), cancer of other parts of the pharynx (C09-C10, C12-C13), and "Malignant neoplasm of other and ill-defined sites in the lip, oral cavity and pharynx" (C14). To redistribute the data, weights were created using the same method employed in age-sex splitting (see

©2017 Global Burden of Disease Liver Cancer Collaboration. *JAMA Oncology*.

step four above). For the undefined code (C14 in the example) an “average all-cancer” weight was used, which was generated by adding all cases from SEER/NORDCAN/CI5 and dividing those by the combined population. Then, proportions were generated by subcause for each aggregate cause as in the sex-splitting example above (see step four). The total number of cases from the aggregated group (C00-C14) was recalculated for each subgroup and the undefined code (C14). C14 was then redistributed as a “garbage code” in step six. Distinct proportions were used for C46 (Kaposi sarcoma). C46 entries were redistributed as “other cancer” and HIV.

In the sixth step (#6 in the flowchart) unspecified code (“garbage code”) was redistributed. Redistribution of cancer registry incidence and mortality data mirrored the process of the redistribution used in the cause of death database and has not changed compared to GBD 2013.[14]

In the seventh step (#7 in the flowchart) duplicate or redundant sources were removed from the processed cancer registry dataset. Duplicate sources were present if, for example, the cancer registry was part of the CI5 dataset but we also had data from the registry directly. Redundancies occurred and were removed as described in “Inclusion and Exclusion Criteria,” where more detailed data were available, or when national registry data could replace regionally representative data. From here, two parallel selection processes were run to generate input data for the MI models and to generate incidence for final mortality estimation. Higher priority was given to registry data from the most standardized source when creating the final incidence input (generally CI5 data); whereas preference was given to registry data from sources with matching mortality and incidence for the MI model input.

Mortality to incidence ratio estimation

In the eighth step (#8 in the flowchart) the processed liver cancer incidence and mortality data from cancer registries were matched by cancer, age, sex, year, and location to generate MI ratios. Because some cancer registries do not report mortality data – even though high-quality vital registration system data are available for the registry’s coverage area –processed vital registration mortality data from the CoD database were matched to the registry’s incidence data for some countries. This was the case for certain registries in the following countries: Australia, Austria, Belgium, Bulgaria, Denmark, Estonia, Finland, Hungary, Iceland, Ireland, New Zealand, Norway, South Korea, and Switzerland.

The ninth step involved creating and selecting the MI models. Multiple logit random effect models were created. All models were run separately for liver cancer, and the best model was selected from the following list.

1. $\text{logit}\left(MI\ ratio_{c,a,s,t}\right) = \alpha + \beta_1 SDI_{c,t} + \sum_a^A \beta_2 I_a + \beta_3 I_s + \theta_c + \epsilon_{c,a,s,t}$
2. $\text{logit}\left(MI\ ratio_{c,a,s,t}\right) = \alpha + \beta_1 SDI_{c,t} + \sum_a^A \beta_2 I_a + \beta_3 I_s + \beta_4 t + \theta_c + \epsilon_{c,a,s,t}$
3. $\text{logit}\left(MI\ ratio_{c,a,s,t}\right) = \alpha + \beta_1 SDI_{c,t} + \sum_a^A \beta_2 I_a + \beta_3 I_s + \beta_4 DS + \theta_c + \epsilon_{c,a,s,t}$
4. $\text{logit}\left(MI\ ratio_{c,a,s,t}\right) = \alpha + \beta_1 SDI_{c,t} + \sum_a^A \beta_2 I_a + \beta_3 I_s + \beta_4 DS + \beta_5 t + \theta_c + \epsilon_{c,a,s,t}$
5. $\text{logit}\left(MI\ ratio_{c,a,s,t}\right) = \alpha + \beta_1 SDI_{c,t} + \sum_a^A \beta_2 I_a + \beta_3 I_s + \theta_c + \lambda_{SR}(SDS_{c,t}) + \beta_4 t + \epsilon_{c,a,s,t}$
6. $\text{logit}\left(MI\ ratio_{c,a,s,t}\right) = \alpha + \beta_1 SDI_{c,t} + \sum_a^A \beta_2 I_a + \beta_3 I_s + \theta_c + \lambda_{SR}(SDS_{c,t}) + \epsilon_{c,a,s,t}$
7. $\text{logit}\left(MI\ ratio_{c,a,s,t}\right) = \alpha + \beta_1 SDI_{c,t} + \sum_a^A \beta_2 I_a + \beta_3 I_s + \theta_c + \lambda_{SR}(SDS_{c,t}) + \beta_4 DS + \epsilon_{c,a,s,t}$
8. $\text{logit}\left(MI\ ratio_{c,a,s,t}\right) = \alpha + \beta_1 SDI_{c,t} + \sum_a^A \beta_2 I_a + \beta_3 I_s + \theta_c + \lambda_{SR}(SDS_{c,t}) + \beta_4 t + \beta_5 DS + \epsilon_{c,a,s,t}$

c: country, a: age group, t: time (years); s: sex

SDI: Socio-demographic Index (index using log lag dependent income per capita (LDI), average educational attainment in the population over age 15, and total fertility rate (TFR))

©2017 Global Burden of Disease Liver Cancer Collaboration. *JAMA Oncology*.

I: indicator variable
DS: binary variable for development status
θ_c: random effect by country (intercept)
$\lambda_{SR}(SDI_{c,t})$: random effect modifier between SDI and superregion (slope)
$\epsilon_{c,a,s,t}$: error term

All models were tested at multiple stages before creating the final model output. Models were initiated with SDI (Socio-demographic Index) as covariate and first tested using the complete input dataset.[14] If after that initial test the SDI covariate's coefficient was negative (as expected), the next step was to outlier any data point for which the residual from the prediction was greater than three times the MAD from the mean residual. Next, data were marked as outliers due to a random effect criterion: if the country-level random effect for a developing country was lower than the random effect for the USA, all data points for that country were marked as outliers. The rational of choosing the US was that the MI ratio is expected to be lower in the US compared to a developing country. This process was run iteratively until all developing countries had country-level random effects greater than that of the USA. All data points marked outliers were dropped from the final dataset, and that dataset was used to create the final model predictions.

If the SDI coefficient was found to be positive (unexpected) after the initial SDI test, it was assumed to indicate an excess of unrealistic data in the input dataset. To remove these unrealistic data, SDI was temporarily removed from the model formula. The model proceeded as above without SDI until all unrealistic data points were removed and the SDI coefficient was found to be negative. Unrealistic data were marked as outliers using the same residual MAD and random effect methodology described above. Once SDI was established as negative (expected) the model proceeded as usual.

To select the best model formula, the initial model results were tested by comparing mean MI predictions and the mean root-mean-squared error (RMSE) values of 10 random samples of 80%/20% splits from the input dataset. Mean MI predictions were compared between developing and developed countries. Models were eliminated if the mean MI for developed countries was higher than the mean MI ratio for developing countries. For RMSE testing, the dataset was split into an 80% dataset for model development and a 20% dataset for model testing. The process was repeated 10 times. The best model for liver cancer was selected based on the lowest mean out-of-sample RMSE from those models remaining after checking the mean MI:

$$\text{logit}\left(MI\ ratio_{c,a,s,t}\right) = \alpha + \beta_1 SDI_{c,t} + \sum_{a}^{A} \beta_2 I_a + \beta_3 I_s + \beta_4 DS + \beta_5 t + \theta_c + \epsilon_{c,a,s,t}$$

Once the best models were selected, data points were manually outliered based on the results of the first run of the model algorithm. Data points were outliered if they clearly influenced the model in an unrealistic way. For example, a data point was marked as an outlier if it created a single-year, single-age-group spike in model predictions. This was mainly the case in countries with a small number of cases or deaths, or in age groups with small numbers of cases or deaths. Manual outliers were removed from the input dataset prior to initiating the second run of the model algorithm.

After best models were selected, all final outliers were dropped from the data input, and final linear predictions were created; the final linear predictions and residuals were used as input for space-time smoothing. Space-time smoothing is a spatiotemporal regression to smooth residuals over space, time, and age.[15] The weighted residuals were added to the linear model predictions and used as priors for the

©2017 Global Burden of Disease Liver Cancer Collaboration. *JAMA Oncology*.

third stage, a Gaussian process regression (GPR) implementing a Matern covariance function.[16,17] GPR is a nonparametric technique for interpolating non-linear trends that has been used extensively in the estimation of time series data. Final MI ratio predictions with 95% uncertainty intervals were obtained by back-transforming 1,000 draws from the posterior distribution.

Step 9 has undergone a revision compared to GBD 2010 and GBD 2013. Whereas in GBD 2010 and GBD 2013 only one model was used to predict all MI ratios, for GBD 2015 we generated multiple models and chose a best model based on out-of-sample validation. Another major difference is that LDI (lagged distributed income) was used as a covariate, which was replaced by SDI (Socio-demographic Index) for GBD 2015.

Final MI ratios were matched with the cancer registry incidence dataset in the ninth step (#10 in the flowchart) to generate mortality estimates (Incidence * modeled Mortality/Incidence = Incidence-based mortality estimates). Only the mean of estimated MI ratios was used to transform the incidence data to mortality estimates due to the current inability in the COD database to incorporate uncertainty other than sample size and representativeness. This leads to an underestimation of the uncertainty for the incidence-based mortality estimates from cancer registries. The final mortality estimates were then added to the COD database (#11 in the flowchart).

Cause of death database formatting

Formatting of data sources for the cause of death database has been described in detail elsewhere.[14]

CODEm models

Mortality estimates for liver cancer were generated using CODEm. Methods describing the CODEm approach have been described elsewhere.[14,18] In brief, the CODEm modeling approach is based on the principles that all types of available data should be used even if data quality varies; that individual models but also ensemble models should be tested for their predictive validity; and that the best model or sets of models should be chosen based on the out-of-sample predictive validity. Models were run separately for countries with extensive and complete vital registration data and countries with less VR data to prevent an inflation in the uncertainty around the estimates in “data-rich” countries. eTable 4 includes the list of data-rich countries. Covariates were selected based on a possible predictive relationship between the covariate and the specific cancer mortality. Level 1 covariates have a proven strong relationship with the outcome such as etiological or biological roles. Level 2 covariates have a strong relationship but not a direct biological link. Covariates, which are more distal in the causal chain or are mediated through Level 1 or 2 covariates are categorized as Level 3.[18] Differences in covariate selection between GBD 2013 and GBD 2015 can be found in eTable 3. Results for CODEm model testing can be found in eTable 5.

CodCorrect

CODEm models estimate the individual cause-level mortality without taking into account the all-cause mortality. To ensure that all single causes add up to the all-cause mortality and that all child-causes add up to the parent cause, an algorithm called “CodCorrect” is used. Details regarding the algorithm can be found elsewhere.[14]

Etiology splits

To find the proportion of liver cancer cases due to the four etiology groups included in GBD (1. Liver cancer due to hepatitis B, 2. Liver cancer due to hepatitis C, 3. Liver cancer due to alcohol, 4. Liver cancer

©2017 Global Burden of Disease Liver Cancer Collaboration. *JAMA Oncology*.

due to other causes), a systematic literature search was performed in PubMed. Results can be found in eTable 6. Studies were included if the study population was representative of liver cancer patients for the respective location. For each study the proportions of liver cancer due to the three specific risk factors were calculated. Remaining risk factors were included under a combined “other” group. Cryptogenic cases were excluded. If multiple risk factors were reported for an individual patient these were apportioned proportionally to the individual risk factors. The proportion data found through the systematic literature review were used as input for four separate DisMod-MR 2.1 models to determine the proportion of liver cancers due to the four subgroups for all locations, both sexes, all age groups. A study covariate was used for publications that only assessed liver cancer in a cirrhotic population. The reference or “gold-standard” that was used for crosswalking was the compilation of all studies that assessed the etiology of liver cancer in a general population. For liver cancer due to hepatitis C and hepatitis B, a prior value of 0 was set between age 0 and 0.01. For liver cancer due to alcohol a prior value of 0 was set for ages 0 to 5 years. For liver cancer due to hepatitis C, hepatitis C (IgG) seroprevalence was used as a covariate as well as a covariate for alcohol (liters per capita) and hepatitis B prevalence (HBsAg seroprevalence), forcing a negative relationship between the alcohol and hepatitis B covariate and the outcome of liver cancer due to hepatitis C proportion. For liver cancer due to hepatitis B, seroprevalence of HBsAg was used as a covariate as well as a covariate for alcohol and hepatitis C IgG seroprevalence, forcing a negative relationship between the alcohol and hepatitis C covariate and the outcome of liver cancer due to hepatitis B proportion. For liver cancer due to alcohol, alcohol (liters per capita) was used as a covariate as well as a covariate for hepatitis B and hepatitis C seroprevalence, forcing a negative relationship between the hepatitis B and hepatitis C covariates and the outcome of liver cancer due to alcohol proportion. All covariates used were modeled independently.[19]

Incidence estimation

GBD cancer incidence estimates were generated by dividing final liver cancer mortality estimates (after CodCorrect adjustment and etiology splits) by the liver cancer MI ratios (step 1 in eFigure 2). To propagate uncertainty from the MI ratios and the mortality estimates to incidence, this process was done at the 1,000 draw level. It was assumed that uncertainty in the MI ratio is independent of uncertainty in the estimated age-specific death rates.

Prevalence and YLD estimation

Prevalence is estimated as 10-year prevalence for liver cancer as in GBD 2013.[20] To estimate cancer prevalence, relative cancer survival was estimated by scaling cancer-specific survival between a “best case” and “worst case” survival. The methods and input data used to generate the best and worst case survival as well as to scale countries between these boundaries remained the same as for the GBD 2013 study (steps 2, 3, and 5 in the flowchart).[20] To transform relative to absolute survival (adjusting for background mortality) GBD 2015 lifetables were used (steps 6 and 7 in the flowchart).[14] The access to cancer care variable to scale countries between the best and worst case survival was estimated using the same method as for GBD 2013 (step 4 in the flowchart):[20]

$$Access\ to\ care = 1 - \frac{Age\ standardized\ MI\ ratio_{cys} - Age\ standardized\ MI\ ratio_{min}}{Age\ standardized\ MI\ ratio_{max} - Age\ standardized\ MI\ ratio_{min}}$$

c=country; y=year; s=sex; age-standardized MI ratio$_{min}$=lowest MI ratio for all countries and years; age standardized MI ratio$_{max}$=highest MI ratio for all countries and years

©2017 Global Burden of Disease Liver Cancer Collaboration. JAMA Oncology.

Duration of the treatment phases (1. Diagnosis and primary therapy; 2. Controlled phase; 3. Metastatic phase; 4. Terminal phase) remained the same as for GBD 2013 with 4 months assumed for the diagnosis and treatment phase, 2.51 months assumed for the metastatic phase[21] and 1 month for the terminal phase. Total prevalence time was divided into phases 1, 3, and 4, for the population that died within 10 years, and the remaining prevalence was attributed to the controlled phase. For the population that survived beyond 10 years, prevalence person-time was attributed to phase 1 and phase 2. Years lived with disability (YLDs) were calculated by multiplying each phase by the respective disability weight (eTable 7). To generate the total YLDs for each cancer, the YLDs for each cancer sequela were added (step 13 in eFigure 2). The standard GBD 2013 disability weights were used, which were derived from an open-access internet survey and household surveys in Bangladesh, Hungary, Indonesia, Italy, the Netherlands, Peru, Sweden, Tanzania, and the United States. In the surveys, respondents were asked to determine the healthier individual between two choices with different, randomly selected health states based on a lay description. These surveys were used to generate 235 health states reflecting all GBD sequelae.[22,23]

YLL and DALY estimation

The number of years of life lost (YLLs) due to cancer deaths was calculated by multiplying the number of deaths in each age group by the normative standard life expectancy for the corresponding age group. For GBD 2015 the normative standard life expectancy at birth was 86.59 years, which is based on the lowest observed death rates for each five-year age group in populations greater than 5 million.

Probability of cancer

The cumulative probability of developing cancer for certain age groups and an approximated lifetime risk for all cancer groups (age 0 to 79) as well as the odds of developing cancer for 2015 were calculated. The method used does not take into account competing risks of death. The cancer risk is approximated using the following formula[24]:

$$Cumulative\ risk = 1 - e^{-cumulative\ rate}$$

Comparative risk assessment

As part of the GBD study, risks leading to poor health outcomes are assessed using a comparative risk assessment (CRA) approach.[25] This approach differs from the etiological attribution of liver cancer presented in this study. Categories of risks currently included in the CRA are behavioral, environmental and occupational, and metabolic risks. For these groups, risk-outcome pairs are defined based on evidence of a causal relationship. For liver cancer the risks included in the CRA were smoking, alcohol use, and high body mass index. For liver cancer due to hepatitis B and C, drug use was included as an additional risk-outcome pair. Pathogens like hepatitis B and C have not yet been included in the CRA. The population attributable fraction (PAF) was estimated in the CRA by using the relative risk of an exposure, the prevalence of the risk in a population, and the theoretical minimum risk level. At the global level the PAF of alcohol use on liver cancer deaths due to alcohol use was 100%, meaning that all liver cancer deaths due to alcohol could have been avoided if there were 100% abstinence from alcohol at the population level. The risk factor attribution for liver cancer deaths due to hepatitis C in 2015 was 55.8% (95% UI, 46.7%–64.1%). For liver cancer due to hepatitis B it was 23.7% (95% UI, 13.8%–34.9%). For liver cancer due to other causes it was 21.5% (95% UI, 12.5%–31.0%). Further results for the attributable burden can be found elsewhere.[25]

©2017 Global Burden of Disease Liver Cancer Collaboration. JAMA Oncology.

References

1. Doll R, Payne P, Waterhouse J. *Cancer Incidence in Five Continents I*. Geneva: Union Internationale Contre le Cancer; 1966.

2. Doll R, Muir C, Waterhouse J. *Cancer Incidence in Five Continents II*. Geneva: Union Internationale Contre le Cancer, Geneva; 1970.

3. Waterhouse J, Muir C, Correa P, Powell J. *Cancer Incidence in Five Continents III*. Lyon: IARC; 1976.

4. Waterhouse J, Muir C, Shanmugaratnam K, Powell J. *Cancer Incidence in Five Continents IV*. Lyon: IARC; 1982.

5. Muir C, Mack T, Powell J, Whelan S. *Cancer Incidence in Five Continents V*. Lyon: IARC; 1987.

6. Parkin D, Muir C, Whelan S, Gao Y, Ferlay J, Powell J. *Cancer Incidence in Five Continents VI*. Lyon: IARC; 1992.

7. Parkin D, Whelan S, Ferlay J, Raymond L, Young J. *Cancer Incidence in Five Continents VII*. Lyon: IARC; 1997.

8. Parkin D, Whelan S, Ferlay J, Teppo L, Thomas D. *Cancer Incidence in Five Continents VIII*. Lyon: IARC; 2002.

9. Curado M, Edwards B, Shin H, et al. *Cancer Incidence in Five Continents IX*. Lyon: IARC; 2007. http://www.iarc.fr/en/publications/pdfs-online/epi/sp160/CI5vol9-A.pdf.

10. Forman D, Bray F, Brewster D, et al. Cancer Incidence in Five Continents X. http://ci5.iarc.fr. Published 2013.

11. Steliarova-Foucher E, O'Callaghan M, Ferlay J, Masuyer E, Forman D, Comber H, Bray F. European Cancer Observatory: Cancer Incidence, Mortality, Prevalence and Survival in Europe. International Agency for Research on Cancer. http://eco.iarc.fr. Accessed August 10, 2016.

12. Engholm G, Ferlay J, Christensen N, et al. NORDCAN--a Nordic tool for cancer information, planning, quality control and research. *Acta Oncol*. 2010;49(5):725-736. doi:10.3109/02841861003782017.

13. GBD Mortality and Causes of Death Collaborators. Global, regional, and national life expectancy, all-cause and cause specific mortality for 249 causes of death, 1980–2015: a systematic analysis for the Global Burden of Disease Study 2015. *The Lancet*. August 2016.

14. Wang H, Dwyer-Lindgren L, Lofgren KT, et al. Age-specific and sex-specific mortality in 187 countries, 1970-2010: a systematic analysis for the Global Burden of Disease Study 2010. *The Lancet*. 2012;380(9859):2071-2094. doi:10.1016/S0140-6736(12)61719-X.

15. Murray CJ, Rosenfeld LC, Lim SS, et al. Global malaria mortality between 1980 and 2010: a systematic analysis. *The Lancet*. 2012;379(9814):413-431. doi:10.1016/S0140-6736(12)60034-8.

©2017 Global Burden of Disease Liver Cancer Collaboration. JAMA Oncology.

16. Rasmussen CE, Williams CKI. *Gaussian Processes for Machine Learning*. Cambridge, Mass: MIT Press; 2006.

17. Foreman KJ, Lozano R, Lopez AD, Murray CJ. Modeling causes of death: an integrated approach using CODEm. *Popul Health Metr*. 2012;10(1):1. doi:10.1186/1478-7954-10-1.

18. Stanaway JD, Flaxman AD, Naghavi M, et al. The global burden of viral hepatitis from 1990 to 2013: findings from the Global Burden of Disease Study 2013. *The Lancet*. July 2016. doi:10.1016/S0140-6736(16)30579-7.

19. Fitzmaurice C, Dicker D, Pain A, et al. The Global Burden of Cancer 2013. *JAMA Oncol*. May 2015. doi:10.1001/jamaoncol.2015.0735.

20. *Surveillance, Epidemiology, and End Results (SEER) Program (Www.seer.cancer.gov) SEER*Stat Database: Incidence - SEER 18 Regs Research Data + Hurricane Katrina Impacted Louisiana Cases, Nov 2012 Sub (1973-2010 Varying) - Linked To County Attributes - Total U.S., 1969-2011 Counties, National Cancer Institute, DCCPS, Surveillance Research Program, Surveillance Systems Branch, Released April 2013, Based on the November 2012 Submission.*

21. Haagsma JA, Maertens de Noordhout C, Polinder S, et al. Assessing disability weights based on the responses of 30,660 people from four European countries. *Popul Health Metr*. 2015;13(1). doi:10.1186/s12963-015-0042-4.

22. Salomon JA, Vos T, Hogan DR, et al. Common values in assessing health outcomes from disease and injury: disability weights measurement study for the Global Burden of Disease Study 2010. *The Lancet*. 2012;380(9859):2129-2143. doi:10.1016/S0140-6736(12)61680-8.

23. Esteve J, Benhamou E, Raymond L. *Descriptive Epidemiology*. Vol VI. IARC Scientific Publications No.128. Lyon, France: IARC Publications; 1994.

24. Global, regional, and national comparative risk assessment of 79 behavioural, environmental and occupational, and metabolic risks or clusters of risks, 1990–2015: a systematic analysis for the Global Burden of Disease Study 2015. *The Lancet*. 2016;388(10053):1659-1724. doi:10.1016/S0140-6736(16)31679-8.

25. Global Burden of Disease Cancer Collaboration, Fitzmaurice C, Allen C, et al. Global, Regional, and National Cancer Incidence, Mortality, Years of Life Lost, Years Lived With Disability, and Disability-Adjusted Life-years for 32 Cancer Groups, 1990 to 2015: A Systematic Analysis for the Global Burden of Disease Study. *JAMA Oncol*. December 2016. doi:10.1001/jamaoncol.2016.5688.

©2017 Global Burden of Disease Liver Cancer Collaboration. JAMA Oncology.

Tables and Figures

eTable 1: GATHER checklist

**Objectives and funding**	**Reported in the manuscript/appendix**
1 Define the indicator(s), populations (including age, sex, and geographic entities), and time period(s) for which estimates were made.	See appendix: Definition of indicator
2 List the funding sources for the work.	Bill & Melinda Gates Foundation
**Data Inputs**	
**For all data inputs from multiple sources that are synthesized as part of the study:**	
3 Describe how the data were identified and how the data were accessed.	See appendix: "Data sources"
4 Specify the inclusion and exclusion criteria. Identify all ad-hoc exclusions.	See appendix: "Data sources"
5 Provide information about all included data sources and their main characteristics. For each data source used, report reference information or contact name/institution, population represented, data collection method, year(s) of data collection, sex and age range, diagnostic criteria or measurement method, and sample size, as relevant.	http://internal-ghdx.healthdata.org/gbd-2015/data-input-sources
6 Identify and describe any categories of input data that have potentially important biases (e.g., based on characteristics listed in item 5).	See appendix: "Bias of categories of input data"
**For data inputs that contribute to the analysis but were not synthesized as part of the study:**	
7 Describe and give sources for any other data inputs.	http://internal-ghdx.healthdata.org/gbd-2015/data-input-sources
**For all data inputs:**	
8 Provide all data inputs in a file format from which data can be efficiently extracted (e.g., a spreadsheet rather than a PDF), including all relevant meta-data listed in item 5. For any data inputs that cannot be shared because of ethical or legal reasons, such as third-party ownership, provide a contact name or the name of the institution that retains the right to the data.	http://internal-ghdx.healthdata.org/gbd-2015/data-input-sources
**DATA ANALYSIS**	
9 Provide a conceptual overview of the data analysis method. A diagram may be helpful.	See eFigure 1: Flowchart, liver cancer mortality estimation<br>See eFigure 2: Flowchart, liver cancer incidence to DALY estimation
10 Provide a detailed description of all steps of the analysis, including mathematical formulae. This description should cover, as relevant, data cleaning, data pre-processing, data adjustments and weighting of data sources, and mathematical or statistical model(s).	See appendix: "Data analysis"
11 Describe how candidate models were evaluated and how the final model(s) were selected.	See appendix "Mortality to incidence ratio estimation"

©2017 Global Burden of Disease Liver Cancer Collaboration. JAMA Oncology.

	See appendix “CODEm models”<br>See eTable 3: Covariates selected for CODEm for liver cancer and expected direction of covariate
12 Provide the results of an evaluation of model performance, if done, as well as the results of any relevant sensitivity analysis.	See eTable 5: Results for CODEm model testing
13 Describe methods of calculating uncertainty of the estimates. State which sources of uncertainty were, and were not, accounted for in the uncertainty analysis.	See appendix “Data analysis”
14 State how analytic or statistical source code used to generate estimates can be accessed.	http://ghdx.healthdata.org/gbd-2015-code
**RESULTS AND DISCUSSION**	
15 Provide published estimates in a file format from which data can be efficiently extracted.	GBD 2015 estimates are available online (http://vizhub.healthdata.org/gbd-compare). WebTables specific to the GBD 2015 cancer results are available online (see link in main paper).
16 Report a quantitative measure of the uncertainty of the estimates (e.g., uncertainty intervals).	Done
17 Interpret results in light of existing evidence. If updating a previous set of estimates, describe the reasons for changes in estimates.	eTable 3: Covariates selected for CODEm for liver cancer and expected direction of covariate
18 Discuss limitations of the estimates. Include a discussion of any modelling assumptions or data limitations that affect interpretation of the estimates.	See main manuscript “Limitations”

©2017 Global Burden of Disease Liver Cancer Collaboration. JAMA Oncology.

eTable 2: Data-rich countries including number of years with 95% completeness of vital registration system data

Super Region	Region	Country	Number of years with 95% completeness
Southeast Asia, East Asia, and Oceania	Southeast Asia	Mauritius	35
Southeast Asia, East Asia, and Oceania	Southeast Asia	Philippines	33
Southeast Asia, East Asia, and Oceania	Southeast Asia	Sri Lanka	26
Central Europe, Eastern Europe, and Central Asia	Central Asia	Kazakhstan	29
Central Europe, Eastern Europe, and Central Asia	Central Europe	Bulgaria	33
Central Europe, Eastern Europe, and Central Asia	Central Europe	Croatia	29
Central Europe, Eastern Europe, and Central Asia	Central Europe	Czech Republic	28
Central Europe, Eastern Europe, and Central Asia	Central Europe	Hungary	34
Central Europe, Eastern Europe, and Central Asia	Central Europe	Poland	32
Central Europe, Eastern Europe, and Central Asia	Central Europe	Romania	33
Central Europe, Eastern Europe, and Central Asia	Central Europe	Slovenia	26
Central Europe, Eastern Europe, and Central Asia	Eastern Europe	Estonia	30
Central Europe, Eastern Europe, and Central Asia	Eastern Europe	Latvia	33
Central Europe, Eastern Europe, and Central Asia	Eastern Europe	Lithuania	31
Central Europe, Eastern Europe, and Central Asia	Eastern Europe	Moldova	31
Central Europe, Eastern Europe, and Central Asia	Eastern Europe	Russia	35
Central Europe, Eastern Europe, and Central Asia	Eastern Europe	Ukraine	30
High-income	High-income Asia Pacific	Japan	34
High-income	High-income Asia Pacific	South Korea	29
High-income	High-income Asia Pacific	Singapore	35
High-income	Australasia	Australia	32
High-income	Australasia	New Zealand	33
High-income	Western Europe	Austria	35
High-income	Western Europe	Belgium	33
High-income	Western Europe	Denmark	33
High-income	Western Europe	Finland	34
High-income	Western Europe	France	32
High-income	Western Europe	Germany	34
High-income	Western Europe	Greece	33

©2017 Global Burden of Disease Liver Cancer Collaboration. JAMA Oncology.

High-income	Western Europe	Iceland	30
High-income	Western Europe	Ireland	33
High-income	Western Europe	Israel	34
High-income	Western Europe	Italy	31
High-income	Western Europe	Luxembourg	34
High-income	Western Europe	Malta	35
High-income	Western Europe	Netherlands	34
High-income	Western Europe	Norway	34
High-income	Western Europe	Portugal	32
High-income	Western Europe	Spain	34
High-income	Western Europe	Sweden	32
High-income	Western Europe	Switzerland	34
High-income	Western Europe	United Kingdom	34
High-income	Southern Latin America	Argentina	34
High-income	Southern Latin America	Chile	34
High-income	Southern Latin America	Uruguay	32
High-income	High-income North America	Canada	32
High-income	High-income North America	United States	34
Latin America and Caribbean	Caribbean	Antigua and Barbuda	28
Latin America and Caribbean	Caribbean	Barbados	29
Latin America and Caribbean	Caribbean	Bermuda	32
Latin America and Caribbean	Caribbean	Cuba	34
Latin America and Caribbean	Caribbean	Puerto Rico	33
Latin America and Caribbean	Caribbean	Saint Lucia	29
Latin America and Caribbean	Caribbean	Saint Vincent and the Grenadines	26
Latin America and Caribbean	Caribbean	Trinidad and Tobago	30
Latin America and Caribbean	Central Latin America	Colombia	31
Latin America and Caribbean	Central Latin America	Costa Rica	34
Latin America and Caribbean	Central Latin America	Guatemala	31
Latin America and Caribbean	Central Latin America	Mexico	34

©2017 Global Burden of Disease Liver Cancer Collaboration. JAMA Oncology.

Latin America and Caribbean	Central Latin America	Venezuela	30
North Africa and Middle East	North Africa and Middle East	Kuwait	29

eTable 3: Covariates selected for CODEm for liver cancer and expected direction of covariate

**Covariate**	**Sex**	**Age**	**Direction**
Education (years per capita)	Male, Female	5-80+ years	-1
Health System Access 2 (unitless)			-1
LDI (I$ per capita)			-1
Sociodemographic Index			0
Alcohol (liters per capita)			1
Animal Fats (kcal per capita)			1
Cumulative Cigarettes (15 Years)			1
Cumulative Cigarettes (20 Years)			1
Diabetes Age-Standardized Prevalence (proportion)			1
Hepatitis B Prevalence (proportion)			1
Hepatitis C Prevalence (proportion)			1
Log-transformed SEV scalar: Liver Cancer			1
Mean BMI			1
Red Meat (kcal per capita)			1
Tobacco (cigarettes per capita)			1

©2017 Global Burden of Disease Liver Cancer Collaboration. JAMA Oncology.

eTable 4: Results for CODEm model testing

				Predictive validity					
**Cause**	**Sex**	**Age Start**	**Age End**	**RMSE In**	**RMSE out**	**Trend In**	**Trend Out**	**Coverage In**	**Coverage Out**
Liver cancer [Global]	Male	5-9 years	80+ years	0.290082	0.476742	0.225369	0.221722	0.996852	0.976022
Liver cancer [Data-Rich]	Male	5-9 years	80+ years	0.258174	0.315295	0.211928	0.23142	0.9955	0.994049
Liver cancer [Global]	Female	5-9 years	80+ years	0.31063	0.451614	0.236595	0.228335	0.997464	0.982088
Liver cancer [Data-Rich]	Female	5-9 years	80+ years	0.263012	0.319542	0.218949	0.24845	0.995481	0.993135
RMSE: root-mean-square error<br>Countries include in the data-rich models are shown in eTable 4									

eTable 5: References for etiology splits by country

**Country**	**Years included in estimation (start, end)**		**Citation**	**Liver cancer etiology**
Argentina	2000	2005	Fassio E, Míguez C, Soria S, Palazzo F, Gadano A, Adrover R, Landeira G, Fernández N, García D, Barbero R, Perelstein G, Ríos B, Isla R, Civetta E, Pérez Ravier R, Barzola S, Curciarello J, Colombato LA, Jmeniltzky A. Etiology of hepatocellular carcinoma in Argentina: results of a multicenter retrospective study. Acta Gastroenterol Latinoam. 2009; 39(1): 47-52.	Liver cancer due to alcohol use
Argentina	2000	2005	Fassio E, Míguez C, Soria S, Palazzo F, Gadano A, Adrover R, Landeira G, Fernández N, García D, Barbero R, Perelstein G, Ríos B, Isla R, Civetta E, Pérez Ravier R, Barzola S, Curciarello J, Colombato LA, Jmeniltzky A. Etiology of hepatocellular carcinoma in Argentina: results of a multicenter retrospective study. Acta Gastroenterol Latinoam. 2009; 39(1): 47-52.	Liver cancer due to hepatitis B
Argentina	2000	2005	Fassio E, Míguez C, Soria S, Palazzo F, Gadano A, Adrover R, Landeira G, Fernández N, García D, Barbero R, Perelstein G, Ríos B, Isla R, Civetta E, Pérez Ravier R, Barzola S, Curciarello J, Colombato LA, Jmeniltzky A. Etiology of hepatocellular carcinoma in Argentina: results of a multicenter retrospective study. Acta Gastroenterol Latinoam. 2009; 39(1): 47-52.	Liver cancer due to hepatitis C

©2017 Global Burden of Disease Liver Cancer Collaboration. JAMA Oncology.

Country	Years included in estimation (start, end)		Citation	Liver cancer etiology
Australia	1975	1983	Roberts SK, Kemp W. Hepatocellular carcinoma in an Australian tertiary referral hospital 1975-2002: change in epidemiology and clinical presentation. J Gastroenterol Hepatol. 2007; 22(2): 191-6.	Liver cancer due to alcohol use
Australia	1975	1983	Roberts SK, Kemp W. Hepatocellular carcinoma in an Australian tertiary referral hospital 1975-2002: change in epidemiology and clinical presentation. J Gastroenterol Hepatol. 2007; 22(2): 191-6.	Liver cancer due to hepatitis B
Australia	1975	1983	Roberts SK, Kemp W. Hepatocellular carcinoma in an Australian tertiary referral hospital 1975-2002: change in epidemiology and clinical presentation. J Gastroenterol Hepatol. 2007; 22(2): 191-6.	Liver cancer due to hepatitis C
Australia	1975	1983	Roberts SK, Kemp W. Hepatocellular carcinoma in an Australian tertiary referral hospital 1975-2002: change in epidemiology and clinical presentation. J Gastroenterol Hepatol. 2007; 22(2): 191-6.	Liver cancer due to other causes
Australia	1995	2002	Roberts SK, Kemp W. Hepatocellular carcinoma in an Australian tertiary referral hospital 1975-2002: change in epidemiology and clinical presentation. J Gastroenterol Hepatol. 2007; 22(2): 191-6.	Liver cancer due to alcohol use
Australia	1995	2002	Roberts SK, Kemp W. Hepatocellular carcinoma in an Australian tertiary referral hospital 1975-2002: change in epidemiology and clinical presentation. J Gastroenterol Hepatol. 2007; 22(2): 191-6.	Liver cancer due to hepatitis B
Australia	1995	2002	Roberts SK, Kemp W. Hepatocellular carcinoma in an Australian tertiary referral hospital 1975-2002: change in epidemiology and clinical presentation. J Gastroenterol Hepatol. 2007; 22(2): 191-6.	Liver cancer due to hepatitis C
Australia	1995	2002	Roberts SK, Kemp W. Hepatocellular carcinoma in an Australian tertiary referral hospital 1975-2002: change in epidemiology and clinical presentation. J Gastroenterol Hepatol. 2007; 22(2): 191-6.	Liver cancer due to other causes
Bangladesh	1973	1992	Zaman S, Khan M, Alam K, Williams R. Primary hepatocellular carcinoma and viral hepatitis B and C infection in Bangladeshi subjects. J Trop Med Hyg. 1995; 98(1): 64-8.	Liver cancer due to hepatitis B
Bangladesh	1973	1992	Zaman S, Khan M, Alam K, Williams R. Primary hepatocellular carcinoma and viral hepatitis B and C infection in Bangladeshi subjects. J Trop Med Hyg. 1995; 98(1): 64-8.	Liver cancer due to hepatitis C
Brazil	2004	2009	Carrilho FJ, Kikuchi L, Branco F, Goncalves CS, Mattos AA de, Brazilian HCC Study Group. Clinical	Liver cancer

©2017 Global Burden of Disease Liver Cancer Collaboration. JAMA Oncology.

Country	Years included in estimation (start, end)		Citation	Liver cancer etiology
			and epidemiological aspects of hepatocellular carcinoma in Brazil. Clinics (Sao Paulo). 2010; 65(12): 1285-90.	due to alcohol use
Brazil	2004	2009	Carrilho FJ, Kikuchi L, Branco F, Goncalves CS, Mattos AA de, Brazilian HCC Study Group. Clinical and epidemiological aspects of hepatocellular carcinoma in Brazil. Clinics (Sao Paulo). 2010; 65(12): 1285-90.	Liver cancer due to hepatitis B
Brazil	2004	2009	Carrilho FJ, Kikuchi L, Branco F, Goncalves CS, Mattos AA de, Brazilian HCC Study Group. Clinical and epidemiological aspects of hepatocellular carcinoma in Brazil. Clinics (Sao Paulo). 2010; 65(12): 1285-90.	Liver cancer due to hepatitis C
Brazil	2004	2009	Carrilho FJ, Kikuchi L, Branco F, Goncalves CS, Mattos AA de, Brazilian HCC Study Group. Clinical and epidemiological aspects of hepatocellular carcinoma in Brazil. Clinics (Sao Paulo). 2010; 65(12): 1285-90.	Liver cancer due to other causes
Brazil	1992	1994	Gonçalves CS, Pereira FE, Gayotto LC. Hepatocellular carcinoma in Brazil: report of a national survey (Florianópolis, SC, 1995). Rev Inst Med Trop Sao Paulo. 1997; 39(3): 165-70.	Liver cancer due to alcohol use
Brazil	1992	1994	Gonçalves CS, Pereira FE, Gayotto LC. Hepatocellular carcinoma in Brazil: report of a national survey (Florianópolis, SC, 1995). Rev Inst Med Trop Sao Paulo. 1997; 39(3): 165-70.	Liver cancer due to hepatitis B
Brazil	1992	1994	Gonçalves CS, Pereira FE, Gayotto LC. Hepatocellular carcinoma in Brazil: report of a national survey (Florianópolis, SC, 1995). Rev Inst Med Trop Sao Paulo. 1997; 39(3): 165-70.	Liver cancer due to hepatitis C
Brazil	1992	1994	Gonçalves CS, Pereira FE, Gayotto LC. Hepatocellular carcinoma in Brazil: report of a national survey (Florianópolis, SC, 1995). Rev Inst Med Trop Sao Paulo. 1997; 39(3): 165-70.	Liver cancer due to other causes
Brazil	1992	1999	Miranda ECBM, Moia L de JP, Amaral I do SA, Barbosa MS de B, Conde SRS da S, de Araújo MTF, da Cruz E do RM, Demachki S, Bensabath G, Soares M do CP. [Hepatitis B and C virus infection and the hepatocellular carcinoma in the East Amazon, Brazil]. Rev Soc Bras Med Trop. 2004; 37(Supp 2): 47-51.	Liver cancer due to hepatitis B
Brazil	1992	1999	Miranda ECBM, Moia L de JP, Amaral I do SA, Barbosa MS de B, Conde SRS da S, de Araújo MTF, da Cruz E do RM, Demachki S, Bensabath G, Soares M do CP. [Hepatitis B and C virus infection and the hepatocellular carcinoma in the East Amazon, Brazil]. Rev Soc Bras Med Trop. 2004; 37(Supp 2): 47-51.	Liver cancer due to hepatitis C

©2017 Global Burden of Disease Liver Cancer Collaboration. JAMA Oncology.

Country	Years included in estimation (start, end)		Citation	Liver cancer etiology
Brazil	1998	2010	Osório FMF, Lauar GM, Lima AS, Vidigal PVT, Ferrari TCA, Couto CA. Epidemiological aspects of hepatocellular carcinoma in a referral center of Minas Gerais, Brazil. Arq Gastroenterol. 2013; 50(2): 97-100.	Liver cancer due to alcohol use
Brazil	1998	2010	Osório FMF, Lauar GM, Lima AS, Vidigal PVT, Ferrari TCA, Couto CA. Epidemiological aspects of hepatocellular carcinoma in a referral center of Minas Gerais, Brazil. Arq Gastroenterol. 2013; 50(2): 97-100.	Liver cancer due to hepatitis B
Brazil	1998	2010	Osório FMF, Lauar GM, Lima AS, Vidigal PVT, Ferrari TCA, Couto CA. Epidemiological aspects of hepatocellular carcinoma in a referral center of Minas Gerais, Brazil. Arq Gastroenterol. 2013; 50(2): 97-100.	Liver cancer due to hepatitis C
Brazil	1998	2010	Osório FMF, Lauar GM, Lima AS, Vidigal PVT, Ferrari TCA, Couto CA. Epidemiological aspects of hepatocellular carcinoma in a referral center of Minas Gerais, Brazil. Arq Gastroenterol. 2013; 50(2): 97-100.	Liver cancer due to other causes
China	1998	2000	Gao JD, Shao YF, Xu Y, Ming LH, Wu ZY, Liu GT, Wang XH, Gao WH, Sun YT, Feng XL, Liang LM, Zhang YH, Sun ZT. Tight association of hepatocellular carcinoma with HBV infection in North China. Hepatobiliary Pancreat Dis Int. 2005; 4(1): 46-9.	Liver cancer due to hepatitis B
China	1998	2000	Gao JD, Shao YF, Xu Y, Ming LH, Wu ZY, Liu GT, Wang XH, Gao WH, Sun YT, Feng XL, Liang LM, Zhang YH, Sun ZT. Tight association of hepatocellular carcinoma with HBV infection in North China. Hepatobiliary Pancreat Dis Int. 2005; 4(1): 46-9.	Liver cancer due to hepatitis C
China	1994	2000	Ming L, Thorgeirsson SS, Gail MH, Lu P, Harris CC, Wang N, Shao Y, Wu Z, Liu G, Wang X, Sun Z. Dominant role of hepatitis B virus and cofactor role of aflatoxin in hepatocarcinogenesis in Qidong, China. Hepatology. 2002; 36(5): 1214-20.	Liver cancer due to hepatitis B
China	1994	2000	Ming L, Thorgeirsson SS, Gail MH, Lu P, Harris CC, Wang N, Shao Y, Wu Z, Liu G, Wang X, Sun Z. Dominant role of hepatitis B virus and cofactor role of aflatoxin in hepatocarcinogenesis in Qidong, China. Hepatology. 2002; 36(5): 1214-20.	Liver cancer due to hepatitis C
China	1991	1993	Shi J, Zhu L, Liu S, Xie W-F. A meta-analysis of case-control studies on the combined effect of hepatitis B and C virus infections in causing hepatocellular carcinoma in China. Br J Cancer. 2005; 92(3): 607-12.	Liver cancer due to hepatitis B
China	1991	1993	Shi J, Zhu L, Liu S, Xie W-F. A meta-analysis of case-control studies on the combined effect of hepatitis B and C virus infections in causing hepatocellular carcinoma in China. Br J Cancer. 2005; 92(3): 607-12.	Liver cancer due to hepatitis C
China	1994	1996	Shi J, Zhu L, Liu S, Xie W-F. A meta-analysis of case-control studies on the combined effect of	Liver cancer

©2017 Global Burden of Disease Liver Cancer Collaboration. JAMA Oncology.

Country	Years included in estimation (start, end)		Citation	Liver cancer etiology
			hepatitis B and C virus infections in causing hepatocellular carcinoma in China. Br J Cancer. 2005; 92(3): 607-12.	due to hepatitis B
China	1994	1996	Shi J, Zhu L, Liu S, Xie W-F. A meta-analysis of case-control studies on the combined effect of hepatitis B and C virus infections in causing hepatocellular carcinoma in China. Br J Cancer. 2005; 92(3): 607-12.	Liver cancer due to hepatitis C
China	2000	2002	Wang B-E, Ma W-M, Sulaiman A, Noer S, Sumoharjo S, Sumarsidi D, Tandon BN, Nakao K, Mishiro S, Miyakawa Y, Akahane Y, Suzuki H. Demographic, clinical, and virological characteristics of hepatocellular carcinoma in Asia: survey of 414 patients from four countries. J Med Virol. 2002; 67(3): 394-400.	Liver cancer due to hepatitis B
China	2000	2002	Wang B-E, Ma W-M, Sulaiman A, Noer S, Sumoharjo S, Sumarsidi D, Tandon BN, Nakao K, Mishiro S, Miyakawa Y, Akahane Y, Suzuki H. Demographic, clinical, and virological characteristics of hepatocellular carcinoma in Asia: survey of 414 patients from four countries. J Med Virol. 2002; 67(3): 394-400.	Liver cancer due to hepatitis C
China	2000	2002	Wang B-E, Ma W-M, Sulaiman A, Noer S, Sumoharjo S, Sumarsidi D, Tandon BN, Nakao K, Mishiro S, Miyakawa Y, Akahane Y, Suzuki H. Demographic, clinical, and virological characteristics of hepatocellular carcinoma in Asia: survey of 414 patients from four countries. J Med Virol. 2002; 67(3): 394-400.	Liver cancer due to other causes
China	1994	1995	Zhang JY, Dai M, Wang X, Lu WQ, Li DS, Zhang MX, Wang KJ, Dai LP, Han SG, Zhou YF, Zhuang H. A case-control study of hepatitis B and C virus infection as risk factors for hepatocellular carcinoma in Henan, China. Int J Epidemiol. 1998; 27(4): 574-8.	Liver cancer due to hepatitis B
China	1994	1995	Zhang JY, Dai M, Wang X, Lu WQ, Li DS, Zhang MX, Wang KJ, Dai LP, Han SG, Zhou YF, Zhuang H. A case-control study of hepatitis B and C virus infection as risk factors for hepatocellular carcinoma in Henan, China. Int J Epidemiol. 1998; 27(4): 574-8.	Liver cancer due to hepatitis C
Colombia	2000	2007	Navas M-C, Suarez I, Carreño A, Uribe D, Rios WA, Cortes-Mancera F, Martel G, Vieco B, Lozano D, Jimenez C, Gouas D, Osorio G, Hoyos S, Restrepo JC, Correa G, Jaramillo S, Lopez R, Bravo LE, Arbelaez MP, Scoazec J-Y, Abedi-Ardekani B, Santella RM, Chemin I, Hainaut P. Hepatitis B and Hepatitis C Infection Biomarkers and TP53 Mutations in Hepatocellular Carcinomas from Colombia. Hepat Res Treat. 2011; 2011: 1-10.	Liver cancer due to hepatitis B
Colombia	2000	2007	Navas M-C, Suarez I, Carreño A, Uribe D, Rios WA, Cortes-Mancera F, Martel G, Vieco B, Lozano D, Jimenez C, Gouas D, Osorio G, Hoyos S, Restrepo JC, Correa G, Jaramillo S, Lopez R, Bravo LE, Arbelaez MP, Scoazec J-Y, Abedi-Ardekani B, Santella RM, Chemin I, Hainaut P. Hepatitis B and	Liver cancer due to hepatitis C

©2017 Global Burden of Disease Liver Cancer Collaboration. JAMA Oncology.

Country	Years included in estimation (start, end)		Citation	Liver cancer etiology
			Hepatitis C Infection Biomarkers and TP53 Mutations in Hepatocellular Carcinomas from Colombia. Hepat Res Treat. 2011; 2011: 1-10.	
Croatia	2006	2009	Constantin CV, Streba CT, Rogoveanu I, Nita-Stefanescu L, Ionescu AG. Cirrhosis and Chronic Viral Hepatitis as Risk Factors for Hepatocellular Carcinoma: Romanian Single-clinic Experience. Maedica (Buchar). 2010; 5(4): 265-70.	Liver cancer due to alcohol use
Croatia	2006	2009	Constantin CV, Streba CT, Rogoveanu I, Nita-Stefanescu L, Ionescu AG. Cirrhosis and Chronic Viral Hepatitis as Risk Factors for Hepatocellular Carcinoma: Romanian Single-clinic Experience. Maedica (Buchar). 2010; 5(4): 265-70.	Liver cancer due to hepatitis B
Croatia	2006	2009	Constantin CV, Streba CT, Rogoveanu I, Nita-Stefanescu L, Ionescu AG. Cirrhosis and Chronic Viral Hepatitis as Risk Factors for Hepatocellular Carcinoma: Romanian Single-clinic Experience. Maedica (Buchar). 2010; 5(4): 265-70.	Liver cancer due to hepatitis C
Egypt	2009	2010	Abou El Azm AR, Yousef M, Mansour N, Awad A, El Dardiry S, Abdel Aziz I. New insights on non-B non-C hepatocellular carcinoma in mid Delta Region, Egypt. J Gastrointest Cancer. 2014; 45(3): 276-83.	Liver cancer due to hepatitis B
Egypt	2009	2010	Abou El Azm AR, Yousef M, Mansour N, Awad A, El Dardiry S, Abdel Aziz I. New insights on non-B non-C hepatocellular carcinoma in mid Delta Region, Egypt. J Gastrointest Cancer. 2014; 45(3): 276-83.	Liver cancer due to hepatitis C
Egypt	2009	2010	Abou El Azm AR, Yousef M, Mansour N, Awad A, El Dardiry S, Abdel Aziz I. New insights on non-B non-C hepatocellular carcinoma in mid Delta Region, Egypt. J Gastrointest Cancer. 2014; 45(3): 276-83.	Liver cancer due to other causes
Egypt	2010	2011	Abou El Azm AR, Yousef M, Mansour N, Awad A, El Dardiry S, Abdel Aziz I. New insights on non-B non-C hepatocellular carcinoma in mid Delta Region, Egypt. J Gastrointest Cancer. 2014; 45(3): 276-83.	Liver cancer due to hepatitis B
Egypt	2010	2011	Abou El Azm AR, Yousef M, Mansour N, Awad A, El Dardiry S, Abdel Aziz I. New insights on non-B non-C hepatocellular carcinoma in mid Delta Region, Egypt. J Gastrointest Cancer. 2014; 45(3): 276-83.	Liver cancer due to hepatitis C
Egypt	2010	2011	Abou El Azm AR, Yousef M, Mansour N, Awad A, El Dardiry S, Abdel Aziz I. New insights on non-B non-C hepatocellular carcinoma in mid Delta Region, Egypt. J Gastrointest Cancer. 2014; 45(3): 276-83.	Liver cancer due to other causes
Egypt	2011	2012	Abou El Azm AR, Yousef M, Mansour N, Awad A, El Dardiry S, Abdel Aziz I. New insights on non-B non-C hepatocellular carcinoma in mid Delta Region, Egypt. J Gastrointest Cancer. 2014;	Liver cancer due to

©2017 Global Burden of Disease Liver Cancer Collaboration. JAMA Oncology.

Country	Years included in estimation (start, end)		Citation	Liver cancer etiology
			45(3): 276-83.	hepatitis B
Egypt	2011	2012	Abou El Azm AR, Yousef M, Mansour N, Awad A, El Dardiry S, Abdel Aziz I. New insights on non-B non-C hepatocellular carcinoma in mid Delta Region, Egypt. J Gastrointest Cancer. 2014; 45(3): 276-83.	Liver cancer due to hepatitis C
Egypt	2011	2012	Abou El Azm AR, Yousef M, Mansour N, Awad A, El Dardiry S, Abdel Aziz I. New insights on non-B non-C hepatocellular carcinoma in mid Delta Region, Egypt. J Gastrointest Cancer. 2014; 45(3): 276-83.	Liver cancer due to other causes
Egypt	1993	1997	el-Zayadi A-R, Badran HM, Barakat EMF, Attia M el-D, Shawky S, Mohamed MK, Selim O, Saeid A. Hepatocellular carcinoma in Egypt: a single center study over a decade. World J Gastroenterol. 2005; 11(33): 5193-8.	Liver cancer due to hepatitis B
Egypt	1993	1997	el-Zayadi A-R, Badran HM, Barakat EMF, Attia M el-D, Shawky S, Mohamed MK, Selim O, Saeid A. Hepatocellular carcinoma in Egypt: a single center study over a decade. World J Gastroenterol. 2005; 11(33): 5193-8.	Liver cancer due to hepatitis C
Egypt	1998	2002	el-Zayadi A-R, Badran HM, Barakat EMF, Attia M el-D, Shawky S, Mohamed MK, Selim O, Saeid A. Hepatocellular carcinoma in Egypt: a single center study over a decade. World J Gastroenterol. 2005; 11(33): 5193-8.	Liver cancer due to hepatitis B
Egypt	1998	2002	el-Zayadi A-R, Badran HM, Barakat EMF, Attia M el-D, Shawky S, Mohamed MK, Selim O, Saeid A. Hepatocellular carcinoma in Egypt: a single center study over a decade. World J Gastroenterol. 2005; 11(33): 5193-8.	Liver cancer due to hepatitis C
Egypt	1995	1996	Hassan MM, Zaghloul AS, El-Serag HB, Soliman O, Patt YZ, Chappell CL, Beasley RP, Hwang LY. The role of hepatitis C in hepatocellular carcinoma: a case control study among Egyptian patients. J Clin Gastroenterol. 2001; 33(2): 123-6.	Liver cancer due to hepatitis B
Egypt	1995	1996	Hassan MM, Zaghloul AS, El-Serag HB, Soliman O, Patt YZ, Chappell CL, Beasley RP, Hwang LY. The role of hepatitis C in hepatocellular carcinoma: a case control study among Egyptian patients. J Clin Gastroenterol. 2001; 33(2): 123-6.	Liver cancer due to hepatitis C
Egypt	1995	1996	Mabrouk GM. Prevalence of hepatitis C infection and schistosomiasis in Egyptian patients with hepatocellular carcinoma. Dis Markers. 1997; 13(3): 177-82.	Liver cancer due to hepatitis B
Egypt	1995	1996	Mabrouk GM. Prevalence of hepatitis C infection and schistosomiasis in Egyptian patients with hepatocellular carcinoma. Dis Markers. 1997; 13(3): 177-82.	Liver cancer due to hepatitis C

©2017 Global Burden of Disease Liver Cancer Collaboration. JAMA Oncology.

Country	Years included in estimation (start, end)		Citation	Liver cancer etiology
Gabon	1990	1998	Perret J-L, Moussavou-Kombila J-B, Delaporte E, Pemba L-F, Boguikouma J-B, Matton T, Larouze B. [HBs Ag and antibodies to hepatitis C virus in complicated chronic liver disease in Gabon. A case control study]. Gastroenterol Clin Biol. 2002; 26(2): 131-5.	Liver cancer due to hepatitis B
Gabon	1990	1998	Perret J-L, Moussavou-Kombila J-B, Delaporte E, Pemba L-F, Boguikouma J-B, Matton T, Larouze B. [HBs Ag and antibodies to hepatitis C virus in complicated chronic liver disease in Gabon. A case control study]. Gastroenterol Clin Biol. 2002; 26(2): 131-5.	Liver cancer due to hepatitis C
Germany	2007	2008	Ertle J, Dechêne A, Sowa J-P, Penndorf V, Herzer K, Kaiser G, Schlaak JF, Gerken G, Syn W-K, Canbay A. Non-alcoholic fatty liver disease progresses to hepatocellular carcinoma in the absence of apparent cirrhosis. Int J Cancer. 2011; 128(10): 2436-43.	Liver cancer due to alcohol use
Germany	2007	2008	Ertle J, Dechêne A, Sowa J-P, Penndorf V, Herzer K, Kaiser G, Schlaak JF, Gerken G, Syn W-K, Canbay A. Non-alcoholic fatty liver disease progresses to hepatocellular carcinoma in the absence of apparent cirrhosis. Int J Cancer. 2011; 128(10): 2436-43.	Liver cancer due to hepatitis B
Germany	2007	2008	Ertle J, Dechêne A, Sowa J-P, Penndorf V, Herzer K, Kaiser G, Schlaak JF, Gerken G, Syn W-K, Canbay A. Non-alcoholic fatty liver disease progresses to hepatocellular carcinoma in the absence of apparent cirrhosis. Int J Cancer. 2011; 128(10): 2436-43.	Liver cancer due to hepatitis C
Germany	2007	2008	Ertle J, Dechêne A, Sowa J-P, Penndorf V, Herzer K, Kaiser G, Schlaak JF, Gerken G, Syn W-K, Canbay A. Non-alcoholic fatty liver disease progresses to hepatocellular carcinoma in the absence of apparent cirrhosis. Int J Cancer. 2011; 128(10): 2436-43.	Liver cancer due to other causes
Germany	1994	2000	Hellerbrand C, Hartmann A, Richter G, Knöll A, Wiest R, Schölmerich J, Lock G. Hepatocellular carcinoma in southern Germany: epidemiological and clinicopathological characteristics and risk factors. Dig Dis. 2001; 19(4): 345-51.	Liver cancer due to alcohol use
Germany	1994	2000	Hellerbrand C, Hartmann A, Richter G, Knöll A, Wiest R, Schölmerich J, Lock G. Hepatocellular carcinoma in southern Germany: epidemiological and clinicopathological characteristics and risk factors. Dig Dis. 2001; 19(4): 345-51.	Liver cancer due to hepatitis B
Germany	1994	2000	Hellerbrand C, Hartmann A, Richter G, Knöll A, Wiest R, Schölmerich J, Lock G. Hepatocellular carcinoma in southern Germany: epidemiological and clinicopathological characteristics and risk factors. Dig Dis. 2001; 19(4): 345-51.	Liver cancer due to hepatitis C
Germany	1994	2000	Hellerbrand C, Hartmann A, Richter G, Knöll A, Wiest R, Schölmerich J, Lock G. Hepatocellular carcinoma in southern Germany: epidemiological and clinicopathological characteristics and risk factors. Dig Dis. 2001; 19(4): 345-51.	Liver cancer due to other causes
Germany	1994	2008	Kirchner G, Kirovski G, Hebestreit A, Schölmerich J, Schlitt HJ, Stoeltzing O, Hellerbrand C.	Liver cancer

©2017 Global Burden of Disease Liver Cancer Collaboration. JAMA Oncology.

Country	Years included in estimation (start, end)		Citation	Liver cancer etiology
			Epidemiology and survival of patients with hepatocellular carcinoma in Southern Germany. Int J Clin Exp Med. 2010; 3(2): 169-79.	due to alcohol use
Germany	1994	2008	Kirchner G, Kirovski G, Hebestreit A, Schölmerich J, Schlitt HJ, Stoeltzing O, Hellerbrand C. Epidemiology and survival of patients with hepatocellular carcinoma in Southern Germany. Int J Clin Exp Med. 2010; 3(2): 169-79.	Liver cancer due to hepatitis B
Germany	1994	2008	Kirchner G, Kirovski G, Hebestreit A, Schölmerich J, Schlitt HJ, Stoeltzing O, Hellerbrand C. Epidemiology and survival of patients with hepatocellular carcinoma in Southern Germany. Int J Clin Exp Med. 2010; 3(2): 169-79.	Liver cancer due to hepatitis C
Germany	1994	2008	Kirchner G, Kirovski G, Hebestreit A, Schölmerich J, Schlitt HJ, Stoeltzing O, Hellerbrand C. Epidemiology and survival of patients with hepatocellular carcinoma in Southern Germany. Int J Clin Exp Med. 2010; 3(2): 169-79.	Liver cancer due to other causes
Germany	1993	1997	Kubicka S, Rudolph KL, Hanke M, Tietze MK, Tillmann HL, Trautwein C, Manns M. Hepatocellular carcinoma in Germany: a retrospective epidemiological study from a low-endemic area. Liver. 2000; 20(4): 312-8.	Liver cancer due to alcohol use
Germany	1993	1997	Kubicka S, Rudolph KL, Hanke M, Tietze MK, Tillmann HL, Trautwein C, Manns M. Hepatocellular carcinoma in Germany: a retrospective epidemiological study from a low-endemic area. Liver. 2000; 20(4): 312-8.	Liver cancer due to hepatitis B
Germany	1993	1997	Kubicka S, Rudolph KL, Hanke M, Tietze MK, Tillmann HL, Trautwein C, Manns M. Hepatocellular carcinoma in Germany: a retrospective epidemiological study from a low-endemic area. Liver. 2000; 20(4): 312-8.	Liver cancer due to hepatitis C
Germany	1993	1997	Kubicka S, Rudolph KL, Hanke M, Tietze MK, Tillmann HL, Trautwein C, Manns M. Hepatocellular carcinoma in Germany: a retrospective epidemiological study from a low-endemic area. Liver. 2000; 20(4): 312-8.	Liver cancer due to other causes
Germany	1998	2009	op den Winkel M, Nagel D, Sappl J, op den Winkel P, Lamerz R, Zech CJ, Straub G, Nickel T, Rentsch M, Stieber P, Göke B, Kolligs FT. Prognosis of patients with hepatocellular carcinoma. Validation and ranking of established staging-systems in a large western HCC-cohort. PLoS One. 2012; 7(10): e45066.	Liver cancer due to alcohol use
Germany	1998	2009	op den Winkel M, Nagel D, Sappl J, op den Winkel P, Lamerz R, Zech CJ, Straub G, Nickel T, Rentsch M, Stieber P, Göke B, Kolligs FT. Prognosis of patients with hepatocellular carcinoma. Validation and ranking of established staging-systems in a large western HCC-cohort. PLoS One. 2012; 7(10): e45066.	Liver cancer due to hepatitis B

©2017 Global Burden of Disease Liver Cancer Collaboration. JAMA Oncology.

Country	Years included in estimation (start, end)		Citation	Liver cancer etiology
Germany	1998	2009	op den Winkel M, Nagel D, Sappl J, op den Winkel P, Lamerz R, Zech CJ, Straub G, Nickel T, Rentsch M, Stieber P, Göke B, Kolligs FT. Prognosis of patients with hepatocellular carcinoma. Validation and ranking of established staging-systems in a large western HCC-cohort. PLoS One. 2012; 7(10): e45066.	Liver cancer due to hepatitis C
Germany	1998	2009	op den Winkel M, Nagel D, Sappl J, op den Winkel P, Lamerz R, Zech CJ, Straub G, Nickel T, Rentsch M, Stieber P, Göke B, Kolligs FT. Prognosis of patients with hepatocellular carcinoma. Validation and ranking of established staging-systems in a large western HCC-cohort. PLoS One. 2012; 7(10): e45066.	Liver cancer due to other causes
Germany	1990	1997	Petry W, Heintges T, Hensel F, Erhardt A, Wenning M, Niederau C, Häussinger D. [Hepatocellular carcinoma in Germany. Epidemiology, etiology, clinical aspects and prognosis in 100 consecutive patients of a university clinic]. Z Gastroenterol. 1997; 35(12): 1059-67.	Liver cancer due to alcohol use
Germany	1990	1997	Petry W, Heintges T, Hensel F, Erhardt A, Wenning M, Niederau C, Häussinger D. [Hepatocellular carcinoma in Germany. Epidemiology, etiology, clinical aspects and prognosis in 100 consecutive patients of a university clinic]. Z Gastroenterol. 1997; 35(12): 1059-67.	Liver cancer due to hepatitis B
Germany	1990	1997	Petry W, Heintges T, Hensel F, Erhardt A, Wenning M, Niederau C, Häussinger D. [Hepatocellular carcinoma in Germany. Epidemiology, etiology, clinical aspects and prognosis in 100 consecutive patients of a university clinic]. Z Gastroenterol. 1997; 35(12): 1059-67.	Liver cancer due to hepatitis C
Germany	1990	1997	Petry W, Heintges T, Hensel F, Erhardt A, Wenning M, Niederau C, Häussinger D. [Hepatocellular carcinoma in Germany. Epidemiology, etiology, clinical aspects and prognosis in 100 consecutive patients of a university clinic]. Z Gastroenterol. 1997; 35(12): 1059-67.	Liver cancer due to other causes
Germany	1997	1999	Rabe C, Pilz T, Klostermann C, Berna M, Schild HH, Sauerbruch T, Caselmann WH. Clinical characteristics and outcome of a cohort of 101 patients with hepatocellular carcinoma. World J Gastroenterol. 2001; 7(2): 208-15.	Liver cancer due to alcohol use
Germany	1997	1999	Rabe C, Pilz T, Klostermann C, Berna M, Schild HH, Sauerbruch T, Caselmann WH. Clinical characteristics and outcome of a cohort of 101 patients with hepatocellular carcinoma. World J Gastroenterol. 2001; 7(2): 208-15.	Liver cancer due to hepatitis B
Germany	1997	1999	Rabe C, Pilz T, Klostermann C, Berna M, Schild HH, Sauerbruch T, Caselmann WH. Clinical characteristics and outcome of a cohort of 101 patients with hepatocellular carcinoma. World J Gastroenterol. 2001; 7(2): 208-15.	Liver cancer due to hepatitis C
Germany	1997	1999	Rabe C, Pilz T, Klostermann C, Berna M, Schild HH, Sauerbruch T, Caselmann WH. Clinical characteristics and outcome of a cohort of 101 patients with hepatocellular carcinoma. World J	Liver cancer due to other

©2017 Global Burden of Disease Liver Cancer Collaboration. JAMA Oncology.

Country	Years included in estimation (start, end)		Citation	Liver cancer etiology
			Gastroenterol. 2001; 7(2): 208-15.	causes
Germany	1994	1998	Schütte K, Kipper M, Kahl S, Bornschein J, Götze T, Adolf D, Arend J, Seidensticker R, Lippert H, Ricke J, Malfertheiner P. Clinical characteristics and time trends in etiology of hepatocellular cancer in Germany. Digestion. 2013; 87(3): 147-59.	Liver cancer due to alcohol use
Germany	1994	1998	Schütte K, Kipper M, Kahl S, Bornschein J, Götze T, Adolf D, Arend J, Seidensticker R, Lippert H, Ricke J, Malfertheiner P. Clinical characteristics and time trends in etiology of hepatocellular cancer in Germany. Digestion. 2013; 87(3): 147-59.	Liver cancer due to hepatitis B
Germany	1994	1998	Schütte K, Kipper M, Kahl S, Bornschein J, Götze T, Adolf D, Arend J, Seidensticker R, Lippert H, Ricke J, Malfertheiner P. Clinical characteristics and time trends in etiology of hepatocellular cancer in Germany. Digestion. 2013; 87(3): 147-59.	Liver cancer due to hepatitis C
Germany	1994	1998	Schütte K, Kipper M, Kahl S, Bornschein J, Götze T, Adolf D, Arend J, Seidensticker R, Lippert H, Ricke J, Malfertheiner P. Clinical characteristics and time trends in etiology of hepatocellular cancer in Germany. Digestion. 2013; 87(3): 147-59.	Liver cancer due to other causes
Germany	1999	2003	Schütte K, Kipper M, Kahl S, Bornschein J, Götze T, Adolf D, Arend J, Seidensticker R, Lippert H, Ricke J, Malfertheiner P. Clinical characteristics and time trends in etiology of hepatocellular cancer in Germany. Digestion. 2013; 87(3): 147-59.	Liver cancer due to alcohol use
Germany	1999	2003	Schütte K, Kipper M, Kahl S, Bornschein J, Götze T, Adolf D, Arend J, Seidensticker R, Lippert H, Ricke J, Malfertheiner P. Clinical characteristics and time trends in etiology of hepatocellular cancer in Germany. Digestion. 2013; 87(3): 147-59.	Liver cancer due to hepatitis B
Germany	1999	2003	Schütte K, Kipper M, Kahl S, Bornschein J, Götze T, Adolf D, Arend J, Seidensticker R, Lippert H, Ricke J, Malfertheiner P. Clinical characteristics and time trends in etiology of hepatocellular cancer in Germany. Digestion. 2013; 87(3): 147-59.	Liver cancer due to hepatitis C
Germany	1999	2003	Schütte K, Kipper M, Kahl S, Bornschein J, Götze T, Adolf D, Arend J, Seidensticker R, Lippert H, Ricke J, Malfertheiner P. Clinical characteristics and time trends in etiology of hepatocellular cancer in Germany. Digestion. 2013; 87(3): 147-59.	Liver cancer due to other causes
Germany	2004	2008	Schütte K, Kipper M, Kahl S, Bornschein J, Götze T, Adolf D, Arend J, Seidensticker R, Lippert H, Ricke J, Malfertheiner P. Clinical characteristics and time trends in etiology of hepatocellular cancer in Germany. Digestion. 2013; 87(3): 147-59.	Liver cancer due to alcohol use
Germany	2004	2008	Schütte K, Kipper M, Kahl S, Bornschein J, Götze T, Adolf D, Arend J, Seidensticker R, Lippert H, Ricke J, Malfertheiner P. Clinical characteristics and time trends in etiology of hepatocellular cancer in Germany. Digestion. 2013; 87(3): 147-59.	Liver cancer due to hepatitis B

©2017 Global Burden of Disease Liver Cancer Collaboration. JAMA Oncology.

Country	Years included in estimation (start, end)		Citation	Liver cancer etiology
Germany	2004	2008	Schütte K, Kipper M, Kahl S, Bornschein J, Götze T, Adolf D, Arend J, Seidensticker R, Lippert H, Ricke J, Malfertheiner P. Clinical characteristics and time trends in etiology of hepatocellular cancer in Germany. Digestion. 2013; 87(3): 147-59.	Liver cancer due to hepatitis C
Germany	2004	2008	Schütte K, Kipper M, Kahl S, Bornschein J, Götze T, Adolf D, Arend J, Seidensticker R, Lippert H, Ricke J, Malfertheiner P. Clinical characteristics and time trends in etiology of hepatocellular cancer in Germany. Digestion. 2013; 87(3): 147-59.	Liver cancer due to other causes
Germany	2009	2011	Schütte K, Kipper M, Kahl S, Bornschein J, Götze T, Adolf D, Arend J, Seidensticker R, Lippert H, Ricke J, Malfertheiner P. Clinical characteristics and time trends in etiology of hepatocellular cancer in Germany. Digestion. 2013; 87(3): 147-59.	Liver cancer due to alcohol use
Germany	2009	2011	Schütte K, Kipper M, Kahl S, Bornschein J, Götze T, Adolf D, Arend J, Seidensticker R, Lippert H, Ricke J, Malfertheiner P. Clinical characteristics and time trends in etiology of hepatocellular cancer in Germany. Digestion. 2013; 87(3): 147-59.	Liver cancer due to hepatitis B
Germany	2009	2011	Schütte K, Kipper M, Kahl S, Bornschein J, Götze T, Adolf D, Arend J, Seidensticker R, Lippert H, Ricke J, Malfertheiner P. Clinical characteristics and time trends in etiology of hepatocellular cancer in Germany. Digestion. 2013; 87(3): 147-59.	Liver cancer due to hepatitis C
Germany	2009	2011	Schütte K, Kipper M, Kahl S, Bornschein J, Götze T, Adolf D, Arend J, Seidensticker R, Lippert H, Ricke J, Malfertheiner P. Clinical characteristics and time trends in etiology of hepatocellular cancer in Germany. Digestion. 2013; 87(3): 147-59.	Liver cancer due to other causes
Greece	1995	1998	Kuper HE, Tzonou A, Kaklamani E, Hadziyannis S, Tasopoulos N, Lagiou P, Trichopoulos D, Stuver S. Hepatitis B and C viruses in the etiology of hepatocellular carcinoma; a study in Greece using third-generation assays. Cancer Causes Control. 2000; 11(2): 171-5.	Liver cancer due to hepatitis B
Greece	1995	1998	Kuper HE, Tzonou A, Kaklamani E, Hadziyannis S, Tasopoulos N, Lagiou P, Trichopoulos D, Stuver S. Hepatitis B and C viruses in the etiology of hepatocellular carcinoma; a study in Greece using third-generation assays. Cancer Causes Control. 2000; 11(2): 171-5.	Liver cancer due to hepatitis C
Greece	1996	2000	Raptis I, Koskinas J, Emmanouil T, Hadziyannis S. Changing relative roles of hepatitis B and C viruses in the aetiology of hepatocellular carcinoma in Greece. Epidemiological and clinical observations. J Viral Hepat. 2003; 10(6): 450-4.	Liver cancer due to hepatitis B
Greece	1996	2000	Raptis I, Koskinas J, Emmanouil T, Hadziyannis S. Changing relative roles of hepatitis B and C viruses in the aetiology of hepatocellular carcinoma in Greece. Epidemiological and clinical observations. J Viral Hepat. 2003; 10(6): 450-4.	Liver cancer due to hepatitis C
India	1991	2003	Kumar R, Saraswat MK, Sharma BC, Sakhuja P, Sarin SK. Characteristics of hepatocellular	Liver cancer

©2017 Global Burden of Disease Liver Cancer Collaboration. JAMA Oncology.

Country	Years included in estimation (start, end)		Citation	Liver cancer etiology
			carcinoma in India: a retrospective analysis of 191 cases. QJM. 2008; 101(6): 479-85.	due to alcohol use
India	1991	2003	Kumar R, Saraswat MK, Sharma BC, Sakhuja P, Sarin SK. Characteristics of hepatocellular carcinoma in India: a retrospective analysis of 191 cases. QJM. 2008; 101(6): 479-85.	Liver cancer due to hepatitis C
India	1991	2003	Kumar R, Saraswat MK, Sharma BC, Sakhuja P, Sarin SK. Characteristics of hepatocellular carcinoma in India: a retrospective analysis of 191 cases. QJM. 2008; 101(6): 479-85.	Liver cancer due to other causes
India	1993	1999	Sarin SK, Thakur V, Guptan RC, Saigal S, Malhotra V, Thyagarajan SP, Das BC. Profile of hepatocellular carcinoma in India: an insight into the possible etiologic associations. J Gastroenterol Hepatol. 2001; 16(6): 666-73.	Liver cancer due to alcohol use
India	1993	1999	Sarin SK, Thakur V, Guptan RC, Saigal S, Malhotra V, Thyagarajan SP, Das BC. Profile of hepatocellular carcinoma in India: an insight into the possible etiologic associations. J Gastroenterol Hepatol. 2001; 16(6): 666-73.	Liver cancer due to hepatitis B
India	1993	1999	Sarin SK, Thakur V, Guptan RC, Saigal S, Malhotra V, Thyagarajan SP, Das BC. Profile of hepatocellular carcinoma in India: an insight into the possible etiologic associations. J Gastroenterol Hepatol. 2001; 16(6): 666-73.	Liver cancer due to hepatitis C
India	1993	1999	Sarin SK, Thakur V, Guptan RC, Saigal S, Malhotra V, Thyagarajan SP, Das BC. Profile of hepatocellular carcinoma in India: an insight into the possible etiologic associations. J Gastroenterol Hepatol. 2001; 16(6): 666-73.	Liver cancer due to other causes
India	2007	2010	Sood A, Midha V, Goyal O, Goyal P, Sood N, Sharma SK. Profile of hepatocellular carcinoma in a tertiary care hospital in Punjab in northern India. Indian J Gastroenterol. 2014; 33(1): 35-40.	Liver cancer due to alcohol use
India	2007	2010	Sood A, Midha V, Goyal O, Goyal P, Sood N, Sharma SK. Profile of hepatocellular carcinoma in a tertiary care hospital in Punjab in northern India. Indian J Gastroenterol. 2014; 33(1): 35-40.	Liver cancer due to hepatitis B
India	2007	2010	Sood A, Midha V, Goyal O, Goyal P, Sood N, Sharma SK. Profile of hepatocellular carcinoma in a tertiary care hospital in Punjab in northern India. Indian J Gastroenterol. 2014; 33(1): 35-40.	Liver cancer due to hepatitis C
India	2007	2010	Sood A, Midha V, Goyal O, Goyal P, Sood N, Sharma SK. Profile of hepatocellular carcinoma in a tertiary care hospital in Punjab in northern India. Indian J Gastroenterol. 2014; 33(1): 35-40.	Liver cancer due to other

©2017 Global Burden of Disease Liver Cancer Collaboration. JAMA Oncology.

Country	Years included in estimation (start, end)		Citation	Liver cancer etiology
				causes
India	2000	2002	Wang B-E, Ma W-M, Sulaiman A, Noer S, Sumoharjo S, Sumarsidi D, Tandon BN, Nakao K, Mishiro S, Miyakawa Y, Akahane Y, Suzuki H. Demographic, clinical, and virological characteristics of hepatocellular carcinoma in Asia: survey of 414 patients from four countries. J Med Virol. 2002; 67(3): 394-400.	Liver cancer due to hepatitis B
India	2000	2002	Wang B-E, Ma W-M, Sulaiman A, Noer S, Sumoharjo S, Sumarsidi D, Tandon BN, Nakao K, Mishiro S, Miyakawa Y, Akahane Y, Suzuki H. Demographic, clinical, and virological characteristics of hepatocellular carcinoma in Asia: survey of 414 patients from four countries. J Med Virol. 2002; 67(3): 394-400.	Liver cancer due to hepatitis C
India	2000	2002	Wang B-E, Ma W-M, Sulaiman A, Noer S, Sumoharjo S, Sumarsidi D, Tandon BN, Nakao K, Mishiro S, Miyakawa Y, Akahane Y, Suzuki H. Demographic, clinical, and virological characteristics of hepatocellular carcinoma in Asia: survey of 414 patients from four countries. J Med Virol. 2002; 67(3): 394-400.	Liver cancer due to other causes
Indonesia	1991	1993	Hadiwandowo S, Tsuda F, Okamoto H, Tokita H, Wang Y, Tanaka T, Miyakawa Y, Mayumi M. Hepatitis B virus subtypes and hepatitis C virus genotypes in patients with chronic liver disease or on maintenance hemodialysis in Indonesia. J Med Virol. 1994; 43(2): 182-6.	Liver cancer due to hepatitis B
Indonesia	1991	1993	Hadiwandowo S, Tsuda F, Okamoto H, Tokita H, Wang Y, Tanaka T, Miyakawa Y, Mayumi M. Hepatitis B virus subtypes and hepatitis C virus genotypes in patients with chronic liver disease or on maintenance hemodialysis in Indonesia. J Med Virol. 1994; 43(2): 182-6.	Liver cancer due to hepatitis C
Indonesia	2000	2002	Wang B-E, Ma W-M, Sulaiman A, Noer S, Sumoharjo S, Sumarsidi D, Tandon BN, Nakao K, Mishiro S, Miyakawa Y, Akahane Y, Suzuki H. Demographic, clinical, and virological characteristics of hepatocellular carcinoma in Asia: survey of 414 patients from four countries. J Med Virol. 2002; 67(3): 394-400.	Liver cancer due to hepatitis B
Indonesia	2000	2002	Wang B-E, Ma W-M, Sulaiman A, Noer S, Sumoharjo S, Sumarsidi D, Tandon BN, Nakao K, Mishiro S, Miyakawa Y, Akahane Y, Suzuki H. Demographic, clinical, and virological characteristics of hepatocellular carcinoma in Asia: survey of 414 patients from four countries. J Med Virol. 2002; 67(3): 394-400.	Liver cancer due to hepatitis C
Iran	1999	2004	Hajiani E, Masjedizadeh R, Hashemi J, Azmi M, Rajabi T. Risk factors for hepatocellular carcinoma in Southern Iran. Saudi Med J. 2005; 26(6): 974-7.	Liver cancer due to alcohol use
Iran	1999	2004	Hajiani E, Masjedizadeh R, Hashemi J, Azmi M, Rajabi T. Risk factors for hepatocellular	Liver cancer

©2017 Global Burden of Disease Liver Cancer Collaboration. JAMA Oncology.

Country	Years included in estimation (start, end)		Citation	Liver cancer etiology
			carcinoma in Southern Iran. Saudi Med J. 2005; 26(6): 974-7.	due to hepatitis B
Iran	1999	2004	Hajiani E, Masjedizadeh R, Hashemi J, Azmi M, Rajabi T. Risk factors for hepatocellular carcinoma in Southern Iran. Saudi Med J. 2005; 26(6): 974-7.	Liver cancer due to hepatitis C
Iran	1999	2004	Hajiani E, Masjedizadeh R, Hashemi J, Azmi M, Rajabi T. Risk factors for hepatocellular carcinoma in Southern Iran. Saudi Med J. 2005; 26(6): 974-7.	Liver cancer due to other causes
Italy	1995	1997	Chiesa R, Donato F, Tagger A, Favret M, Ribero ML, Nardi G, Gelatti U, Bucella E, Tomasi E, Portolani N, Bonetti M, Bettini L, Pelizzari G, Salmi A, Savio A, Garatti M, Callea F. Etiology of hepatocellular carcinoma in Italian patients with and without cirrhosis. Cancer Epidemiol Biomarkers Prev. 2000; 9(2): 213-6.	Liver cancer due to alcohol use
Italy	1995	1997	Chiesa R, Donato F, Tagger A, Favret M, Ribero ML, Nardi G, Gelatti U, Bucella E, Tomasi E, Portolani N, Bonetti M, Bettini L, Pelizzari G, Salmi A, Savio A, Garatti M, Callea F. Etiology of hepatocellular carcinoma in Italian patients with and without cirrhosis. Cancer Epidemiol Biomarkers Prev. 2000; 9(2): 213-6.	Liver cancer due to hepatitis B
Italy	1995	1997	Chiesa R, Donato F, Tagger A, Favret M, Ribero ML, Nardi G, Gelatti U, Bucella E, Tomasi E, Portolani N, Bonetti M, Bettini L, Pelizzari G, Salmi A, Savio A, Garatti M, Callea F. Etiology of hepatocellular carcinoma in Italian patients with and without cirrhosis. Cancer Epidemiol Biomarkers Prev. 2000; 9(2): 213-6.	Liver cancer due to hepatitis C
Italy	1995	1997	Chiesa R, Donato F, Tagger A, Favret M, Ribero ML, Nardi G, Gelatti U, Bucella E, Tomasi E, Portolani N, Bonetti M, Bettini L, Pelizzari G, Salmi A, Savio A, Garatti M, Callea F. Etiology of hepatocellular carcinoma in Italian patients with and without cirrhosis. Cancer Epidemiol Biomarkers Prev. 2000; 9(2): 213-6.	Liver cancer due to other causes
Italy	1995	1996	Donato F, Tagger A, Chiesa R, Ribero ML, Tomasoni V, Fasola M, Gelatti U, Portera G, Boffetta P, Nardi G. Hepatitis B and C virus infection, alcohol drinking, and hepatocellular carcinoma: a case-control study in Italy. Brescia HCC Study. Hepatology. 1997; 26(3): 579-84.	Liver cancer due to hepatitis B
Italy	1995	1996	Donato F, Tagger A, Chiesa R, Ribero ML, Tomasoni V, Fasola M, Gelatti U, Portera G, Boffetta P, Nardi G. Hepatitis B and C virus infection, alcohol drinking, and hepatocellular carcinoma: a case-control study in Italy. Brescia HCC Study. Hepatology. 1997; 26(3): 579-84.	Liver cancer due to hepatitis C
Italy	1999	2002	Franceschi S, Montella M, Polesel J, La Vecchia C, Crispo A, Dal Maso L, Casarin P, Izzo F,	Liver cancer

©2017 Global Burden of Disease Liver Cancer Collaboration. JAMA Oncology.

Country	Years included in estimation (start, end)		Citation	Liver cancer etiology
			Tommasi LG, Chemin I, Trépo C, Crovatto M, Talamini R. Hepatitis viruses, alcohol, and tobacco in the etiology of hepatocellular carcinoma in Italy. Cancer Epidemiol Biomarkers Prev. 2006; 15(4): 683-9.	due to alcohol use
Italy	1999	2002	Franceschi S, Montella M, Polesel J, La Vecchia C, Crispo A, Dal Maso L, Casarin P, Izzo F, Tommasi LG, Chemin I, Trépo C, Crovatto M, Talamini R. Hepatitis viruses, alcohol, and tobacco in the etiology of hepatocellular carcinoma in Italy. Cancer Epidemiol Biomarkers Prev. 2006; 15(4): 683-9.	Liver cancer due to hepatitis B
Italy	1999	2002	Franceschi S, Montella M, Polesel J, La Vecchia C, Crispo A, Dal Maso L, Casarin P, Izzo F, Tommasi LG, Chemin I, Trépo C, Crovatto M, Talamini R. Hepatitis viruses, alcohol, and tobacco in the etiology of hepatocellular carcinoma in Italy. Cancer Epidemiol Biomarkers Prev. 2006; 15(4): 683-9.	Liver cancer due to hepatitis C
Italy	1999	2002	Franceschi S, Montella M, Polesel J, La Vecchia C, Crispo A, Dal Maso L, Casarin P, Izzo F, Tommasi LG, Chemin I, Trépo C, Crovatto M, Talamini R. Hepatitis viruses, alcohol, and tobacco in the etiology of hepatocellular carcinoma in Italy. Cancer Epidemiol Biomarkers Prev. 2006; 15(4): 683-9.	Liver cancer due to hepatitis C
Italy	1999	2002	Franceschi S, Montella M, Polesel J, La Vecchia C, Crispo A, Dal Maso L, Casarin P, Izzo F, Tommasi LG, Chemin I, Trépo C, Crovatto M, Talamini R. Hepatitis viruses, alcohol, and tobacco in the etiology of hepatocellular carcinoma in Italy. Cancer Epidemiol Biomarkers Prev. 2006; 15(4): 683-9.	Liver cancer due to other causes
Italy	1995	1998	Gelatti U, Donato F, Tagger A, Fantoni C, Portolani N, Ribero ML, Martelli C, Trevisi P, Covolo L, Simonati C, Nardi G, Brescia HCC Study. Etiology of hepatocellular carcinoma influences clinical and pathologic features but not patient survival. Am J Gastroenterol. 2003; 98(4): 907-14.	Liver cancer due to alcohol use
Italy	1995	1998	Gelatti U, Donato F, Tagger A, Fantoni C, Portolani N, Ribero ML, Martelli C, Trevisi P, Covolo L, Simonati C, Nardi G, Brescia HCC Study. Etiology of hepatocellular carcinoma influences clinical and pathologic features but not patient survival. Am J Gastroenterol. 2003; 98(4): 907-14.	Liver cancer due to hepatitis B
Italy	1995	1998	Gelatti U, Donato F, Tagger A, Fantoni C, Portolani N, Ribero ML, Martelli C, Trevisi P, Covolo L, Simonati C, Nardi G, Brescia HCC Study. Etiology of hepatocellular carcinoma influences clinical and pathologic features but not patient survival. Am J Gastroenterol. 2003; 98(4): 907-14.	Liver cancer due to hepatitis C
Italy	1995	1998	Gelatti U, Donato F, Tagger A, Fantoni C, Portolani N, Ribero ML, Martelli C, Trevisi P, Covolo L, Simonati C, Nardi G, Brescia HCC Study. Etiology of hepatocellular carcinoma influences clinical and pathologic features but not patient survival. Am J Gastroenterol. 2003; 98(4): 907-14.	Liver cancer due to other causes

©2017 Global Burden of Disease Liver Cancer Collaboration. JAMA Oncology.

Country	Years included in estimation (start, end)		Citation	Liver cancer etiology
Italy	2001	2001	Sagnelli E, Stroffolini T, Mele A, Almasio P, Coppola N, Ferrigno L, Scolastico C, Onofrio M, Imparato M, Filippini P. The importance of HCV on the burden of chronic liver disease in Italy: a multicenter prevalence study of 9,997 cases. J Med Virol. 2005; 75(4): 522-7.	Liver cancer due to alcohol use
Italy	2001	2001	Sagnelli E, Stroffolini T, Mele A, Almasio P, Coppola N, Ferrigno L, Scolastico C, Onofrio M, Imparato M, Filippini P. The importance of HCV on the burden of chronic liver disease in Italy: a multicenter prevalence study of 9,997 cases. J Med Virol. 2005; 75(4): 522-7.	Liver cancer due to hepatitis B
Italy	2001	2001	Sagnelli E, Stroffolini T, Mele A, Almasio P, Coppola N, Ferrigno L, Scolastico C, Onofrio M, Imparato M, Filippini P. The importance of HCV on the burden of chronic liver disease in Italy: a multicenter prevalence study of 9,997 cases. J Med Virol. 2005; 75(4): 522-7.	Liver cancer due to hepatitis C
Italy	2001	2001	Sagnelli E, Stroffolini T, Mele A, Almasio P, Coppola N, Ferrigno L, Scolastico C, Onofrio M, Imparato M, Filippini P. The importance of HCV on the burden of chronic liver disease in Italy: a multicenter prevalence study of 9,997 cases. J Med Virol. 2005; 75(4): 522-7.	Liver cancer due to other causes
Italy	1987	1996	Santi V, Buccione D, Di Micoli A, Fatti G, Frigerio M, Farinati F, Del Poggio P, Rapaccini G, Di Nolfo MA, Benvegnù L, Zoli M, Borzio F, Giannini EG, Caturelli E, Chiaramonte M, Bernardi M, Trevisani F. The changing scenario of hepatocellular carcinoma over the last two decades in Italy. J Hepatol. 2012; 56(2): 397-405.	Liver cancer due to alcohol use
Italy	1987	1996	Santi V, Buccione D, Di Micoli A, Fatti G, Frigerio M, Farinati F, Del Poggio P, Rapaccini G, Di Nolfo MA, Benvegnù L, Zoli M, Borzio F, Giannini EG, Caturelli E, Chiaramonte M, Bernardi M, Trevisani F. The changing scenario of hepatocellular carcinoma over the last two decades in Italy. J Hepatol. 2012; 56(2): 397-405.	Liver cancer due to hepatitis B
Italy	1987	1996	Santi V, Buccione D, Di Micoli A, Fatti G, Frigerio M, Farinati F, Del Poggio P, Rapaccini G, Di Nolfo MA, Benvegnù L, Zoli M, Borzio F, Giannini EG, Caturelli E, Chiaramonte M, Bernardi M, Trevisani F. The changing scenario of hepatocellular carcinoma over the last two decades in Italy. J Hepatol. 2012; 56(2): 397-405.	Liver cancer due to hepatitis C
Italy	1987	1996	Santi V, Buccione D, Di Micoli A, Fatti G, Frigerio M, Farinati F, Del Poggio P, Rapaccini G, Di Nolfo MA, Benvegnù L, Zoli M, Borzio F, Giannini EG, Caturelli E, Chiaramonte M, Bernardi M, Trevisani F. The changing scenario of hepatocellular carcinoma over the last two decades in Italy. J Hepatol. 2012; 56(2): 397-405.	Liver cancer due to other causes
Italy	1997	2001	Santi V, Buccione D, Di Micoli A, Fatti G, Frigerio M, Farinati F, Del Poggio P, Rapaccini G, Di Nolfo MA, Benvegnù L, Zoli M, Borzio F, Giannini EG, Caturelli E, Chiaramonte M, Bernardi M, Trevisani F. The changing scenario of hepatocellular carcinoma over the last two decades in	Liver cancer due to alcohol use

©2017 Global Burden of Disease Liver Cancer Collaboration. JAMA Oncology.

Country	Years included in estimation (start, end)		Citation	Liver cancer etiology
			Italy. J Hepatol. 2012; 56(2): 397-405.	
Italy	1997	2001	Santi V, Buccione D, Di Micoli A, Fatti G, Frigerio M, Farinati F, Del Poggio P, Rapaccini G, Di Nolfo MA, Benvegnù L, Zoli M, Borzio F, Giannini EG, Caturelli E, Chiaramonte M, Bernardi M, Trevisani F. The changing scenario of hepatocellular carcinoma over the last two decades in Italy. J Hepatol. 2012; 56(2): 397-405.	Liver cancer due to hepatitis B
Italy	1997	2001	Santi V, Buccione D, Di Micoli A, Fatti G, Frigerio M, Farinati F, Del Poggio P, Rapaccini G, Di Nolfo MA, Benvegnù L, Zoli M, Borzio F, Giannini EG, Caturelli E, Chiaramonte M, Bernardi M, Trevisani F. The changing scenario of hepatocellular carcinoma over the last two decades in Italy. J Hepatol. 2012; 56(2): 397-405.	Liver cancer due to hepatitis C
Italy	1997	2001	Santi V, Buccione D, Di Micoli A, Fatti G, Frigerio M, Farinati F, Del Poggio P, Rapaccini G, Di Nolfo MA, Benvegnù L, Zoli M, Borzio F, Giannini EG, Caturelli E, Chiaramonte M, Bernardi M, Trevisani F. The changing scenario of hepatocellular carcinoma over the last two decades in Italy. J Hepatol. 2012; 56(2): 397-405.	Liver cancer due to other causes
Italy	2002	2008	Santi V, Buccione D, Di Micoli A, Fatti G, Frigerio M, Farinati F, Del Poggio P, Rapaccini G, Di Nolfo MA, Benvegnù L, Zoli M, Borzio F, Giannini EG, Caturelli E, Chiaramonte M, Bernardi M, Trevisani F. The changing scenario of hepatocellular carcinoma over the last two decades in Italy. J Hepatol. 2012; 56(2): 397-405.	Liver cancer due to alcohol use
Italy	2002	2008	Santi V, Buccione D, Di Micoli A, Fatti G, Frigerio M, Farinati F, Del Poggio P, Rapaccini G, Di Nolfo MA, Benvegnù L, Zoli M, Borzio F, Giannini EG, Caturelli E, Chiaramonte M, Bernardi M, Trevisani F. The changing scenario of hepatocellular carcinoma over the last two decades in Italy. J Hepatol. 2012; 56(2): 397-405.	Liver cancer due to hepatitis B
Italy	2002	2008	Santi V, Buccione D, Di Micoli A, Fatti G, Frigerio M, Farinati F, Del Poggio P, Rapaccini G, Di Nolfo MA, Benvegnù L, Zoli M, Borzio F, Giannini EG, Caturelli E, Chiaramonte M, Bernardi M, Trevisani F. The changing scenario of hepatocellular carcinoma over the last two decades in Italy. J Hepatol. 2012; 56(2): 397-405.	Liver cancer due to hepatitis C
Italy	2002	2008	Santi V, Buccione D, Di Micoli A, Fatti G, Frigerio M, Farinati F, Del Poggio P, Rapaccini G, Di Nolfo MA, Benvegnù L, Zoli M, Borzio F, Giannini EG, Caturelli E, Chiaramonte M, Bernardi M, Trevisani F. The changing scenario of hepatocellular carcinoma over the last two decades in Italy. J Hepatol. 2012; 56(2): 397-405.	Liver cancer due to other causes
Italy	1993	1998	Soresi M, La Spada E, Giannitrapani L, Campagna E, Di Gesaro V, Granà W, Sandonato L, Brancatelli G, Rotolo G, Affronti A, Messina S, Montalto G. Hepatocellular carcinoma:	Liver cancer due to

©2017 Global Burden of Disease Liver Cancer Collaboration. JAMA Oncology.

Country	Years included in estimation (start, end)		Citation	Liver cancer etiology
			comparison of two different periods at the same center. Eur J Intern Med. 2010; 21(2): 127-30.	hepatitis B
Italy	1993	1998	Soresi M, La Spada E, Giannitrapani L, Campagna E, Di Gesaro V, Granà W, Sandonato L, Brancatelli G, Rotolo G, Affronti A, Messina S, Montalto G. Hepatocellular carcinoma: comparison of two different periods at the same center. Eur J Intern Med. 2010; 21(2): 127-30.	Liver cancer due to hepatitis C
Italy	2003	2008	Soresi M, La Spada E, Giannitrapani L, Campagna E, Di Gesaro V, Granà W, Sandonato L, Brancatelli G, Rotolo G, Affronti A, Messina S, Montalto G. Hepatocellular carcinoma: comparison of two different periods at the same center. Eur J Intern Med. 2010; 21(2): 127-30.	Liver cancer due to hepatitis B
Italy	2003	2008	Soresi M, La Spada E, Giannitrapani L, Campagna E, Di Gesaro V, Granà W, Sandonato L, Brancatelli G, Rotolo G, Affronti A, Messina S, Montalto G. Hepatocellular carcinoma: comparison of two different periods at the same center. Eur J Intern Med. 2010; 21(2): 127-30.	Liver cancer due to hepatitis C
Italy	1995	1998	Tagger A, Donato F, Ribero ML, Chiesa R, Portera G, Gelatti U, Albertini A, Fasola M, Boffetta P, Nardi G. Case-control study on hepatitis C virus (HCV) as a risk factor for hepatocellular carcinoma: the role of HCV genotypes and the synergism with hepatitis B virus and alcohol. Brescia HCC Study. Int J Cancer. 1999; 81(5): 695-9.	Liver cancer due to hepatitis B
Italy	1995	1998	Tagger A, Donato F, Ribero ML, Chiesa R, Portera G, Gelatti U, Albertini A, Fasola M, Boffetta P, Nardi G. Case-control study on hepatitis C virus (HCV) as a risk factor for hepatocellular carcinoma: the role of HCV genotypes and the synergism with hepatitis B virus and alcohol. Brescia HCC Study. Int J Cancer. 1999; 81(5): 695-9.	Liver cancer due to hepatitis C
Italy	1989	2009	Zani C, Pasquale L, Bressanelli M, Puoti M, Paris B, Coccaglio R, Lascioli I, Pieriacci G, Donato F. The epidemiological pattern of chronic liver diseases in a community undergoing voluntary screening for hepatitis B and C. Dig Liver Dis. 2011; 43(8): 653-8.	Liver cancer due to alcohol use
Italy	1989	2009	Zani C, Pasquale L, Bressanelli M, Puoti M, Paris B, Coccaglio R, Lascioli I, Pieriacci G, Donato F. The epidemiological pattern of chronic liver diseases in a community undergoing voluntary screening for hepatitis B and C. Dig Liver Dis. 2011; 43(8): 653-8.	Liver cancer due to hepatitis B
Italy	1989	2009	Zani C, Pasquale L, Bressanelli M, Puoti M, Paris B, Coccaglio R, Lascioli I, Pieriacci G, Donato F. The epidemiological pattern of chronic liver diseases in a community undergoing voluntary screening for hepatitis B and C. Dig Liver Dis. 2011; 43(8): 653-8.	Liver cancer due to hepatitis C
Italy	1989	2009	Zani C, Pasquale L, Bressanelli M, Puoti M, Paris B, Coccaglio R, Lascioli I, Pieriacci G, Donato F. The epidemiological pattern of chronic liver diseases in a community undergoing voluntary screening for hepatitis B and C. Dig Liver Dis. 2011; 43(8): 653-8.	Liver cancer due to other causes
Japan	1982	2007	Akahoshi H, Taura N, Ichikawa T, Miyaaki H, Akiyama M, Miuma S, Ozawa E, Takeshita S,	Liver cancer

©2017 Global Burden of Disease Liver Cancer Collaboration. JAMA Oncology.

Country	Years included in estimation (start, end)		Citation	Liver cancer etiology
			Muraoka T, Matsuzaki T, Ohtani M, Isomoto H, Matsumoto T, Takeshima F, Nakao K. Differences in prognostic factors according to viral status in patients with hepatocellular carcinoma. Oncol Rep. 2010; 23(5): 1317-23.	due to alcohol use
Japan	1982	2007	Akahoshi H, Taura N, Ichikawa T, Miyaaki H, Akiyama M, Miuma S, Ozawa E, Takeshita S, Muraoka T, Matsuzaki T, Ohtani M, Isomoto H, Matsumoto T, Takeshima F, Nakao K. Differences in prognostic factors according to viral status in patients with hepatocellular carcinoma. Oncol Rep. 2010; 23(5): 1317-23.	Liver cancer due to hepatitis B
Japan	1982	2007	Akahoshi H, Taura N, Ichikawa T, Miyaaki H, Akiyama M, Miuma S, Ozawa E, Takeshita S, Muraoka T, Matsuzaki T, Ohtani M, Isomoto H, Matsumoto T, Takeshima F, Nakao K. Differences in prognostic factors according to viral status in patients with hepatocellular carcinoma. Oncol Rep. 2010; 23(5): 1317-23.	Liver cancer due to hepatitis C
Japan	1982	2007	Akahoshi H, Taura N, Ichikawa T, Miyaaki H, Akiyama M, Miuma S, Ozawa E, Takeshita S, Muraoka T, Matsuzaki T, Ohtani M, Isomoto H, Matsumoto T, Takeshima F, Nakao K. Differences in prognostic factors according to viral status in patients with hepatocellular carcinoma. Oncol Rep. 2010; 23(5): 1317-23.	Liver cancer due to other causes
Japan	1994	1994	Koide T, Ohno T, Huang XE, Iijima Y, Sugihara K, Mizokami M, Xiang J, Tokudome S. HBV/HCV Infection, Alcohol, Tobacco and Genetic Polymorphisms for Hepatocellular Carcinoma in Nagoya, Japan. Asian Pac J Cancer Prev. 2000; 1(3): 237-43.	Liver cancer due to hepatitis B
Japan	1994	1994	Koide T, Ohno T, Huang XE, Iijima Y, Sugihara K, Mizokami M, Xiang J, Tokudome S. HBV/HCV Infection, Alcohol, Tobacco and Genetic Polymorphisms for Hepatocellular Carcinoma in Nagoya, Japan. Asian Pac J Cancer Prev. 2000; 1(3): 237-43.	Liver cancer due to hepatitis C
Japan	1990	1997	Kubo S, Nishiguchi S, Hirohashi K, Tanaka H, Tsukamoto T, Hamba H, Shuto T, Okuda T, Tamori A, Kuroki T, Kinoshita H. High prevalence of infection with hepatitis B and C viruses in patients with hepatocellular carcinoma in Japan. Hepatogastroenterology. 1999; 46(25): 357-9.	Liver cancer due to hepatitis B
Japan	1990	1997	Kubo S, Nishiguchi S, Hirohashi K, Tanaka H, Tsukamoto T, Hamba H, Shuto T, Okuda T, Tamori A, Kuroki T, Kinoshita H. High prevalence of infection with hepatitis B and C viruses in patients with hepatocellular carcinoma in Japan. Hepatogastroenterology. 1999; 46(25): 357-9.	Liver cancer due to hepatitis C
Japan	2006	2008	Michitaka K, Nishiguchi S, Aoyagi Y, Hiasa Y, Tokumoto Y, Onji M, Japan Etiology of Liver Cirrhosis Study Group. Etiology of liver cirrhosis in Japan: a nationwide survey. J Gastroenterol. 2010; 45(1): 86-94.	Liver cancer due to alcohol use
Japan	2006	2008	Michitaka K, Nishiguchi S, Aoyagi Y, Hiasa Y, Tokumoto Y, Onji M, Japan Etiology of Liver	Liver cancer

©2017 Global Burden of Disease Liver Cancer Collaboration. JAMA Oncology.

Country	Years included in estimation (start, end)		Citation	Liver cancer etiology
			Cirrhosis Study Group. Etiology of liver cirrhosis in Japan: a nationwide survey. J Gastroenterol. 2010; 45(1): 86-94.	due to hepatitis B
Japan	2006	2008	Michitaka K, Nishiguchi S, Aoyagi Y, Hiasa Y, Tokumoto Y, Onji M, Japan Etiology of Liver Cirrhosis Study Group. Etiology of liver cirrhosis in Japan: a nationwide survey. J Gastroenterol. 2010; 45(1): 86-94.	Liver cancer due to hepatitis C
Japan	2006	2008	Michitaka K, Nishiguchi S, Aoyagi Y, Hiasa Y, Tokumoto Y, Onji M, Japan Etiology of Liver Cirrhosis Study Group. Etiology of liver cirrhosis in Japan: a nationwide survey. J Gastroenterol. 2010; 45(1): 86-94.	Liver cancer due to other causes
Japan	1995	2009	Nagaoki Y, Hyogo H, Aikata H, Tanaka M, Naeshiro N, Nakahara T, Honda Y, Miyaki D, Kawaoka T, Takaki S, Hiramatsu A, Waki K, Imamura M, Kawakami Y, Takahashi S, Chayama K. Recent trend of clinical features in patients with hepatocellular carcinoma. Hepatol Res. 2012; 42(4): 368-75.	Liver cancer due to alcohol use
Japan	1995	2009	Nagaoki Y, Hyogo H, Aikata H, Tanaka M, Naeshiro N, Nakahara T, Honda Y, Miyaki D, Kawaoka T, Takaki S, Hiramatsu A, Waki K, Imamura M, Kawakami Y, Takahashi S, Chayama K. Recent trend of clinical features in patients with hepatocellular carcinoma. Hepatol Res. 2012; 42(4): 368-75.	Liver cancer due to hepatitis B
Japan	1995	2009	Nagaoki Y, Hyogo H, Aikata H, Tanaka M, Naeshiro N, Nakahara T, Honda Y, Miyaki D, Kawaoka T, Takaki S, Hiramatsu A, Waki K, Imamura M, Kawakami Y, Takahashi S, Chayama K. Recent trend of clinical features in patients with hepatocellular carcinoma. Hepatol Res. 2012; 42(4): 368-75.	Liver cancer due to hepatitis C
Japan	1995	2009	Nagaoki Y, Hyogo H, Aikata H, Tanaka M, Naeshiro N, Nakahara T, Honda Y, Miyaki D, Kawaoka T, Takaki S, Hiramatsu A, Waki K, Imamura M, Kawakami Y, Takahashi S, Chayama K. Recent trend of clinical features in patients with hepatocellular carcinoma. Hepatol Res. 2012; 42(4): 368-75.	Liver cancer due to other causes
Japan	1990	1992	Tanaka H, Imai Y, Hiramatsu N, Ito Y, Imanaka K, Oshita M, Hijioka T, Katayama K, Yabuuchi I, Yoshihara H, Inoue A, Kato M, Takehara T, Tamura S, Kasahara A, Hayashi N, Tsukuma H. Declining incidence of hepatocellular carcinoma in Osaka, Japan, from 1990 to 2003. Ann Intern Med. 2008; 148(11): 820-6.	Liver cancer due to hepatitis B
Japan	1990	1992	Tanaka H, Imai Y, Hiramatsu N, Ito Y, Imanaka K, Oshita M, Hijioka T, Katayama K, Yabuuchi I, Yoshihara H, Inoue A, Kato M, Takehara T, Tamura S, Kasahara A, Hayashi N, Tsukuma H. Declining incidence of hepatocellular carcinoma in Osaka, Japan, from 1990 to 2003. Ann	Liver cancer due to hepatitis C

©2017 Global Burden of Disease Liver Cancer Collaboration. JAMA Oncology.

Country	Years included in estimation (start, end)		Citation	Liver cancer etiology
			Intern Med. 2008; 148(11): 820-6.	
Japan	1993	1995	Tanaka H, Imai Y, Hiramatsu N, Ito Y, Imanaka K, Oshita M, Hijioka T, Katayama K, Yabuuchi I, Yoshihara H, Inoue A, Kato M, Takehara T, Tamura S, Kasahara A, Hayashi N, Tsukuma H. Declining incidence of hepatocellular carcinoma in Osaka, Japan, from 1990 to 2003. Ann Intern Med. 2008; 148(11): 820-6.	Liver cancer due to hepatitis B
Japan	1993	1995	Tanaka H, Imai Y, Hiramatsu N, Ito Y, Imanaka K, Oshita M, Hijioka T, Katayama K, Yabuuchi I, Yoshihara H, Inoue A, Kato M, Takehara T, Tamura S, Kasahara A, Hayashi N, Tsukuma H. Declining incidence of hepatocellular carcinoma in Osaka, Japan, from 1990 to 2003. Ann Intern Med. 2008; 148(11): 820-6.	Liver cancer due to hepatitis C
Japan	1996	1998	Tanaka H, Imai Y, Hiramatsu N, Ito Y, Imanaka K, Oshita M, Hijioka T, Katayama K, Yabuuchi I, Yoshihara H, Inoue A, Kato M, Takehara T, Tamura S, Kasahara A, Hayashi N, Tsukuma H. Declining incidence of hepatocellular carcinoma in Osaka, Japan, from 1990 to 2003. Ann Intern Med. 2008; 148(11): 820-6.	Liver cancer due to hepatitis B
Japan	1996	1998	Tanaka H, Imai Y, Hiramatsu N, Ito Y, Imanaka K, Oshita M, Hijioka T, Katayama K, Yabuuchi I, Yoshihara H, Inoue A, Kato M, Takehara T, Tamura S, Kasahara A, Hayashi N, Tsukuma H. Declining incidence of hepatocellular carcinoma in Osaka, Japan, from 1990 to 2003. Ann Intern Med. 2008; 148(11): 820-6.	Liver cancer due to hepatitis C
Japan	1999	2001	Tanaka H, Imai Y, Hiramatsu N, Ito Y, Imanaka K, Oshita M, Hijioka T, Katayama K, Yabuuchi I, Yoshihara H, Inoue A, Kato M, Takehara T, Tamura S, Kasahara A, Hayashi N, Tsukuma H. Declining incidence of hepatocellular carcinoma in Osaka, Japan, from 1990 to 2003. Ann Intern Med. 2008; 148(11): 820-6.	Liver cancer due to hepatitis B
Japan	1999	2001	Tanaka H, Imai Y, Hiramatsu N, Ito Y, Imanaka K, Oshita M, Hijioka T, Katayama K, Yabuuchi I, Yoshihara H, Inoue A, Kato M, Takehara T, Tamura S, Kasahara A, Hayashi N, Tsukuma H. Declining incidence of hepatocellular carcinoma in Osaka, Japan, from 1990 to 2003. Ann Intern Med. 2008; 148(11): 820-6.	Liver cancer due to hepatitis C
Japan	2002	2003	Tanaka H, Imai Y, Hiramatsu N, Ito Y, Imanaka K, Oshita M, Hijioka T, Katayama K, Yabuuchi I, Yoshihara H, Inoue A, Kato M, Takehara T, Tamura S, Kasahara A, Hayashi N, Tsukuma H. Declining incidence of hepatocellular carcinoma in Osaka, Japan, from 1990 to 2003. Ann Intern Med. 2008; 148(11): 820-6.	Liver cancer due to hepatitis B
Japan	2002	2003	Tanaka H, Imai Y, Hiramatsu N, Ito Y, Imanaka K, Oshita M, Hijioka T, Katayama K, Yabuuchi I, Yoshihara H, Inoue A, Kato M, Takehara T, Tamura S, Kasahara A, Hayashi N, Tsukuma H.	Liver cancer due to

©2017 Global Burden of Disease Liver Cancer Collaboration. JAMA Oncology.

Country	Years included in estimation (start, end)		Citation	Liver cancer etiology
			Declining incidence of hepatocellular carcinoma in Osaka, Japan, from 1990 to 2003. Ann Intern Med. 2008; 148(11): 820-6.	hepatitis C
Japan	1996	2008	Taura N, Fukushima N, Yastuhashi H, Takami Y, Seike M, Watanabe H, Mizuta T, Sasaki Y, Nagata K, Tabara A, Komorizono Y, Taketomi A, Matsumoto S, Tamai T, Muro T, Nakao K, Fukuizumi K, Maeshiro T, Inoue O, Sata M. The incidence of hepatocellular carcinoma associated with hepatitis C infection decreased in Kyushu area. Med Sci Monit. 2011; 17(2): PH7-11.	Liver cancer due to hepatitis B
Japan	1996	2008	Taura N, Fukushima N, Yastuhashi H, Takami Y, Seike M, Watanabe H, Mizuta T, Sasaki Y, Nagata K, Tabara A, Komorizono Y, Taketomi A, Matsumoto S, Tamai T, Muro T, Nakao K, Fukuizumi K, Maeshiro T, Inoue O, Sata M. The incidence of hepatocellular carcinoma associated with hepatitis C infection decreased in Kyushu area. Med Sci Monit. 2011; 17(2): PH7-11.	Liver cancer due to hepatitis C
Japan	2006	2009	Tokushige K, Hashimoto E, Horie Y, Taniai M, Higuchi S. Hepatocellular carcinoma in Japanese patients with nonalcoholic fatty liver disease, alcoholic liver disease, and chronic liver disease of unknown etiology: report of the nationwide survey. J Gastroenterol. 2011; 46(10): 1230-7.	Liver cancer due to alcohol use
Japan	2006	2009	Tokushige K, Hashimoto E, Horie Y, Taniai M, Higuchi S. Hepatocellular carcinoma in Japanese patients with nonalcoholic fatty liver disease, alcoholic liver disease, and chronic liver disease of unknown etiology: report of the nationwide survey. J Gastroenterol. 2011; 46(10): 1230-7.	Liver cancer due to hepatitis B
Japan	2006	2009	Tokushige K, Hashimoto E, Horie Y, Taniai M, Higuchi S. Hepatocellular carcinoma in Japanese patients with nonalcoholic fatty liver disease, alcoholic liver disease, and chronic liver disease of unknown etiology: report of the nationwide survey. J Gastroenterol. 2011; 46(10): 1230-7.	Liver cancer due to hepatitis C
Japan	2006	2009	Tokushige K, Hashimoto E, Horie Y, Taniai M, Higuchi S. Hepatocellular carcinoma in Japanese patients with nonalcoholic fatty liver disease, alcoholic liver disease, and chronic liver disease of unknown etiology: report of the nationwide survey. J Gastroenterol. 2011; 46(10): 1230-7.	Liver cancer due to other causes
Japan	2000	2002	Wang B-E, Ma W-M, Sulaiman A, Noer S, Sumoharjo S, Sumarsidi D, Tandon BN, Nakao K, Mishiro S, Miyakawa Y, Akahane Y, Suzuki H. Demographic, clinical, and virological characteristics of hepatocellular carcinoma in Asia: survey of 414 patients from four countries. J Med Virol. 2002; 67(3): 394-400.	Liver cancer due to hepatitis B
Japan	2000	2002	Wang B-E, Ma W-M, Sulaiman A, Noer S, Sumoharjo S, Sumarsidi D, Tandon BN, Nakao K, Mishiro S, Miyakawa Y, Akahane Y, Suzuki H. Demographic, clinical, and virological characteristics of hepatocellular carcinoma in Asia: survey of 414 patients from four countries.	Liver cancer due to hepatitis C

©2017 Global Burden of Disease Liver Cancer Collaboration. JAMA Oncology.

Country	Years included in estimation (start, end)		Citation	Liver cancer etiology
			J Med Virol. 2002; 67(3): 394-400.	
Japan	2000	2002	Wang B-E, Ma W-M, Sulaiman A, Noer S, Sumoharjo S, Sumarsidi D, Tandon BN, Nakao K, Mishiro S, Miyakawa Y, Akahane Y, Suzuki H. Demographic, clinical, and virological characteristics of hepatocellular carcinoma in Asia: survey of 414 patients from four countries. J Med Virol. 2002; 67(3): 394-400.	Liver cancer due to other causes
Japan	1993	1999	Watabe H, Shiratori Y, Tateishi R, Fujishima T, Akamatsu M, Koike Y, Obi S, Hamamura K, Sato S, Teratani T, Shiina S, Omata M. Clinical features of patients with HCC who are negative for both HBV and HCV markers. Hepatogastroenterology. 2003; 50(54): 2157-60.	Liver cancer due to hepatitis B
Japan	1993	1999	Watabe H, Shiratori Y, Tateishi R, Fujishima T, Akamatsu M, Koike Y, Obi S, Hamamura K, Sato S, Teratani T, Shiina S, Omata M. Clinical features of patients with HCC who are negative for both HBV and HCV markers. Hepatogastroenterology. 2003; 50(54): 2157-60.	Liver cancer due to hepatitis C
Kenya	1992	1994	Ilako FM, McLigeyo SO, Riyat MS, Lule GN, Okoth FA, Kaptich D. The prevalence of hepatitis C virus antibodies in renal patients, blood donors and patients with chronic liver disease in Kenya. East Afr Med J. 1995; 72(6): 362-4.	Liver cancer due to hepatitis B
Kenya	1992	1994	Ilako FM, McLigeyo SO, Riyat MS, Lule GN, Okoth FA, Kaptich D. The prevalence of hepatitis C virus antibodies in renal patients, blood donors and patients with chronic liver disease in Kenya. East Afr Med J. 1995; 72(6): 362-4.	Liver cancer due to hepatitis C
Lebanon	1998	2003	Yaghi C, Sharara A-I, Rassam P, Moucari R, Honein K, BouJaoude J, Slim R, Noun R, Abdul-Baki H, Khalifeh M, Ramia S, Sayegh R. Hepatocellular carcinoma in Lebanon: Etiology and prognostic factors associated with short-term survival. World J Gastroenterol. 2006; 12(22): 3575-80.	Liver cancer due to alcohol use
Lebanon	1998	2003	Yaghi C, Sharara A-I, Rassam P, Moucari R, Honein K, BouJaoude J, Slim R, Noun R, Abdul-Baki H, Khalifeh M, Ramia S, Sayegh R. Hepatocellular carcinoma in Lebanon: Etiology and prognostic factors associated with short-term survival. World J Gastroenterol. 2006; 12(22): 3575-80.	Liver cancer due to hepatitis B
Lebanon	1998	2003	Yaghi C, Sharara A-I, Rassam P, Moucari R, Honein K, BouJaoude J, Slim R, Noun R, Abdul-Baki H, Khalifeh M, Ramia S, Sayegh R. Hepatocellular carcinoma in Lebanon: Etiology and prognostic factors associated with short-term survival. World J Gastroenterol. 2006; 12(22): 3575-80.	Liver cancer due to hepatitis C
Malaysia	2006	2009	Qua C-S, Goh K-L. Liver cirrhosis in Malaysia: peculiar epidemiology in a multiracial Asian country. J Gastroenterol Hepatol. 2011; 26(8): 1333-7.	Liver cancer due to

©2017 Global Burden of Disease Liver Cancer Collaboration. JAMA Oncology.

Country	Years included in estimation (start, end)		Citation	Liver cancer etiology
				hepatitis B
Malaysia	2006	2009	Qua C-S, Goh K-L. Liver cirrhosis in Malaysia: peculiar epidemiology in a multiracial Asian country. J Gastroenterol Hepatol. 2011; 26(8): 1333-7.	Liver cancer due to hepatitis C
Malaysia	2006	2009	Qua C-S, Goh K-L. Liver cirrhosis in Malaysia: peculiar epidemiology in a multiracial Asian country. J Gastroenterol Hepatol. 2011; 26(8): 1333-7.	Liver cancer due to other causes
Mali	1998	1999	Dembele M, Maïga I, Minta D, Konate A, Diarra M, Sangare D, Traore HA, Maïga MY, Tounkara A, Payan C, Lunel E, Carbonnelle B, Cales P. [Study of antigen HBs and antivirus antibodies of hepatitis C during hepatopathies in Mali]. Bull Soc Pathol Exot. 2004; 97(3): 161-4.	Liver cancer due to hepatitis B
Mali	1998	1999	Dembele M, Maïga I, Minta D, Konate A, Diarra M, Sangare D, Traore HA, Maïga MY, Tounkara A, Payan C, Lunel E, Carbonnelle B, Cales P. [Study of antigen HBs and antivirus antibodies of hepatitis C during hepatopathies in Mali]. Bull Soc Pathol Exot. 2004; 97(3): 161-4.	Liver cancer due to hepatitis C
Mexico	1992	2002	Mondragón-Sánchez R, Garduño-López AL, Hernández-Castillo E, Gómez-Gómez E, Ruiz-Molina JM. Hepatocellular carcinoma and hepatitis C in Mexico. Hepatogastroenterology. 2005; 52(64): 1159-62.	Liver cancer due to alcohol use
Mexico	1992	2002	Mondragón-Sánchez R, Garduño-López AL, Hernández-Castillo E, Gómez-Gómez E, Ruiz-Molina JM. Hepatocellular carcinoma and hepatitis C in Mexico. Hepatogastroenterology. 2005; 52(64): 1159-62.	Liver cancer due to hepatitis B
Mexico	1992	2002	Mondragón-Sánchez R, Garduño-López AL, Hernández-Castillo E, Gómez-Gómez E, Ruiz-Molina JM. Hepatocellular carcinoma and hepatitis C in Mexico. Hepatogastroenterology. 2005; 52(64): 1159-62.	Liver cancer due to hepatitis C
Mexico	1992	2002	Mondragón-Sánchez R, Garduño-López AL, Hernández-Castillo E, Gómez-Gómez E, Ruiz-Molina JM. Hepatocellular carcinoma and hepatitis C in Mexico. Hepatogastroenterology. 2005; 52(64): 1159-62.	Liver cancer due to other causes
Mongolia	2005	2005	Oyunsuren T, Kurbanov F, Tanaka Y, Elkady A, Sanduijav R, Khajidsuren O, Dagvadorj B, Mizokami M. High frequency of hepatocellular carcinoma in Mongolia; association with mono-, or co-infection with hepatitis C, B, and delta viruses. J Med Virol. 2006; 78(12): 1688-95.	Liver cancer due to hepatitis B
Mongolia	2005	2005	Oyunsuren T, Kurbanov F, Tanaka Y, Elkady A, Sanduijav R, Khajidsuren O, Dagvadorj B, Mizokami M. High frequency of hepatocellular carcinoma in Mongolia; association with mono-, or co-infection with hepatitis C, B, and delta viruses. J Med Virol. 2006; 78(12): 1688-95.	Liver cancer due to hepatitis C

©2017 Global Burden of Disease Liver Cancer Collaboration. JAMA Oncology.

Country	Years included in estimation (start, end)		Citation	Liver cancer etiology
Mongolia	2004	2004	Tsatsralt-Od B, Takahashi M, Nishizawa T, Endo K, Inoue J, Okamoto H. High prevalence of dual or triple infection of hepatitis B, C, and delta viruses among patients with chronic liver disease in Mongolia. J Med Virol. 2005; 77(4): 491-9.	Liver cancer due to hepatitis B
Mongolia	2004	2004	Tsatsralt-Od B, Takahashi M, Nishizawa T, Endo K, Inoue J, Okamoto H. High prevalence of dual or triple infection of hepatitis B, C, and delta viruses among patients with chronic liver disease in Mongolia. J Med Virol. 2005; 77(4): 491-9.	Liver cancer due to hepatitis C
Nigeria	1999	2005	Nwokediuko SC, Ijoma UN, Obienu O. Liver Cancer in Enugu, South East Nigeria. Insight Bioinforma. 2011; 1(1): 1-5.	Liver cancer due to hepatitis B
Nigeria	1999	2005	Nwokediuko SC, Ijoma UN, Obienu O. Liver Cancer in Enugu, South East Nigeria. Insight Bioinforma. 2011; 1(1): 1-5.	Liver cancer due to hepatitis C
Nigeria	1993	1994	Ojo OS, Thursz M, Thomas HC, Ndububa DA, Adeodu OO, Rotimi O, Lawal AA, Durosinmi MA, Akonai AK, Fatusi AO. Hepatitis B virus markers, hepatitis D virus antigen and hepatitis C virus antibodies in Nigerian patients with chronic liver disease. East Afr Med J. 1995; 72(11): 719-21.	Liver cancer due to hepatitis B
Nigeria	1993	1994	Ojo OS, Thursz M, Thomas HC, Ndububa DA, Adeodu OO, Rotimi O, Lawal AA, Durosinmi MA, Akonai AK, Fatusi AO. Hepatitis B virus markers, hepatitis D virus antigen and hepatitis C virus antibodies in Nigerian patients with chronic liver disease. East Afr Med J. 1995; 72(11): 719-21.	Liver cancer due to hepatitis C
Nigeria	2007	2008	Okonkwo UC, Nwosu MN, Ukah C, Okpala OC, Ahaneku JI. The clinical and pathological features of hepatocellular carcinoma in Nnewi, Nigeria. Niger J Med. 2011; 20(3): 366-71.	Liver cancer due to hepatitis B
Nigeria	2007	2008	Okonkwo UC, Nwosu MN, Ukah C, Okpala OC, Ahaneku JI. The clinical and pathological features of hepatocellular carcinoma in Nnewi, Nigeria. Niger J Med. 2011; 20(3): 366-71.	Liver cancer due to hepatitis C
Nigeria	1995	1995	Olubuyide IO, Aliyu B, Olalelye OA, Ola SO, Olawuyi F, Malabu UH, Odemuyiwa SO, Odaibo GN, Cook GC. Hepatitis B and C virus and hepatocellular carcinoma. Trans R Soc Trop Med Hyg. 1997; 91(1): 38-41.	Liver cancer due to hepatitis B
Nigeria	1995	1995	Olubuyide IO, Aliyu B, Olalelye OA, Ola SO, Olawuyi F, Malabu UH, Odemuyiwa SO, Odaibo GN, Cook GC. Hepatitis B and C virus and hepatocellular carcinoma. Trans R Soc Trop Med Hyg. 1997; 91(1): 38-41.	Liver cancer due to hepatitis C
Pakistan	2005	2007	Alam JM, Mahmood SR, Shaheen R, Asghar SS. Hepatitis "B" and "C" viral infections in patients	Liver cancer

©2017 Global Burden of Disease Liver Cancer Collaboration. JAMA Oncology.

Country	Years included in estimation (start, end)		Citation	Liver cancer etiology
			with hepatocellular carcinoma. Pak J Pharmacol. 2009; 26(2): 25-32.	due to hepatitis B
Pakistan	2005	2007	Alam JM, Mahmood SR, Shaheen R, Asghar SS. Hepatitis "B" and "C" viral infections in patients with hepatocellular carcinoma. Pak J Pharmacol. 2009; 26(2): 25-32.	Liver cancer due to hepatitis C
Pakistan	2006	2007	Khan A, Tanaka Y, Azam Z, Abbas Z, Kurbanov F, Saleem U, Hamid S, Jafri W, Mizokami M. Epidemic spread of hepatitis C virus genotype 3a and relation to high incidence of hepatocellular carcinoma in Pakistan. J Med Virol. 2009; 81(7): 1189-97.	Liver cancer due to hepatitis B
Pakistan	2006	2007	Khan A, Tanaka Y, Azam Z, Abbas Z, Kurbanov F, Saleem U, Hamid S, Jafri W, Mizokami M. Epidemic spread of hepatitis C virus genotype 3a and relation to high incidence of hepatocellular carcinoma in Pakistan. J Med Virol. 2009; 81(7): 1189-97.	Liver cancer due to hepatitis C
Pakistan	1994	2000	Khokhar N. Spectrum of chronic liver disease in a tertiary care hospital. J Pak Med Assoc. 2002; 52(2): 56-8.	Liver cancer due to hepatitis B
Pakistan	1994	2000	Khokhar N. Spectrum of chronic liver disease in a tertiary care hospital. J Pak Med Assoc. 2002; 52(2): 56-8.	Liver cancer due to hepatitis C
Peru	1995	1998	Ruiz E, Almonte M M, Pizarro R, Celis J, Montalbelti JA, Urbano R. [Hepatitis B and C virus infection as risk factors for hepatocarcinoma in Peru: case and control study]. Rev Gastroenterol Peru. 1998; 18(3): 199-212.	Liver cancer due to hepatitis B
Peru	1995	1998	Ruiz E, Almonte M M, Pizarro R, Celis J, Montalbelti JA, Urbano R. [Hepatitis B and C virus infection as risk factors for hepatocarcinoma in Peru: case and control study]. Rev Gastroenterol Peru. 1998; 18(3): 199-212.	Liver cancer due to hepatitis C
Saudi Arabia	1995	1998	Ayoola EA, Gadour MOEH. Hepatocellular carcinoma in Saudi Arabia: role of hepatitis B and C infection. J Gastroenterol Hepatol. 2004; 19(6): 665-9.	Liver cancer due to hepatitis B
Saudi Arabia	1995	1998	Ayoola EA, Gadour MOEH. Hepatocellular carcinoma in Saudi Arabia: role of hepatitis B and C infection. J Gastroenterol Hepatol. 2004; 19(6): 665-9.	Liver cancer due to hepatitis C
Senegal	1995	1995	Mbaye PS, Renaudineau Y, Diallo A, Haudrechy D, Sane M, Michel G, Raphenon G, Klotz F. [Hepatitis C virus and chronic hepatopathies in Dakar: case-control study]. Med Trop (Mars).	Liver cancer due to

©2017 Global Burden of Disease Liver Cancer Collaboration. JAMA Oncology.

Country	Years included in estimation (start, end)		Citation	Liver cancer etiology
			2000; 60(1): 47-52.	hepatitis B
Senegal	1995	1995	Mbaye PS, Renaudineau Y, Diallo A, Haudrechy D, Sane M, Michel G, Raphenon G, Klotz F. [Hepatitis C virus and chronic hepatopathies in Dakar: case-control study]. Med Trop (Mars). 2000; 60(1): 47-52.	Liver cancer due to hepatitis C
Serbia	2004	2007	Kanazir M, Boricic I, Delic D, Tepavcevic DK, Knezevic A, Jovanovic T, Pekmezovic T. Risk factors for hepatocellular carcinoma: a case-control study in Belgrade (Serbia). Tumori. 2010; 96(6): 911-7.	Liver cancer due to hepatitis B
Serbia	2004	2007	Kanazir M, Boricic I, Delic D, Tepavcevic DK, Knezevic A, Jovanovic T, Pekmezovic T. Risk factors for hepatocellular carcinoma: a case-control study in Belgrade (Serbia). Tumori. 2010; 96(6): 911-7.	Liver cancer due to hepatitis C
Singapore	1990	1993	Khin LW, Teo CJ, Guan R. Seroprevalence of hepatitis B and C viral markers in patients with primary hepatocellular carcinoma in Singapore. Singapore Med J. 1996; 37(5): 492-6.	Liver cancer due to hepatitis B
Singapore	1990	1993	Khin LW, Teo CJ, Guan R. Seroprevalence of hepatitis B and C viral markers in patients with primary hepatocellular carcinoma in Singapore. Singapore Med J. 1996; 37(5): 492-6.	Liver cancer due to hepatitis C
South Africa	1991	1992	Soni PN, Tait DR, Gopaul W, Sathar MA, Simjee AE. Hepatitis C virus infection in chronic liver disease in Natal. S Afr Med J. 1996; 86(1): 80-3.	Liver cancer due to hepatitis B
South Africa	1991	1992	Soni PN, Tait DR, Gopaul W, Sathar MA, Simjee AE. Hepatitis C virus infection in chronic liver disease in Natal. S Afr Med J. 1996; 86(1): 80-3.	Liver cancer due to hepatitis C
South Korea	2004	2009	Kim BH, Park JW, Nam BH, Kwak HW, Kim WR. Validation of a model to estimate survival in ambulatory patients with hepatocellular carcinoma: a single-centre cohort study. Liver Int. 2014; 34(7): e317-23.	Liver cancer due to alcohol use
South Korea	2004	2009	Kim BH, Park JW, Nam BH, Kwak HW, Kim WR. Validation of a model to estimate survival in ambulatory patients with hepatocellular carcinoma: a single-centre cohort study. Liver Int. 2014; 34(7): e317-23.	Liver cancer due to hepatitis B
South Korea	2004	2009	Kim BH, Park JW, Nam BH, Kwak HW, Kim WR. Validation of a model to estimate survival in ambulatory patients with hepatocellular carcinoma: a single-centre cohort study. Liver Int. 2014; 34(7): e317-23.	Liver cancer due to hepatitis C

©2017 Global Burden of Disease Liver Cancer Collaboration. JAMA Oncology.

Country	Years included in estimation (start, end)		Citation	Liver cancer etiology
South Korea	2004	2009	Kim BH, Park JW, Nam BH, Kwak HW, Kim WR. Validation of a model to estimate survival in ambulatory patients with hepatocellular carcinoma: a single-centre cohort study. Liver Int. 2014; 34(7): e317-23.	Liver cancer due to other causes
Mexico	2003	2012	Ladrón de Guevara L, Rojas-Macuil P, Sánchez-Chávez X, Rossano-García A, Gorraez-de-la-Mora MT, Cervantes-Sánchez G, Orozco-Vázquez J, Lemus-Velázquez M, Rosas-Zúñiga L, Erazo-Valle A, Di-Silvio M. Hepatocellular carcinoma: epidemiological profile from a cohort of federal employees in Mexico. Ann Hepatol. 2009; 8(3): 212-9.	Liver cancer due to alcohol use
Mexico	2003	2012	Ladrón de Guevara L, Rojas-Macuil P, Sánchez-Chávez X, Rossano-García A, Gorraez-de-la-Mora MT, Cervantes-Sánchez G, Orozco-Vázquez J, Lemus-Velázquez M, Rosas-Zúñiga L, Erazo-Valle A, Di-Silvio M. Hepatocellular carcinoma: epidemiological profile from a cohort of federal employees in Mexico. Ann Hepatol. 2009; 8(3): 212-9.	Liver cancer due to hepatitis B
Mexico	2003	2012	Ladrón de Guevara L, Rojas-Macuil P, Sánchez-Chávez X, Rossano-García A, Gorraez-de-la-Mora MT, Cervantes-Sánchez G, Orozco-Vázquez J, Lemus-Velázquez M, Rosas-Zúñiga L, Erazo-Valle A, Di-Silvio M. Hepatocellular carcinoma: epidemiological profile from a cohort of federal employees in Mexico. Ann Hepatol. 2009; 8(3): 212-9.	Liver cancer due to hepatitis C
Mexico	2003	2012	Ladrón de Guevara L, Rojas-Macuil P, Sánchez-Chávez X, Rossano-García A, Gorraez-de-la-Mora MT, Cervantes-Sánchez G, Orozco-Vázquez J, Lemus-Velázquez M, Rosas-Zúñiga L, Erazo-Valle A, Di-Silvio M. Hepatocellular carcinoma: epidemiological profile from a cohort of federal employees in Mexico. Ann Hepatol. 2009; 8(3): 212-9.	Liver cancer due to other causes
South Korea	2003	2012	Lee SS, Jeong S-H, Byoun Y-S, Chung SM, Seong MH, Sohn HR, Min B-Y, Jang ES, Kim J-W, Park GJ, Lee YJ, Lee KH, Ahn S. Clinical features and outcome of cryptogenic hepatocellular carcinoma compared to those of viral and alcoholic hepatocellular carcinoma. BMC Cancer. 2013; 335.	Liver cancer due to alcohol use
South Korea	2003	2012	Lee SS, Jeong S-H, Byoun Y-S, Chung SM, Seong MH, Sohn HR, Min B-Y, Jang ES, Kim J-W, Park GJ, Lee YJ, Lee KH, Ahn S. Clinical features and outcome of cryptogenic hepatocellular carcinoma compared to those of viral and alcoholic hepatocellular carcinoma. BMC Cancer. 2013; 335.	Liver cancer due to hepatitis B
South Korea	2003	2012	Lee SS, Jeong S-H, Byoun Y-S, Chung SM, Seong MH, Sohn HR, Min B-Y, Jang ES, Kim J-W, Park GJ, Lee YJ, Lee KH, Ahn S. Clinical features and outcome of cryptogenic hepatocellular carcinoma compared to those of viral and alcoholic hepatocellular carcinoma. BMC Cancer. 2013; 335.	Liver cancer due to hepatitis C

©2017 Global Burden of Disease Liver Cancer Collaboration. JAMA Oncology.

Country	Years included in estimation (start, end)		Citation	Liver cancer etiology
South Korea	2003	2012	Lee SS, Jeong S-H, Byoun Y-S, Chung SM, Seong MH, Sohn HR, Min B-Y, Jang ES, Kim J-W, Park GJ, Lee YJ, Lee KH, Ahn S. Clinical features and outcome of cryptogenic hepatocellular carcinoma compared to those of viral and alcoholic hepatocellular carcinoma. BMC Cancer. 2013; 335.	Liver cancer due to other causes
South Korea	1995	2000	Shim J, Kim B-H, Kim NH, Dong SH, Kim HJ, Chang YW, Lee JI, Chang R. Clinical features of HBsAg-negative but anti-HBc-positive hepatocellular carcinoma in a hepatitis B virus endemic area. J Gastroenterol Hepatol. 2005; 20(5): 746-51.	Liver cancer due to hepatitis B
South Korea	1995	2000	Shim J, Kim B-H, Kim NH, Dong SH, Kim HJ, Chang YW, Lee JI, Chang R. Clinical features of HBsAg-negative but anti-HBc-positive hepatocellular carcinoma in a hepatitis B virus endemic area. J Gastroenterol Hepatol. 2005; 20(5): 746-51.	Liver cancer due to hepatitis C
Spain	2000	2000	García-Torres ML, Zaragozá A, Giner R, Primo J, del Olmo JA. Incidence and epidemiological factors of hepatocellular carcinoma in Valencia during the year 2000. Rev Esp Enferm Dig. 2003; 95(6): 381-8.	Liver cancer due to hepatitis B
Spain	2000	2000	García-Torres ML, Zaragozá A, Giner R, Primo J, del Olmo JA. Incidence and epidemiological factors of hepatocellular carcinoma in Valencia during the year 2000. Rev Esp Enferm Dig. 2003; 95(6): 381-8.	Liver cancer due to hepatitis C
Spain	2000	2000	García-Torres ML, Zaragozá A, Giner R, Primo J, del Olmo JA. Incidence and epidemiological factors of hepatocellular carcinoma in Valencia during the year 2000. Rev Esp Enferm Dig. 2003; 95(6): 381-8.	Liver cancer due to other causes
Spain	1990	1994	Macías Rodríguez MA, Rendón Unceta P, Tejada Cabrera M, Infante Hernández JM, Correro Aguilar F, Díaz García F, Benítez Rodríguez E, Mangas Rojas A, Martín Herrera L. Risk factors for hepatocellular carcinoma in patients with liver cirrhosis. Rev Esp Enferm Dig. 2000; 92(7): 458-69.	Liver cancer due to hepatitis B
Spain	1990	1994	Macías Rodríguez MA, Rendón Unceta P, Tejada Cabrera M, Infante Hernández JM, Correro Aguilar F, Díaz García F, Benítez Rodríguez E, Mangas Rojas A, Martín Herrera L. Risk factors for hepatocellular carcinoma in patients with liver cirrhosis. Rev Esp Enferm Dig. 2000; 92(7): 458-69.	Liver cancer due to hepatitis C
Sudan	1996	1998	Omer RE, Van't Veer P, Kadaru AM, Kampman E, el Khidir IM, Fedail SS, Kok FJ. The role of hepatitis B and hepatitis C viral infections in the incidence of hepatocellular carcinoma in Sudan. Trans R Soc Trop Med Hyg. 2001; 95(5): 487-91.	Liver cancer due to hepatitis B
Sudan	1996	1998	Omer RE, Van't Veer P, Kadaru AM, Kampman E, el Khidir IM, Fedail SS, Kok FJ. The role of	Liver cancer

©2017 Global Burden of Disease Liver Cancer Collaboration. JAMA Oncology.

Country	Years included in estimation (start, end)		Citation	Liver cancer etiology
			hepatitis B and hepatitis C viral infections in the incidence of hepatocellular carcinoma in Sudan. Trans R Soc Trop Med Hyg. 2001; 95(5): 487-91.	due to hepatitis C
Taiwan	1996	1999	Lee CM, Lu SN, Changchien CS, Yeh CT, Hsu TT, Tang JH, Wang JH, Lin DY, Chen CL, Chen WJ. Age, gender, and local geographic variations of viral etiology of hepatocellular carcinoma in a hyperendemic area for hepatitis B virus infection. Cancer. 1999; 86(7): 1143-50.	Liver cancer due to hepatitis B
Taiwan	1996	1999	Lee CM, Lu SN, Changchien CS, Yeh CT, Hsu TT, Tang JH, Wang JH, Lin DY, Chen CL, Chen WJ. Age, gender, and local geographic variations of viral etiology of hepatocellular carcinoma in a hyperendemic area for hepatitis B virus infection. Cancer. 1999; 86(7): 1143-50.	Liver cancer due to hepatitis C
Thailand	2007	2012	Somboon K, Siramolpiwat S, Vilaichone RK. Epidemiology and survival of hepatocellular carcinoma in the central region of Thailand. Asian Pac J Cancer Prev. 2014; 15(8): 3567-70.	Liver cancer due to alcohol use
Thailand	2007	2012	Somboon K, Siramolpiwat S, Vilaichone RK. Epidemiology and survival of hepatocellular carcinoma in the central region of Thailand. Asian Pac J Cancer Prev. 2014; 15(8): 3567-70.	Liver cancer due to hepatitis B
Thailand	2007	2012	Somboon K, Siramolpiwat S, Vilaichone RK. Epidemiology and survival of hepatocellular carcinoma in the central region of Thailand. Asian Pac J Cancer Prev. 2014; 15(8): 3567-70.	Liver cancer due to hepatitis C
Thailand	2007	2012	Somboon K, Siramolpiwat S, Vilaichone RK. Epidemiology and survival of hepatocellular carcinoma in the central region of Thailand. Asian Pac J Cancer Prev. 2014; 15(8): 3567-70.	Liver cancer due to other causes
Thailand	1997	1998	Tangkijvanich P, Hirsch P, Theamboonlers A, Nuchprayoon I, Poovorawan Y. Association of hepatitis viruses with hepatocellular carcinoma in Thailand. J Gastroenterol. 1999; 34(2): 227-33.	Liver cancer due to hepatitis B
Thailand	1997	1998	Tangkijvanich P, Hirsch P, Theamboonlers A, Nuchprayoon I, Poovorawan Y. Association of hepatitis viruses with hepatocellular carcinoma in Thailand. J Gastroenterol. 1999; 34(2): 227-33.	Liver cancer due to hepatitis C
The Gambia	1997	2001	Kirk GD, Lesi OA, Mendy M, Akano AO, Sam O, Goedert JJ, Hainaut P, Hall AJ, Whittle H, Montesano R. The Gambia Liver Cancer Study: Infection with hepatitis B and C and the risk of hepatocellular carcinoma in West Africa. Hepatology. 2004; 39(1): 211-9.	Liver cancer due to hepatitis B
The Gambia	1997	2001	Kirk GD, Lesi OA, Mendy M, Akano AO, Sam O, Goedert JJ, Hainaut P, Hall AJ, Whittle H, Montesano R. The Gambia Liver Cancer Study: Infection with hepatitis B and C and the risk of	Liver cancer due to

©2017 Global Burden of Disease Liver Cancer Collaboration. JAMA Oncology.

Country	Years included in estimation (start, end)		Citation	Liver cancer etiology
			hepatocellular carcinoma in West Africa. Hepatology. 2004; 39(1): 211-9.	hepatitis C
The Gambia	2002	2002	Mboto CI, Davies-Russell A, Fielder M, Jewell AP. Hepatocellular Carcinoma in The Gambia and the role of Hepatitis B and Hepatitis C. Int Semin Surg Oncol. 2005; 2: 20.	Liver cancer due to hepatitis B
The Gambia	2002	2002	Mboto CI, Davies-Russell A, Fielder M, Jewell AP. Hepatocellular Carcinoma in The Gambia and the role of Hepatitis B and Hepatitis C. Int Semin Surg Oncol. 2005; 2: 20.	Liver cancer due to hepatitis C
The Gambia	1997	2001	Umoh NJ, Lesi OA, Mendy M, Bah E, Akano A, Whittle H, Hainaut P, Kirk GD. Aetiological differences in demographical, clinical and pathological characteristics of hepatocellular carcinoma in The Gambia. Liver Int. 2011; 31(2): 215-21.	Liver cancer due to hepatitis B
The Gambia	1997	2001	Umoh NJ, Lesi OA, Mendy M, Bah E, Akano A, Whittle H, Hainaut P, Kirk GD. Aetiological differences in demographical, clinical and pathological characteristics of hepatocellular carcinoma in The Gambia. Liver Int. 2011; 31(2): 215-21.	Liver cancer due to hepatitis C
Tunisia	1997	2009	Said Y, Debbeche R, Ben Ali Z, Bouzid K, Trabelsi S, Bouzaidi S, Salem M, Rajhi H, Kouni Chahed M, Najjar T. [Epidemiological, clinical and therapeutic features of hepatocellular carcinoma in cirrhotic patients]. Tunis Med. 2012; 90(6): 468-72.	Liver cancer due to alcohol use
Tunisia	1997	2009	Said Y, Debbeche R, Ben Ali Z, Bouzid K, Trabelsi S, Bouzaidi S, Salem M, Rajhi H, Kouni Chahed M, Najjar T. [Epidemiological, clinical and therapeutic features of hepatocellular carcinoma in cirrhotic patients]. Tunis Med. 2012; 90(6): 468-72.	Liver cancer due to hepatitis B
Tunisia	1997	2009	Said Y, Debbeche R, Ben Ali Z, Bouzid K, Trabelsi S, Bouzaidi S, Salem M, Rajhi H, Kouni Chahed M, Najjar T. [Epidemiological, clinical and therapeutic features of hepatocellular carcinoma in cirrhotic patients]. Tunis Med. 2012; 90(6): 468-72.	Liver cancer due to hepatitis C
Tunisia	1997	2009	Said Y, Debbeche R, Ben Ali Z, Bouzid K, Trabelsi S, Bouzaidi S, Salem M, Rajhi H, Kouni Chahed M, Najjar T. [Epidemiological, clinical and therapeutic features of hepatocellular carcinoma in cirrhotic patients]. Tunis Med. 2012; 90(6): 468-72.	Liver cancer due to other causes
Tunisia	1991	1994	Triki H. [Epidemiology of hepatitis B virus, hepatitis C virus and Delta virus in the general population and in liver cirrhosis in Tunisia]. Arch Inst Pasteur Tunis. 1994; 71(3-4): 403-6.	Liver cancer due to hepatitis B
Tunisia	1991	1994	Triki H. [Epidemiology of hepatitis B virus, hepatitis C virus and Delta virus in the general population and in liver cirrhosis in Tunisia]. Arch Inst Pasteur Tunis. 1994; 71(3-4): 403-6.	Liver cancer due to hepatitis C

©2017 Global Burden of Disease Liver Cancer Collaboration. JAMA Oncology.

Country	Years included in estimation (start, end)		Citation	Liver cancer etiology
Turkey	1994	2007	Alacacioglu A, Somali I, Simsek I, Astarcioglu I, Ozkan M, Camci C, Alkis N, Karaoglu A, Tarhan O, Unek T, Yilmaz U. Epidemiology and survival of hepatocellular carcinoma in Turkey: outcome of multicenter study. Jpn J Clin Oncol. 2008; 38(10): 683-8.	Liver cancer due to alcohol use
Turkey	1994	2007	Alacacioglu A, Somali I, Simsek I, Astarcioglu I, Ozkan M, Camci C, Alkis N, Karaoglu A, Tarhan O, Unek T, Yilmaz U. Epidemiology and survival of hepatocellular carcinoma in Turkey: outcome of multicenter study. Jpn J Clin Oncol. 2008; 38(10): 683-8.	Liver cancer due to hepatitis B
Turkey	1994	2007	Alacacioglu A, Somali I, Simsek I, Astarcioglu I, Ozkan M, Camci C, Alkis N, Karaoglu A, Tarhan O, Unek T, Yilmaz U. Epidemiology and survival of hepatocellular carcinoma in Turkey: outcome of multicenter study. Jpn J Clin Oncol. 2008; 38(10): 683-8.	Liver cancer due to hepatitis C
Turkey	1994	2007	Alacacioglu A, Somali I, Simsek I, Astarcioglu I, Ozkan M, Camci C, Alkis N, Karaoglu A, Tarhan O, Unek T, Yilmaz U. Epidemiology and survival of hepatocellular carcinoma in Turkey: outcome of multicenter study. Jpn J Clin Oncol. 2008; 38(10): 683-8.	Liver cancer due to other causes
Turkey	1999	2002	Ozer B, Serin E, Yilmaz U, Gümürdülü Y, Saygili OB, Kayaselçuk F, Boyacioğlu S. Clinicopathologic features and risk factors for hepatocellular carcinoma: results from a single center in southern Turkey. Turk J Gastroenterol. 2003; 14(2): 85-90.	Liver cancer due to alcohol use
Turkey	1999	2002	Ozer B, Serin E, Yilmaz U, Gümürdülü Y, Saygili OB, Kayaselçuk F, Boyacioğlu S. Clinicopathologic features and risk factors for hepatocellular carcinoma: results from a single center in southern Turkey. Turk J Gastroenterol. 2003; 14(2): 85-90.	Liver cancer due to hepatitis B
Turkey	1999	2002	Ozer B, Serin E, Yilmaz U, Gümürdülü Y, Saygili OB, Kayaselçuk F, Boyacioğlu S. Clinicopathologic features and risk factors for hepatocellular carcinoma: results from a single center in southern Turkey. Turk J Gastroenterol. 2003; 14(2): 85-90.	Liver cancer due to hepatitis C
Turkey	1999	2002	Ozer B, Serin E, Yilmaz U, Gümürdülü Y, Saygili OB, Kayaselçuk F, Boyacioğlu S. Clinicopathologic features and risk factors for hepatocellular carcinoma: results from a single center in southern Turkey. Turk J Gastroenterol. 2003; 14(2): 85-90.	Liver cancer due to other causes
Turkey	1995	1997	Ozyilkan O, Arslan M, Ozyilkan E. Hepatitis B virus and hepatitis C virus infections in Turkish patients with hepatocellular carcinoma. Am J Gastroenterol. 1996; 91(7): 1479-80.	Liver cancer due to hepatitis B
Turkey	1995	1997	Ozyilkan O, Arslan M, Ozyilkan E. Hepatitis B virus and hepatitis C virus infections in Turkish patients with hepatocellular carcinoma. Am J Gastroenterol. 1996; 91(7): 1479-80.	Liver cancer due to hepatitis C
Turkey	1994	1997	Uzunalimoğlu O, Yurdaydin C, Cetinkaya H, Bozkaya H, Sahin T, Colakoğlu S, Tankurt E, Sarioğlu	Liver cancer

©2017 Global Burden of Disease Liver Cancer Collaboration. JAMA Oncology.

Country	Years included in estimation (start, end)		Citation	Liver cancer etiology
			M, Ozenirler S, Akkiz H, Tözün N, Değertekin H, Okten A. Risk factors for hepatocellular carcinoma in Turkey. Dig Dis Sci. 2001; 46(5): 1022-8.	due to alcohol use
Turkey	1994	1997	Uzunalimoğlu O, Yurdaydin C, Cetinkaya H, Bozkaya H, Sahin T, Colakoğlu S, Tankurt E, Sarioğlu M, Ozenirler S, Akkiz H, Tözün N, Değertekin H, Okten A. Risk factors for hepatocellular carcinoma in Turkey. Dig Dis Sci. 2001; 46(5): 1022-8.	Liver cancer due to hepatitis B
Turkey	1994	1997	Uzunalimoğlu O, Yurdaydin C, Cetinkaya H, Bozkaya H, Sahin T, Colakoğlu S, Tankurt E, Sarioğlu M, Ozenirler S, Akkiz H, Tözün N, Değertekin H, Okten A. Risk factors for hepatocellular carcinoma in Turkey. Dig Dis Sci. 2001; 46(5): 1022-8.	Liver cancer due to hepatitis C
Turkey	1994	1997	Uzunalimoğlu O, Yurdaydin C, Cetinkaya H, Bozkaya H, Sahin T, Colakoğlu S, Tankurt E, Sarioğlu M, Ozenirler S, Akkiz H, Tözün N, Değertekin H, Okten A. Risk factors for hepatocellular carcinoma in Turkey. Dig Dis Sci. 2001; 46(5): 1022-8.	Liver cancer due to other causes
United States	1993	1999	Davila JA, Morgan RO, Shaib Y, McGlynn KA, El-Serag HB. Hepatitis C infection and the increasing incidence of hepatocellular carcinoma: a population-based study. Gastroenterology. 2004; 127(5): 1372-80.	Liver cancer due to alcohol use
United States	1993	1999	Davila JA, Morgan RO, Shaib Y, McGlynn KA, El-Serag HB. Hepatitis C infection and the increasing incidence of hepatocellular carcinoma: a population-based study. Gastroenterology. 2004; 127(5): 1372-80.	Liver cancer due to hepatitis B
United States	1993	1999	Davila JA, Morgan RO, Shaib Y, McGlynn KA, El-Serag HB. Hepatitis C infection and the increasing incidence of hepatocellular carcinoma: a population-based study. Gastroenterology. 2004; 127(5): 1372-80.	Liver cancer due to hepatitis C
United States	1993	1999	Davila JA, Morgan RO, Shaib Y, McGlynn KA, El-Serag HB. Hepatitis C infection and the increasing incidence of hepatocellular carcinoma: a population-based study. Gastroenterology. 2004; 127(5): 1372-80.	Liver cancer due to other causes
United States	1997	1999	Di Bisceglie AM, Lyra AC, Schwartz M, Reddy RK, Martin P, Gores G, Lok ASF, Hussain KB, Gish R, Van Thiel DH, Younossi Z, Tong M, Hassanein T, Balart L, Fleckenstein J, Flamm S, Blei A, Befeler AS, Liver Cancer Network. Hepatitis C-related hepatocellular carcinoma in the United States: influence of ethnic status. Am J Gastroenterol. 2003; 98(9): 2060-3.	Liver cancer due to hepatitis B
United States	1997	1999	Di Bisceglie AM, Lyra AC, Schwartz M, Reddy RK, Martin P, Gores G, Lok ASF, Hussain KB, Gish R, Van Thiel DH, Younossi Z, Tong M, Hassanein T, Balart L, Fleckenstein J, Flamm S, Blei A, Befeler AS, Liver Cancer Network. Hepatitis C-related hepatocellular carcinoma in the United States: influence of ethnic status. Am J Gastroenterol. 2003; 98(9): 2060-3.	Liver cancer due to hepatitis C

©2017 Global Burden of Disease Liver Cancer Collaboration. JAMA Oncology.

Country	Years included in estimation (start, end)		Citation	Liver cancer etiology
United States	2001	2009	Ha NB, Ha NB, Ahmed A, Ayoub W, Daugherty TJ, Chang ET, Lutchman GA, Garcia G, Cooper AD, Keeffe EB, Nguyen MH. Risk factors for hepatocellular carcinoma in patients with chronic liver disease: a case-control study. Cancer Causes Control. 2012; 23(3): 455-62.	Liver cancer due to hepatitis B
United States	2001	2009	Ha NB, Ha NB, Ahmed A, Ayoub W, Daugherty TJ, Chang ET, Lutchman GA, Garcia G, Cooper AD, Keeffe EB, Nguyen MH. Risk factors for hepatocellular carcinoma in patients with chronic liver disease: a case-control study. Cancer Causes Control. 2012; 23(3): 455-62.	Liver cancer due to hepatitis C
United States	1993	1995	Hassan MM, Frome A, Patt YZ, El-Serag HB. Rising prevalence of hepatitis C virus infection among patients recently diagnosed with hepatocellular carcinoma in the United States. J Clin Gastroenterol. 2002; 35(3): 266-9.	Liver cancer due to hepatitis B
United States	1993	1995	Hassan MM, Frome A, Patt YZ, El-Serag HB. Rising prevalence of hepatitis C virus infection among patients recently diagnosed with hepatocellular carcinoma in the United States. J Clin Gastroenterol. 2002; 35(3): 266-9.	Liver cancer due to hepatitis C
United States	2002	2003	Marrero JA, Fontana RJ, Fu S, Conjeevaram HS, Su GL, Lok AS. Alcohol, tobacco and obesity are synergistic risk factors for hepatocellular carcinoma. J Hepatol. 2005; 42(2): 218-24.	Liver cancer due to alcohol use
United States	2002	2003	Marrero JA, Fontana RJ, Fu S, Conjeevaram HS, Su GL, Lok AS. Alcohol, tobacco and obesity are synergistic risk factors for hepatocellular carcinoma. J Hepatol. 2005; 42(2): 218-24.	Liver cancer due to hepatitis B
United States	2002	2003	Marrero JA, Fontana RJ, Fu S, Conjeevaram HS, Su GL, Lok AS. Alcohol, tobacco and obesity are synergistic risk factors for hepatocellular carcinoma. J Hepatol. 2005; 42(2): 218-24.	Liver cancer due to hepatitis C
United States	2002	2003	Marrero JA, Fontana RJ, Fu S, Conjeevaram HS, Su GL, Lok AS. Alcohol, tobacco and obesity are synergistic risk factors for hepatocellular carcinoma. J Hepatol. 2005; 42(2): 218-24.	Liver cancer due to other causes
United States	1994	2010	Welzel TM, Graubard BI, Quraishi S, Zeuzem S, Davila JA, El-Serag HB, McGlynn KA. Population-attributable fractions of risk factors for hepatocellular carcinoma in the United States. Am J Gastroenterol. 2013; 108(8): 1314-21.	Liver cancer due to alcohol use
United States	1994	2010	Welzel TM, Graubard BI, Quraishi S, Zeuzem S, Davila JA, El-Serag HB, McGlynn KA. Population-attributable fractions of risk factors for hepatocellular carcinoma in the United States. Am J Gastroenterol. 2013; 108(8): 1314-21.	Liver cancer due to hepatitis B
United	1994	2010	Welzel TM, Graubard BI, Quraishi S, Zeuzem S, Davila JA, El-Serag HB, McGlynn KA. Population-	Liver cancer

©2017 Global Burden of Disease Liver Cancer Collaboration. JAMA Oncology.

Country	Years included in estimation (start, end)		Citation	Liver cancer etiology
States			attributable fractions of risk factors for hepatocellular carcinoma in the United States. Am J Gastroenterol. 2013; 108(8): 1314-21.	due to hepatitis C
United States	1994	2010	Welzel TM, Graubard BI, Quraishi S, Zeuzem S, Davila JA, El-Serag HB, McGlynn KA. Population-attributable fractions of risk factors for hepatocellular carcinoma in the United States. Am J Gastroenterol. 2013; 108(8): 1314-21.	Liver cancer due to other causes
United States	2007	2009	Yang JD, Harmsen WS, Slettedahl SW, Chaiteerakij R, Enders FT, Therneau TM, Orsini L, Kim WR, Roberts LR. Factors that affect risk for hepatocellular carcinoma and effects of surveillance. Clin Gastroenterol Hepatol. 2011; 9(7): 617-623.	Liver cancer due to alcohol use
United States	2007	2009	Yang JD, Harmsen WS, Slettedahl SW, Chaiteerakij R, Enders FT, Therneau TM, Orsini L, Kim WR, Roberts LR. Factors that affect risk for hepatocellular carcinoma and effects of surveillance. Clin Gastroenterol Hepatol. 2011; 9(7): 617-623.	Liver cancer due to other causes
United States	1976	1990	Yang JD, Kim B, Sanderson SO, St Sauver JL, Yawn BP, Pedersen RA, Larson JJ, Therneau TM, Roberts LR, Kim WR. Hepatocellular carcinoma in olmsted county, Minnesota, 1976-2008. Mayo Clin Proc. 2012; 87(1): 9-16.	Liver cancer due to hepatitis B
United States	1976	1990	Yang JD, Kim B, Sanderson SO, St Sauver JL, Yawn BP, Pedersen RA, Larson JJ, Therneau TM, Roberts LR, Kim WR. Hepatocellular carcinoma in olmsted county, Minnesota, 1976-2008. Mayo Clin Proc. 2012; 87(1): 9-16.	Liver cancer due to hepatitis C
United States	1991	2000	Yang JD, Kim B, Sanderson SO, St Sauver JL, Yawn BP, Pedersen RA, Larson JJ, Therneau TM, Roberts LR, Kim WR. Hepatocellular carcinoma in olmsted county, Minnesota, 1976-2008. Mayo Clin Proc. 2012; 87(1): 9-16.	Liver cancer due to hepatitis B
United States	1991	2000	Yang JD, Kim B, Sanderson SO, St Sauver JL, Yawn BP, Pedersen RA, Larson JJ, Therneau TM, Roberts LR, Kim WR. Hepatocellular carcinoma in olmsted county, Minnesota, 1976-2008. Mayo Clin Proc. 2012; 87(1): 9-16.	Liver cancer due to hepatitis C
United States	2011	2008	Yang JD, Kim B, Sanderson SO, St Sauver JL, Yawn BP, Pedersen RA, Larson JJ, Therneau TM, Roberts LR, Kim WR. Hepatocellular carcinoma in olmsted county, Minnesota, 1976-2008. Mayo Clin Proc. 2012; 87(1): 9-16.	Liver cancer due to hepatitis B
United States	2011	2008	Yang JD, Kim B, Sanderson SO, St Sauver JL, Yawn BP, Pedersen RA, Larson JJ, Therneau TM, Roberts LR, Kim WR. Hepatocellular carcinoma in olmsted county, Minnesota, 1976-2008. Mayo Clin Proc. 2012; 87(1): 9-16.	Liver cancer due to hepatitis C
United States	2000	2000	Yu L, Sloane DA, Guo C, Howell CD. Risk factors for primary hepatocellular carcinoma in black and white Americans in 2000. Clin Gastroenterol Hepatol. 2006; 4(3): 355-60.	Liver cancer due to alcohol

©2017 Global Burden of Disease Liver Cancer Collaboration. JAMA Oncology.

Country	Years included in estimation (start, end)		Citation	Liver cancer etiology
				use
United States	2000	2000	Yu L, Sloane DA, Guo C, Howell CD. Risk factors for primary hepatocellular carcinoma in black and white Americans in 2000. Clin Gastroenterol Hepatol. 2006; 4(3): 355-60.	Liver cancer due to hepatitis B
United States	2000	2000	Yu L, Sloane DA, Guo C, Howell CD. Risk factors for primary hepatocellular carcinoma in black and white Americans in 2000. Clin Gastroenterol Hepatol. 2006; 4(3): 355-60.	Liver cancer due to hepatitis C
United States	2000	2000	Yu L, Sloane DA, Guo C, Howell CD. Risk factors for primary hepatocellular carcinoma in black and white Americans in 2000. Clin Gastroenterol Hepatol. 2006; 4(3): 355-60.	Liver cancer due to other causes
Yemen	2001	2008	Salem AK, Abdulrab A, Alfakeh Y, Aown A. Hepatocellular carcinoma in Yemeni patients: a single centre experience over an 8-year period. East Mediterr Health J. 2012; 18(7): 693-9.	Liver cancer due to alcohol use
Yemen	2001	2008	Salem AK, Abdulrab A, Alfakeh Y, Aown A. Hepatocellular carcinoma in Yemeni patients: a single centre experience over an 8-year period. East Mediterr Health J. 2012; 18(7): 693-9.	Liver cancer due to hepatitis B
Yemen	2001	2008	Salem AK, Abdulrab A, Alfakeh Y, Aown A. Hepatocellular carcinoma in Yemeni patients: a single centre experience over an 8-year period. East Mediterr Health J. 2012; 18(7): 693-9.	Liver cancer due to hepatitis C
Yemen	2001	2008	Salem AK, Abdulrab A, Alfakeh Y, Aown A. Hepatocellular carcinoma in Yemeni patients: a single centre experience over an 8-year period. East Mediterr Health J. 2012; 18(7): 693-9.	Liver cancer due to other causes
Zimbabwe	1994	1996	Weinig M, Hakim JG, Gudza I, Tobaiwa O. Hepatitis C virus and HIV antibodies in patients with hepatocellular carcinoma in Zimbabwe: a pilot study. Trans R Soc Trop Med Hyg. 1997; 91(5): 570-2.	Liver cancer due to hepatitis B
Zimbabwe	1994	1996	Weinig M, Hakim JG, Gudza I, Tobaiwa O. Hepatitis C virus and HIV antibodies in patients with hepatocellular carcinoma in Zimbabwe: a pilot study. Trans R Soc Trop Med Hyg. 1997; 91(5): 570-2.	Liver cancer due to hepatitis C

©2017 Global Burden of Disease Liver Cancer Collaboration. JAMA Oncology.

eTable 6: Disability weights including uncertainty intervals

Health state	Lay descriptions used to derive disability weights[23]	Estimate	Uncertainty interval	
Cancer, diagnosis and primary therapy	Has pain, nausea, fatigue, weight loss and high anxiety.	0.288	0.193	0.399
Cancer, controlled phase	Has a chronic disease that requires medication every day and causes some worry but minimal interference with daily activities.	0.049	0.031	0.072
Cancer, metastatic	Has severe pain, extreme fatigue, weight loss and high anxiety.	0.451	0.307	0.600
Terminal phase	Has lost a lot of weight and regularly uses strong medication to avoid constant pain. The person has no appetite, feels nauseous, and needs to spend most of the day in bed.	0.540	0.377	0.687

©2017 Global Burden of Disease Liver Cancer Collaboration. JAMA Oncology.

eTable 7: Socio-demographic Index groupings by geography, based on 2015 values

Location	SDI level
Aichi	High SDI
Akita	High SDI
Alabama	High SDI
Alaska	High SDI
Andorra	High SDI
Antigua and Barbuda	High SDI
Aomori	High SDI
Arizona	High SDI
Arkansas	High SDI
Australia	High SDI
Austria	High SDI
Beijing	High SDI
Belarus	High SDI
Belgium	High SDI
Bermuda	High SDI
Brunei	High SDI
California	High SDI
Canada	High SDI
Chiba	High SDI
Colorado	High SDI
Connecticut	High SDI
Cyprus	High SDI
Czech Republic	High SDI
Delaware	High SDI
Denmark	High SDI
District of Columbia	High SDI
Distrito Federal	High SDI
Distrito Federal	High SDI
East Midlands	High SDI
East of England	High SDI
Ehime	High SDI
Estonia	High SDI
Finland	High SDI
Florida	High SDI
France	High SDI
Fukui	High SDI
Fukuoka	High SDI
Fukushima	High SDI
Georgia	High SDI

©2017 Global Burden of Disease Liver Cancer Collaboration. JAMA Oncology.

Location	SDI level
Germany	High SDI
Gifu	High SDI
Greater London	High SDI
Guam	High SDI
Gunma	High SDI
Hawaii	High SDI
Hiroshima	High SDI
Hokkaidō	High SDI
Hong Kong Special Administrative Region of China	High SDI
Hungary	High SDI
Hyōgo	High SDI
Ibaraki	High SDI
Iceland	High SDI
Idaho	High SDI
Illinois	High SDI
Indiana	High SDI
Iowa	High SDI
Ireland	High SDI
Ishikawa	High SDI
Israel	High SDI
Italy	High SDI
Iwate	High SDI
Kagawa	High SDI
Kagoshima	High SDI
Kanagawa	High SDI
Kansas	High SDI
Kentucky	High SDI
Kōchi	High SDI
Kumamoto	High SDI
Kuwait	High SDI
Kyōto	High SDI
Latvia	High SDI
Lithuania	High SDI
Louisiana	High SDI
Luxembourg	High SDI
Macao Special Administrative Region of China	High SDI
Maine	High SDI
Maryland	High SDI
Massachusetts	High SDI

©2017 Global Burden of Disease Liver Cancer Collaboration. JAMA Oncology.

Location	SDI level
Michigan	High SDI
Mie	High SDI
Minnesota	High SDI
Mississippi	High SDI
Missouri	High SDI
Miyagi	High SDI
Miyazaki	High SDI
Montana	High SDI
Nagano	High SDI
Nagasaki	High SDI
Nara	High SDI
Nebraska	High SDI
Netherlands	High SDI
Nevada	High SDI
New Hampshire	High SDI
New Jersey	High SDI
New Mexico	High SDI
New York	High SDI
New Zealand	High SDI
Niigata	High SDI
North Carolina	High SDI
North Dakota	High SDI
North West England	High SDI
Northern Mariana Islands	High SDI
Norway	High SDI
Ohio	High SDI
Ôita	High SDI
Okayama	High SDI
Okinawa	High SDI
Oklahoma	High SDI
Oregon	High SDI
Ōsaka	High SDI
Pennsylvania	High SDI
Poland	High SDI
Puerto Rico	High SDI
Rhode Island	High SDI
Russia	High SDI
Saga	High SDI
Saitama	High SDI
Scotland	High SDI

©2017 Global Burden of Disease Liver Cancer Collaboration. JAMA Oncology.

Location	SDI level
Shanghai	High SDI
Shiga	High SDI
Shimane	High SDI
Shizuoka	High SDI
Singapore	High SDI
Slovakia	High SDI
Slovenia	High SDI
South Carolina	High SDI
South Dakota	High SDI
South East England	High SDI
South Korea	High SDI
South West England	High SDI
Stockholm	High SDI
Sweden except Stockholm	High SDI
Switzerland	High SDI
Taiwan	High SDI
Tennessee	High SDI
Texas	High SDI
The Bahamas	High SDI
Tianjin	High SDI
Tochigi	High SDI
Tokushima	High SDI
Tōkyō	High SDI
Tottori	High SDI
Toyama	High SDI
Trinidad and Tobago	High SDI
United Arab Emirates	High SDI
Utah	High SDI
Vermont	High SDI
Virgin Islands, U.S.	High SDI
Virginia	High SDI
Wakayama	High SDI
Wales	High SDI
Washington	High SDI
West Midlands	High SDI
West Virginia	High SDI
Wisconsin	High SDI
Wyoming	High SDI
Yamagata	High SDI
Yamaguchi	High SDI

©2017 Global Burden of Disease Liver Cancer Collaboration. JAMA Oncology.

Location	SDI level
Yamanashi	High SDI
Yorkshire and the Humber	High SDI
'Asir	High-middle SDI
Aguascalientes	High-middle SDI
Albania	High-middle SDI
American Samoa	High-middle SDI
Andhra Pradesh, Urban	High-middle SDI
Argentina	High-middle SDI
Armenia	High-middle SDI
Azerbaijan	High-middle SDI
Bahah	High-middle SDI
Bahrain	High-middle SDI
Baja California	High-middle SDI
Baja California Sur	High-middle SDI
Barbados	High-middle SDI
Bosnia and Herzegovina	High-middle SDI
Bulgaria	High-middle SDI
Campeche	High-middle SDI
Chihuahua	High-middle SDI
Chile	High-middle SDI
Coahuila	High-middle SDI
Colima	High-middle SDI
Colombia	High-middle SDI
Costa Rica	High-middle SDI
Croatia	High-middle SDI
Cuba	High-middle SDI
Delhi, Rural	High-middle SDI
Delhi, Urban	High-middle SDI
Dominica	High-middle SDI
Dominican Republic	High-middle SDI
Durango	High-middle SDI
Eastern Cape	High-middle SDI
Eastern Province	High-middle SDI
Ecuador	High-middle SDI
Espírito Santo	High-middle SDI
Fiji	High-middle SDI
Free State	High-middle SDI
Gauteng	High-middle SDI
Georgia	High-middle SDI
Goa, Rural	High-middle SDI

©2017 Global Burden of Disease Liver Cancer Collaboration. JAMA Oncology.

Location	SDI level
Goa, Urban	High-middle SDI
Greece	High-middle SDI
Greenland	High-middle SDI
Grenada	High-middle SDI
Guangdong	High-middle SDI
Ha'il	High-middle SDI
Haryāna, Urban	High-middle SDI
Heilongjiang	High-middle SDI
Himachal Pradesh, Urban	High-middle SDI
Inner Mongolia	High-middle SDI
Iran	High-middle SDI
Jalisco	High-middle SDI
Jamaica	High-middle SDI
Jawf	High-middle SDI
Jiangsu	High-middle SDI
Jilin	High-middle SDI
Jordan	High-middle SDI
Karnātaka, Urban	High-middle SDI
Kazakhstan	High-middle SDI
KwaZulu-Natal	High-middle SDI
Lebanon	High-middle SDI
Liaoning	High-middle SDI
Macedonia	High-middle SDI
Madinah	High-middle SDI
Mahārāshtra, Urban	High-middle SDI
Makkah	High-middle SDI
Malaysia	High-middle SDI
Malta	High-middle SDI
Mauritius	High-middle SDI
México	High-middle SDI
Moldova	High-middle SDI
Mongolia	High-middle SDI
Montenegro	High-middle SDI
Morelos	High-middle SDI
Mpumalanga	High-middle SDI
Nairobi	High-middle SDI
Nayarit	High-middle SDI
North East England	High-middle SDI
North-West	High-middle SDI
Northern Borders	High-middle SDI

©2017 Global Burden of Disease Liver Cancer Collaboration. JAMA Oncology.

Location	SDI level
Northern Cape	High-middle SDI
Northern Ireland	High-middle SDI
Nuevo León	High-middle SDI
Oman	High-middle SDI
Panama	High-middle SDI
Peru	High-middle SDI
Portugal	High-middle SDI
Punjab, Urban	High-middle SDI
Qassim	High-middle SDI
Qatar	High-middle SDI
Querétaro	High-middle SDI
Quintana Roo	High-middle SDI
Rio de Janeiro	High-middle SDI
Rio Grande do Sul	High-middle SDI
Riyadh	High-middle SDI
Romania	High-middle SDI
Saint Lucia	High-middle SDI
Saint Vincent and the Grenadines	High-middle SDI
San Luis Potosí	High-middle SDI
Santa Catarina	High-middle SDI
São Paulo	High-middle SDI
Serbia	High-middle SDI
Seychelles	High-middle SDI
Shandong	High-middle SDI
Shanxi	High-middle SDI
Sikkim, Urban	High-middle SDI
Sinaloa	High-middle SDI
Sonora	High-middle SDI
Spain	High-middle SDI
Sri Lanka	High-middle SDI
Suriname	High-middle SDI
Tabasco	High-middle SDI
Tabuk	High-middle SDI
Tamaulipas	High-middle SDI
Tamil Nādu, Urban	High-middle SDI
Thailand	High-middle SDI
The Six Minor Territories, Urban	High-middle SDI
Tlaxcala	High-middle SDI
Turkey	High-middle SDI
Turkmenistan	High-middle SDI

©2017 Global Burden of Disease Liver Cancer Collaboration. JAMA Oncology.

Location	SDI level
Ukraine	High-middle SDI
Uruguay	High-middle SDI
Uttarakhand, Urban	High-middle SDI
Uzbekistan	High-middle SDI
Venezuela	High-middle SDI
Western Cape	High-middle SDI
Yucatán	High-middle SDI
Zhejiang	High-middle SDI
Acre	Middle SDI
Algeria	Middle SDI
Amapá	Middle SDI
Amazonas	Middle SDI
Andhra Pradesh, Rural	Middle SDI
Anhui	Middle SDI
Arunāchal Pradesh, Urban	Middle SDI
Assam, Urban	Middle SDI
Bahia	Middle SDI
Belize	Middle SDI
Bihār, Urban	Middle SDI
Bolivia	Middle SDI
Botswana	Middle SDI
Chhattīsgarh, Urban	Middle SDI
Chiapas	Middle SDI
Chongqing	Middle SDI
Egypt	Middle SDI
El Salvador	Middle SDI
Equatorial Guinea	Middle SDI
Federated States of Micronesia	Middle SDI
Fujian	Middle SDI
Gabon	Middle SDI
Gansu	Middle SDI
Goiás	Middle SDI
Guanajuato	Middle SDI
Guangxi	Middle SDI
Guerrero	Middle SDI
Gujarāt, Urban	Middle SDI
Guyana	Middle SDI
Hainan	Middle SDI
Haryāna, Rural	Middle SDI
Hebei	Middle SDI

©2017 Global Burden of Disease Liver Cancer Collaboration. JAMA Oncology.

Location	SDI level
Henan	Middle SDI
Hidalgo	Middle SDI
Himachal Pradesh, Rural	Middle SDI
Honduras	Middle SDI
Hubei	Middle SDI
Hunan	Middle SDI
Indonesia	Middle SDI
Iraq	Middle SDI
Jammu and Kashmīr, Urban	Middle SDI
Jharkhand, Urban	Middle SDI
Jiangxi	Middle SDI
Jizan	Middle SDI
Kerala, Rural	Middle SDI
Kerala, Urban	Middle SDI
Kiambu	Middle SDI
Kyrgyzstan	Middle SDI
Laikipia	Middle SDI
Libya	Middle SDI
Limpopo	Middle SDI
Madhya Pradesh, Urban	Middle SDI
Mahārāshtra, Rural	Middle SDI
Maldives	Middle SDI
Manipur, Urban	Middle SDI
Marshall Islands	Middle SDI
Mato Grosso	Middle SDI
Mato Grosso do Sul	Middle SDI
Meghālaya, Urban	Middle SDI
Michoacán de Ocampo	Middle SDI
Minas Gerais	Middle SDI
Mizoram, Urban	Middle SDI
Mombasa	Middle SDI
Nāgāland, Rural	Middle SDI
Nāgāland, Urban	Middle SDI
Najran	Middle SDI
Namibia	Middle SDI
Nicaragua	Middle SDI
Ningxia	Middle SDI
North Korea	Middle SDI
Nyeri	Middle SDI
Oaxaca	Middle SDI

©2017 Global Burden of Disease Liver Cancer Collaboration. JAMA Oncology.

Location	SDI level
Orissa, Urban	Middle SDI
Palestine	Middle SDI
Pará	Middle SDI
Paraguay	Middle SDI
Paraná	Middle SDI
Pernambuco	Middle SDI
Philippines	Middle SDI
Puebla	Middle SDI
Punjab, Rural	Middle SDI
Qinghai	Middle SDI
Rājasthān, Urban	Middle SDI
Rio Grande do Norte	Middle SDI
Rondônia	Middle SDI
Roraima	Middle SDI
Samoa	Middle SDI
Sergipe	Middle SDI
Shaanxi	Middle SDI
Sichuan	Middle SDI
Sikkim, Rural	Middle SDI
Swaziland	Middle SDI
Syria	Middle SDI
Tajikistan	Middle SDI
Tamil Nādu, Rural	Middle SDI
Telangana, Urban	Middle SDI
The Six Minor Territories, Rural	Middle SDI
Tocantins	Middle SDI
Tonga	Middle SDI
Tripura, Urban	Middle SDI
Tunisia	Middle SDI
Uttar Pradesh, Urban	Middle SDI
Uttarakhand, Rural	Middle SDI
Veracruz de Ignacio de la Llave	Middle SDI
Vietnam	Middle SDI
West Bengal, Urban	Middle SDI
Xinjiang	Middle SDI
Yunnan	Middle SDI
Zacatecas	Middle SDI
Alagoas	Low-middle SDI
Angola	Low-middle SDI
Arunāchal Pradesh, Rural	Low-middle SDI

©2017 Global Burden of Disease Liver Cancer Collaboration. JAMA Oncology.

Location	SDI level
Assam, Rural	Low-middle SDI
Bangladesh	Low-middle SDI
Bhutan	Low-middle SDI
Bihār, Rural	Low-middle SDI
Bomet	Low-middle SDI
Bungoma	Low-middle SDI
Cambodia	Low-middle SDI
Cameroon	Low-middle SDI
Cape Verde	Low-middle SDI
Ceará	Low-middle SDI
Chhattīsgarh, Rural	Low-middle SDI
Congo	Low-middle SDI
Djibouti	Low-middle SDI
Elgeyo-Marakwet	Low-middle SDI
Embu	Low-middle SDI
Ghana	Low-middle SDI
Guatemala	Low-middle SDI
Guizhou	Low-middle SDI
Gujarāt, Rural	Low-middle SDI
Haiti	Low-middle SDI
HomaBay	Low-middle SDI
Jammu and Kashmīr, Rural	Low-middle SDI
Jharkhand, Rural	Low-middle SDI
Kajiado	Low-middle SDI
Kakamega	Low-middle SDI
Karnātaka, Rural	Low-middle SDI
Kericho	Low-middle SDI
Kiribati	Low-middle SDI
Kirinyaga	Low-middle SDI
Kisii	Low-middle SDI
Kisumu	Low-middle SDI
Kwale	Low-middle SDI
Lamu	Low-middle SDI
Laos	Low-middle SDI
Lesotho	Low-middle SDI
Machakos	Low-middle SDI
Madhya Pradesh, Rural	Low-middle SDI
Makueni	Low-middle SDI
Manipur, Rural	Low-middle SDI
Maranhão	Low-middle SDI

©2017 Global Burden of Disease Liver Cancer Collaboration. JAMA Oncology.

Location	SDI level
Meghālaya, Rural	Low-middle SDI
Meru	Low-middle SDI
Migori	Low-middle SDI
Mizoram, Rural	Low-middle SDI
Morocco	Low-middle SDI
Murang'a	Low-middle SDI
Myanmar	Low-middle SDI
Nakuru	Low-middle SDI
Nandi	Low-middle SDI
Nepal	Low-middle SDI
Nigeria	Low-middle SDI
Nyamira	Low-middle SDI
Nyandarua	Low-middle SDI
Orissa, Rural	Low-middle SDI
Pakistan	Low-middle SDI
Papua New Guinea	Low-middle SDI
Paraíba	Low-middle SDI
Piaui	Low-middle SDI
Rājasthān, Rural	Low-middle SDI
Sao Tome and Principe	Low-middle SDI
Siaya	Low-middle SDI
Solomon Islands	Low-middle SDI
Sudan	Low-middle SDI
TaitaTaveta	Low-middle SDI
Tanzania	Low-middle SDI
Telangana, Rural	Low-middle SDI
TharakaNithi	Low-middle SDI
Tibet	Low-middle SDI
Timor-Leste	Low-middle SDI
TransNzoia	Low-middle SDI
Tripura, Rural	Low-middle SDI
UasinGishu	Low-middle SDI
Uttar Pradesh, Rural	Low-middle SDI
Vanuatu	Low-middle SDI
Vihiga	Low-middle SDI
West Bengal, Rural	Low-middle SDI
Yemen	Low-middle SDI
Zambia	Low-middle SDI
Zimbabwe	Low-middle SDI
Afghanistan	Low SDI

©2017 Global Burden of Disease Liver Cancer Collaboration. JAMA Oncology.

Location	SDI level
Baringo	Low SDI
Benin	Low SDI
Burkina Faso	Low SDI
Burundi	Low SDI
Busia	Low SDI
Central African Republic	Low SDI
Chad	Low SDI
Comoros	Low SDI
Cote d'Ivoire	Low SDI
Democratic Republic of the Congo	Low SDI
Eritrea	Low SDI
Ethiopia	Low SDI
Garissa	Low SDI
Guinea	Low SDI
Guinea-Bissau	Low SDI
Isiolo	Low SDI
Kilifi	Low SDI
Kitui	Low SDI
Liberia	Low SDI
Madagascar	Low SDI
Malawi	Low SDI
Mali	Low SDI
Mandera	Low SDI
Marsabit	Low SDI
Mauritania	Low SDI
Mozambique	Low SDI
Narok	Low SDI
Niger	Low SDI
Rwanda	Low SDI
Samburu	Low SDI
Senegal	Low SDI
Sierra Leone	Low SDI
Somalia	Low SDI
South Sudan	Low SDI
TanaRiver	Low SDI
The Gambia	Low SDI
Togo	Low SDI
Turkana	Low SDI
Uganda	Low SDI
Wajir	Low SDI

©2017 Global Burden of Disease Liver Cancer Collaboration. JAMA Oncology.

Location	SDI level
WestPokot	Low SDI

©2017 Global Burden of Disease Liver Cancer Collaboration. JAMA Oncology.

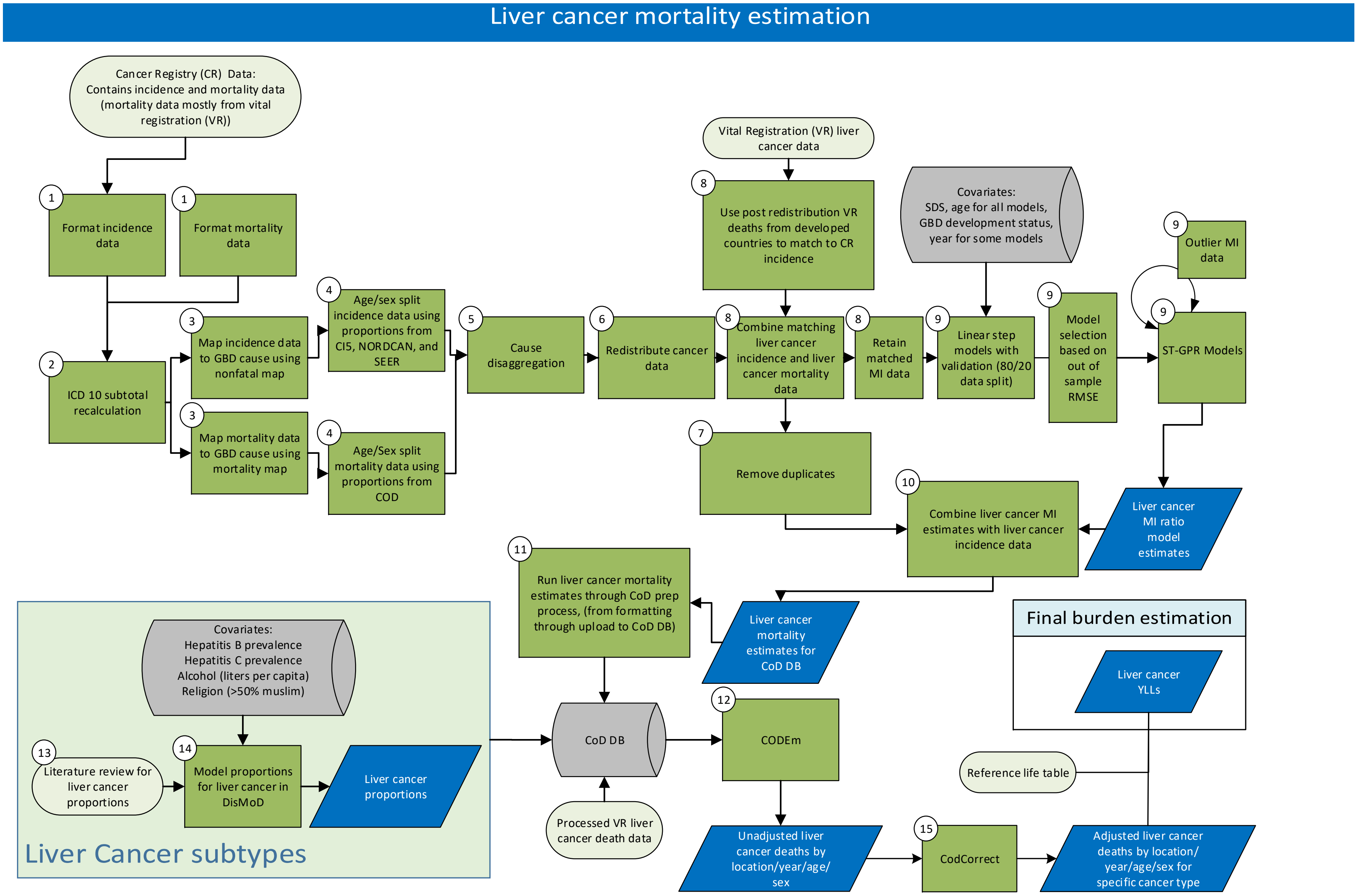

Abbreviations: ICD: International classification of diseases; CI5: Cancer Incidence in Five Continents, DB: database

eFigure 1: Flowchart, liver cancer mortality estimation

©2017 Global Burden of Disease Liver Cancer Collaboration. JAMA Oncology.

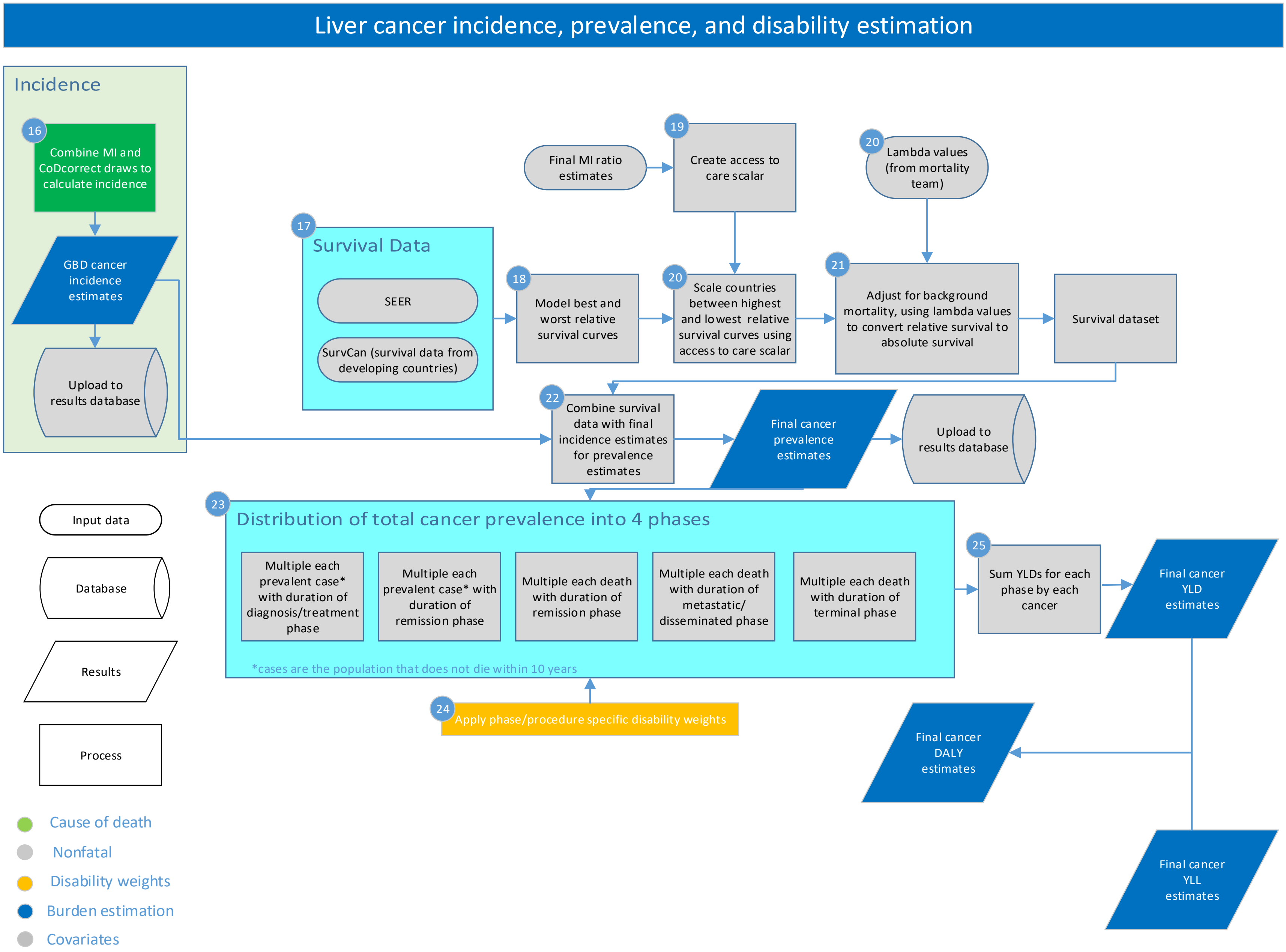

eFigure 2: Flowchart, liver cancer incidence to DALY estimation

©2017 Global Burden of Disease Liver Cancer Collaboration. JAMA Oncology.

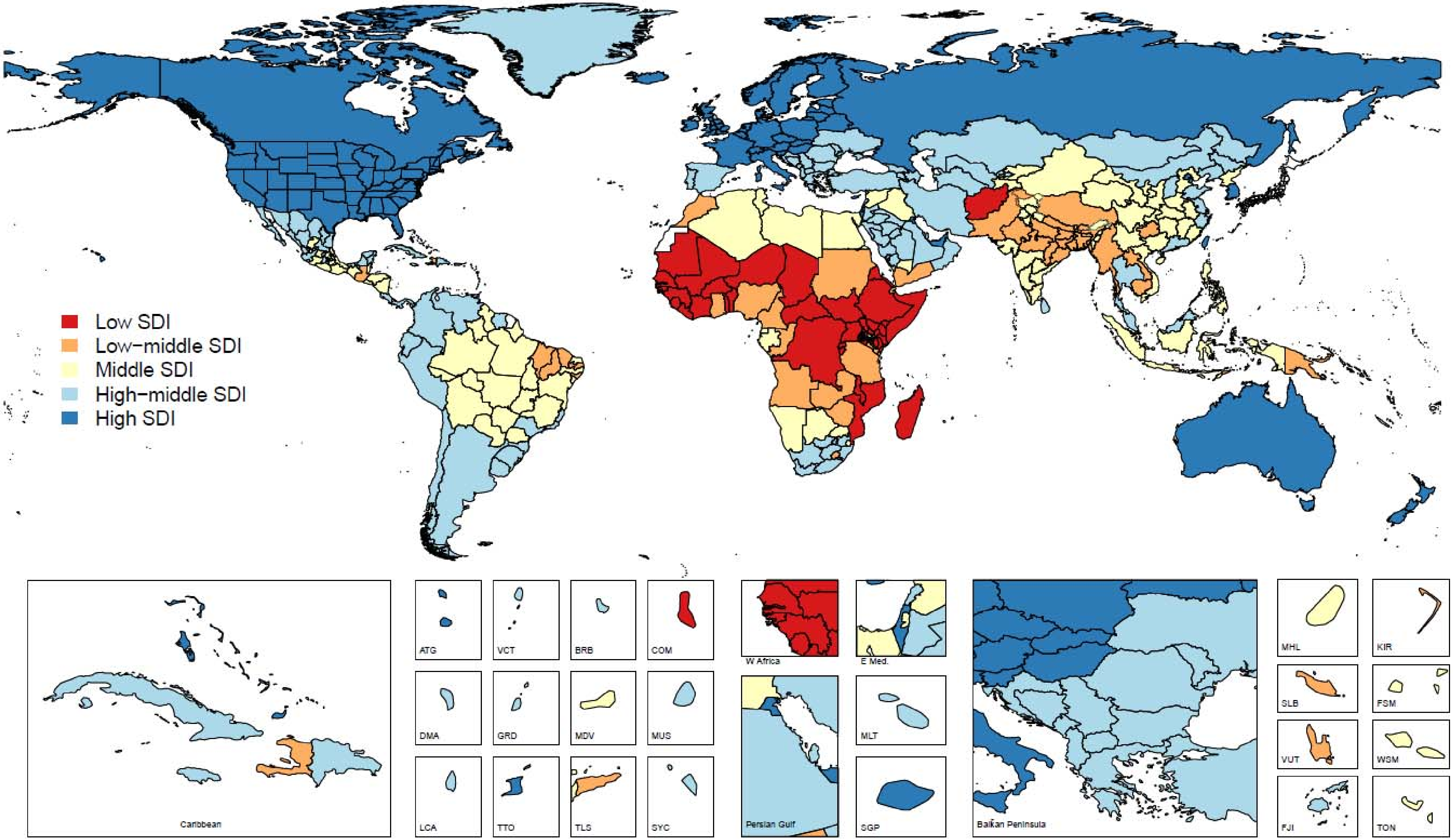

eFigure 3: Sociodemographic Index quintiles, 2015

©2017 Global Burden of Disease Liver Cancer Collaboration. JAMA Oncology.

Appendix B – Additional results

eTable 8: Age-standardized incidence rates with 95% uncertainty interval and male to female ratio by region, by sex, 2015

Region	Cancer	ASIR 2015, male	95% UI ASIR 2015, male	ASIR 2015, female	95% UI ASIR 2015, female	Ratio ASIR Male/ Female
Andean Latin America	Liver cancer	7	(5.8, 8.7)	7.5	(5.2, 9.9)	0.9
Andean Latin America	Liver cancer due to alcohol use	2.4	(2.0, 2.9)	1.1	(0.8, 1.4)	2.2
Andean Latin America	Liver cancer due to hepatitis B	3.1	(2.5, 3.9)	3.3	(2.2, 4.3)	0.9
Andean Latin America	Liver cancer due to hepatitis C	0.6	(0.4, 0.7)	1.2	(0.8, 1.6)	0.5
Andean Latin America	Liver cancer due to other causes	0.9	(0.7, 1.2)	1.9	(1.3, 2.7)	0.5
Australasia	Liver cancer	9.5	(6.9, 13.8)	3.6	(2.4, 6.2)	2.6
Australasia	Liver cancer due to alcohol use	4.7	(3.3, 6.6)	0.7	(0.5, 1.1)	6.7
Australasia	Liver cancer due to hepatitis B	1	(0.7, 1.3)	0.4	(0.3, 0.6)	2.5
Australasia	Liver cancer due to hepatitis C	3.1	(2.2, 4.7)	1.7	(1.1, 3.1)	1.8
Australasia	Liver cancer due to other causes	0.8	(0.6, 1.2)	0.8	(0.5, 1.4)	1.0
Caribbean	Liver cancer	7.4	(6.5, 8.7)	6.2	(5.1, 7.8)	1.2
Caribbean	Liver cancer due to alcohol use	2.5	(2.1, 3.1)	1	(0.8, 1.3)	2.5
Caribbean	Liver cancer due to hepatitis B	2.1	(1.8, 2.6)	1.4	(1.1, 1.8)	1.5
Caribbean	Liver cancer due to hepatitis C	1.7	(1.4, 2.1)	2.3	(1.8, 3.0)	0.7
Caribbean	Liver cancer due to other causes	1.1	(0.9, 1.3)	1.5	(1.2, 2.0)	0.7
Central Asia	Liver cancer	11.6	(10.0, 12.8)	6.2	(4.9, 7.3)	1.9
Central Asia	Liver cancer due to alcohol use	2.9	(2.5, 3.3)	0.8	(0.6, 1.0)	3.6
Central Asia	Liver cancer due to hepatitis B	3.6	(3.1, 4.1)	1.4	(1.1, 1.7)	2.6
Central Asia	Liver cancer due to hepatitis C	3.9	(3.3, 4.4)	2.9	(2.3, 3.5)	1.3
Central Asia	Liver cancer due to other causes	1.2	(1.0, 1.4)	1	(0.8, 1.2)	1.2
Central Europe	Liver cancer	9.6	(8.4, 11.4)	4.9	(3.9, 6.1)	2.0
Central Europe	Liver cancer due to alcohol use	5.1	(4.3, 6.2)	1.6	(1.2, 2.1)	3.2
Central Europe	Liver cancer due to hepatitis B	1.7	(1.4, 2.2)	0.8	(0.6, 1.0)	2.1
Central Europe	Liver cancer due to hepatitis C	2.1	(1.6, 2.7)	1.8	(1.4, 2.4)	1.2
Central Europe	Liver cancer due to other causes	0.7	(0.6, 0.9)	0.7	(0.6, 0.9)	1.0
Central Latin America	Liver cancer	6.6	(6.2, 7.7)	6.7	(5.9, 8.5)	1.0
Central Latin America	Liver cancer due to alcohol use	2.5	(2.3, 3.0)	1.2	(1.0, 1.5)	2.1
Central Latin America	Liver cancer due to hepatitis B	0.6	(0.5, 0.7)	0.4	(0.3, 0.5)	1.5
Central Latin America	Liver cancer due to hepatitis C	2.5	(2.3, 3.0)	3.7	(3.3, 4.9)	0.7
Central Latin America	Liver cancer due to other causes	1	(0.9, 1.2)	1.4	(1.2, 1.8)	0.7
Central Sub-Saharan Africa	Liver cancer	24.4	(13.4, 42.7)	10.6	(6.1, 18.5)	2.3
Central Sub-Saharan Africa	Liver cancer due to alcohol use	8.7	(4.8, 14.8)	2	(1.1, 3.3)	4.4
Central Sub-Saharan Africa	Liver cancer due to hepatitis B	4.3	(2.2, 7.8)	1.7	(1.0, 2.9)	2.5
Central Sub-Saharan Africa	Liver cancer due to hepatitis C	8.9	(4.9, 15.8)	4.9	(2.9, 8.7)	1.8
Central Sub-Saharan Africa	Liver cancer due to other causes	2.5	(1.4, 4.6)	2	(1.1, 3.5)	1.3
East Asia	Liver cancer	36.4	(28.6, 48.0)	12.5	(9.0, 18.2)	2.9
East Asia	Liver cancer due to alcohol use	13	(10.3, 17.2)	2.2	(1.5, 3.3)	5.9
East Asia	Liver cancer due to hepatitis B	15.1	(11.5, 20.2)	4.7	(3.4, 6.8)	3.2

©2017 Global Burden of Disease Liver Cancer Collaboration. JAMA Oncology.

Region	Cancer	ASIR 2015, male	95% UI ASIR 2015, male	ASIR 2015, female	95% UI ASIR 2015, female	Ratio ASIR Male/ Female
East Asia	Liver cancer due to hepatitis C	2.8	(2.2, 3.8)	1.7	(1.2, 2.4)	1.6
East Asia	Liver cancer due to other causes	5.4	(4.2, 7.2)	3.9	(2.8, 5.8)	1.4
Eastern Europe	Liver cancer	8.2	(6.9, 10.3)	4.2	(3.1, 6.4)	2.0
Eastern Europe	Liver cancer due to alcohol use	4.7	(4.0, 5.9)	1.8	(1.3, 2.8)	2.6
Eastern Europe	Liver cancer due to hepatitis B	1.6	(1.3, 2.1)	0.7	(0.5, 1.0)	2.3
Eastern Europe	Liver cancer due to hepatitis C	1.4	(1.1, 1.8)	1.3	(0.9, 2.1)	1.1
Eastern Europe	Liver cancer due to other causes	0.5	(0.4, 0.6)	0.5	(0.3, 0.7)	1.0
Eastern Sub-Saharan Africa	Liver cancer	13.8	(10.0, 18.4)	7.3	(4.9, 10.7)	1.9
Eastern Sub-Saharan Africa	Liver cancer due to alcohol use	5.7	(4.1, 7.6)	1.5	(1.0, 2.2)	3.8
Eastern Sub-Saharan Africa	Liver cancer due to hepatitis B	3.2	(2.2, 4.5)	1.4	(0.9, 2.1)	2.3
Eastern Sub-Saharan Africa	Liver cancer due to hepatitis C	3.4	(2.5, 4.6)	3.1	(2.1, 4.6)	1.1
Eastern Sub-Saharan Africa	Liver cancer due to other causes	1.4	(1.0, 1.9)	1.3	(0.9, 1.9)	1.1
High-income Asia Pacific	Liver cancer	40.1	(34.4, 48.3)	14.2	(10.9, 19.6)	2.8
High-income Asia Pacific	Liver cancer due to alcohol use	9	(7.5, 11.1)	1.5	(1.1, 2.0)	6.0
High-income Asia Pacific	Liver cancer due to hepatitis B	10.5	(9.0, 12.2)	2.9	(2.3, 3.7)	3.6
High-income Asia Pacific	Liver cancer due to hepatitis C	19	(15.7, 23.9)	8.7	(6.3, 12.7)	2.2
High-income Asia Pacific	Liver cancer due to other causes	1.7	(1.4, 2.0)	1.2	(0.9, 1.6)	1.4
High-income North America	Liver cancer	11.6	(9.4, 14.3)	4.3	(3.2, 7.0)	2.7
High-income North America	Liver cancer due to alcohol use	5.5	(4.5, 6.9)	0.6	(0.5, 0.9)	9.2
High-income North America	Liver cancer due to hepatitis B	1.4	(1.1, 1.7)	0.4	(0.3, 0.5)	3.5
High-income North America	Liver cancer due to hepatitis C	2.6	(2.1, 3.2)	1.9	(1.4, 3.2)	1.4
High-income North America	Liver cancer due to other causes	2.1	(1.7, 2.5)	1.4	(1.1, 2.3)	1.5
North Africa and Middle East	Liver cancer	8.1	(7.1, 9.1)	4.7	(3.7, 5.4)	1.7
North Africa and Middle East	Liver cancer due to alcohol use	1.4	(1.0, 1.8)	0.3	(0.2, 0.4)	4.7
North Africa and Middle East	Liver cancer due to hepatitis B	2.2	(1.7, 2.6)	1	(0.7, 1.2)	2.2
North Africa and Middle East	Liver cancer due to hepatitis C	3.5	(3.0, 4.1)	2.4	(1.9, 2.9)	1.5
North Africa and Middle East	Liver cancer due to other causes	1.1	(0.9, 1.3)	1	(0.7, 1.3)	1.1
Oceania	Liver cancer	12.5	(8.8, 17.8)	9.3	(5.7, 14.0)	1.3
Oceania	Liver cancer due to alcohol use	2.8	(2.0, 4.2)	0.9	(0.5, 1.3)	3.1
Oceania	Liver cancer due to hepatitis B	4.6	(3.2, 6.9)	2.7	(1.6, 4.2)	1.7
Oceania	Liver cancer due to hepatitis C	2.2	(1.6, 3.3)	2.4	(1.5, 3.7)	0.9
Oceania	Liver cancer due to other causes	2.8	(2.0, 4.1)	3.3	(2.0, 4.9)	0.8
South Asia	Liver cancer	4.7	(3.8, 6.2)	3.2	(2.4, 4.5)	1.5

©2017 Global Burden of Disease Liver Cancer Collaboration. JAMA Oncology.

Region	Cancer	ASIR 2015, male	95% UI ASIR 2015, male	ASIR 2015, female	95% UI ASIR 2015, female	Ratio ASIR Male/ Female
South Asia	Liver cancer due to alcohol use	1.1	(0.9, 1.5)	0.3	(0.2, 0.5)	3.7
South Asia	Liver cancer due to hepatitis B	1.9	(1.5, 2.5)	0.9	(0.7, 1.3)	2.1
South Asia	Liver cancer due to hepatitis C	1	(0.8, 1.3)	1.2	(0.9, 1.7)	0.8
South Asia	Liver cancer due to other causes	0.7	(0.6, 1.0)	0.8	(0.6, 1.1)	0.9
Southeast Asia	Liver cancer	20.2	(16.0, 25.4)	8.6	(6.1, 11.2)	2.3
Southeast Asia	Liver cancer due to alcohol use	7.5	(5.7, 9.7)	1.6	(1.0, 2.2)	4.7
Southeast Asia	Liver cancer due to hepatitis B	5.2	(3.9, 6.6)	1.7	(1.2, 2.3)	3.1
Southeast Asia	Liver cancer due to hepatitis C	4.1	(3.0, 5.4)	2.7	(1.8, 3.8)	1.5
Southeast Asia	Liver cancer due to other causes	3.5	(2.7, 4.5)	2.6	(1.9, 3.3)	1.3
Southern Latin America	Liver cancer	6.1	(5.4, 7.3)	4	(3.3, 5.2)	1.5
Southern Latin America	Liver cancer due to alcohol use	3.6	(3.1, 4.3)	0.9	(0.7, 1.1)	4.0
Southern Latin America	Liver cancer due to hepatitis B	0.5	(0.4, 0.6)	0.2	(0.2, 0.2)	2.5
Southern Latin America	Liver cancer due to hepatitis C	1.5	(1.3, 1.8)	2.4	(1.9, 3.1)	0.6
Southern Latin America	Liver cancer due to other causes	0.5	(0.4, 0.7)	0.6	(0.5, 0.8)	0.8
Southern Sub-Saharan Africa	Liver cancer	12.3	(9.8, 15.9)	5.8	(4.2, 8.2)	2.1
Southern Sub-Saharan Africa	Liver cancer due to alcohol use	6	(5.0, 7.7)	1.6	(1.2, 2.3)	3.8
Southern Sub-Saharan Africa	Liver cancer due to hepatitis B	3.4	(2.5, 4.5)	1.4	(1.0, 2.0)	2.4
Southern Sub-Saharan Africa	Liver cancer due to hepatitis C	1.9	(1.5, 2.5)	1.9	(1.4, 2.8)	1.0
Southern Sub-Saharan Africa	Liver cancer due to other causes	1	(0.7, 1.3)	0.9	(0.6, 1.4)	1.1
Tropical Latin America	Liver cancer	6.5	(5.5, 8.2)	4.5	(3.5, 6.5)	1.4
Tropical Latin America	Liver cancer due to alcohol use	2.6	(2.1, 3.2)	1	(0.7, 1.4)	2.6
Tropical Latin America	Liver cancer due to hepatitis B	1.5	(1.2, 1.9)	0.6	(0.5, 0.9)	2.5
Tropical Latin America	Liver cancer due to hepatitis C	1.9	(1.6, 2.5)	2	(1.5, 3.0)	1.0
Tropical Latin America	Liver cancer due to other causes	0.6	(0.5, 0.7)	0.9	(0.7, 1.3)	0.7
Western Europe	Liver cancer	13.3	(11.5, 15.4)	5.2	(4.1, 6.9)	2.6
Western Europe	Liver cancer due to alcohol use	5.1	(4.3, 6.0)	1	(0.8, 1.2)	5.1
Western Europe	Liver cancer due to hepatitis B	2.2	(1.7, 2.7)	0.7	(0.5, 0.9)	3.1
Western Europe	Liver cancer due to hepatitis C	4.9	(4.1, 6.1)	2.8	(2.1, 4.0)	1.8
Western Europe	Liver cancer due to other causes	1.1	(0.9, 1.4)	0.7	(0.5, 0.9)	1.6
Western Sub-Saharan Africa	Liver cancer	23.4	(16.8, 33.9)	10.9	(7.0, 15.8)	2.1
Western Sub-Saharan Africa	Liver cancer due to alcohol use	8.9	(5.9, 13.6)	2.8	(1.6, 4.6)	3.2
Western Sub-Saharan Africa	Liver cancer due to hepatitis B	9.2	(6.5, 13.3)	3.6	(2.3, 5.3)	2.6
Western Sub-Saharan Africa	Liver cancer due to hepatitis C	2.6	(1.8, 3.7)	2.1	(1.4, 3.2)	1.2
Western Sub-Saharan Africa	Liver cancer due to other causes	2.8	(2.1, 3.7)	2.3	(1.5, 3.2)	1.2

©2017 Global Burden of Disease Liver Cancer Collaboration. JAMA Oncology.

eTable 9: Global liver cancer incident cases, age-standardized incidence rate, deaths, age-standardized mortality rate, DALYs, and age-standardized DALYs attributable to each cause group by year and percent change (both sexes)

Year	Etiology	Incident cases (thousands with 95% UI)	ASIR (per 100,000 with 95% UI)	Deaths (thousands with 95% UI)	ASMR (per 100,000 with 95% UI)	DALYs (thousands with 95% UI)	Age-standardized DALY rate (per 100,000 with 95% UI)
1990	Hepatitis B	191 (154, 227)	4.8 (3.9, 5.7)	202 (165, 222)	5.2 (4.3, 5.7)	6685 (5312, 7361)	158.7 (126.9, 174.5)
	Hepatitis C	91 (84, 99)	2.7 (2.4, 2.9)	93 (85, 100)	2.7 (2.5, 2.9)	2121 (1903, 2303)	56.9 (51.4, 61.7)
	Alcohol	119 (102, 134)	3.3 (2.8, 3.7)	129 (112, 142)	3.6 (3.1, 3.9)	3265 (2766, 3606)	86.0 (73.4, 94.9)
	Other	88 (73, 102)	2.3 (2.0, 2.7)	94 (79, 103)	2.5 (2.1, 2.8)	2722 (2224, 2998)	67.0 (55.3, 73.9)
1995	Hepatitis B	215 (176, 248)	4.8 (4.0, 5.5)	224 (186, 241)	5.1 (4.3, 5.5)	7449 (5991, 8002)	157.2 (128.0, 168.8)
	Hepatitis C	108 (100, 115)	2.8 (2.6, 3.0)	108 (99, 116)	2.8 (2.6, 3.0)	2405 (2174, 2587)	57.9 (52.6, 62.2)
	Alcohol	141 (123, 156)	3.5 (3.1, 3.9)	152 (132, 164)	3.8 (3.3, 4.1)	3849 (3244, 4171)	90.6 (77.0, 98.0)
	Other	96 (81, 109)	2.3 (2.0, 2.6)	102 (86, 110)	2.5 (2.1, 2.7)	2916 (2409, 3183)	64.1 (53.6, 69.9)
2000	Hepatitis B	243 (195, 278)	4.9 (3.9, 5.5)	251 (203, 268)	5.1 (4.2, 5.5)	8230 (6535, 8788)	155.8 (124.6, 166.2)
	Hepatitis C	124 (113, 133)	2.9 (2.6, 3.1)	125 (114, 133)	2.9 (2.7, 3.1)	2711 (2431, 2917)	58.4 (52.6, 62.7)
	Alcohol	166 (140, 183)	3.6 (3.1, 4.0)	178 (150, 191)	3.9 (3.3, 4.2)	4424 (3663, 4768)	92.9 (77.4, 100.1)
	Other	115 (96, 131)	2.5 (2.1, 2.8)	120 (102, 130)	2.6 (2.2, 2.8)	3351 (2723, 3639)	66.2 (54.6, 72.0)
2005	Hepatitis B	257 (215, 297)	4.6 (3.9, 5.3)	263 (224, 283)	4.8 (4.1, 5.1)	8421 (7092, 9020)	143.4 (121.3, 153.7)
	Hepatitis C	139 (127, 149)	2.9 (2.6, 3.1)	138 (126, 146)	2.8 (2.6, 3.0)	2897 (2608, 3109)	55.5 (50.1, 59.5)
	Alcohol	185 (161, 204)	3.6 (3.1, 4.0)	195 (169, 208)	3.8 (3.3, 4.1)	4787 (4075, 5169)	89.2 (76.2, 96.3)

©2017 Global Burden of Disease Liver Cancer Collaboration. JAMA Oncology.

	Other	127 (108, 145)	2.4 (2.1, 2.8)	131 (114, 142)	2.5 (2.2, 2.7)	3539 (3023, 3830)	62.9 (54.1, 68.1)
2010	Hepatitis B	260 (229, 302)	4.2 (3.7, 4.8)	261 (236, 280)	4.2 (3.8, 4.5)	8107 (7372, 8686)	124.3 (112.9, 133.4)
	Hepatitis C	162 (149, 173)	2.9 (2.7, 3.1)	150 (138, 160)	2.7 (2.5, 2.9)	3051 (2764, 3264)	51.7 (47.0, 55.2)
	Alcohol	210 (190, 232)	3.6 (3.3, 4.0)	215 (195, 232)	3.7 (3.4, 4.0)	5203 (4707, 5657)	85.7 (77.6, 93.0)
	Other	129 (113, 147)	2.2 (1.9, 2.5)	130 (117, 140)	2.2 (2.0, 2.4)	3356 (3014, 3624)	53.6 (48.1, 57.8)
2015	Hepatitis B	272 (235, 327)	3.9 (3.4, 4.7)	265 (241, 291)	3.8 (3.5, 4.2)	8029 (7280, 8796)	110.6 (100.4, 121.2)
	Hepatitis C	196 (178, 217)	3.1 (2.8, 3.4)	167 (154, 178)	2.6 (2.4, 2.8)	3324 (3012, 3574)	49.3 (44.9, 52.9)
	Alcohol	249 (221, 287)	3.7 (3.3, 4.3)	245 (225, 267)	3.7 (3.4, 4.0)	5889 (5368, 6441)	84.8 (77.5, 92.7)
	Other	137 (120, 158)	2.1 (1.8, 2.4)	133 (120, 144)	2.0 (1.8, 2.2)	3336 (3019, 3638)	47.4 (42.9, 51.6)
Percent change between 1990 and 2015 (%)	Hepatitis B	42.4 (14.8, 78.7)	-18.9 (-34.1, 0.7)	30.1 (19.0, 53.8)	-26.5 (-32.7, -14.3)	20.1 (8.3, 45.9)	-30.3 (-37.1, -16.4)
	Hepatitis C	114.3 (94.0, 138.0)	15.7 (4.3, 29.4)	79.3 (70.3, 92.7)	-3.3 (-8.0, 3.4)	56.7 (47.3, 69.6)	-13.4 (-18.3, -6.5)
	Alcohol	109.3 (79.6, 146.0)	13.5 (-2.1, 32.5)	89.5 (74.1, 110.1)	2.9 (-5.2, 13.4)	80.3 (64.0, 104.5)	-1.4 (-10.2, 11.3)
	Other	56.4 (30.3, 89.5)	-12.3 (-26.8, 5.7)	40.3 (29.9, 59.9)	-21.8 (-27.3, -12.0)	22.6 (11.5, 42.7)	-29.2 (-35.4, -18.4)

Abbreviations UI: uncertainty interval, ASIR: age-standardized incidence rate, ASMR: age-standardized mortality rate, DALY: disability-adjusted life-year

©2017 Global Burden of Disease Liver Cancer Collaboration. JAMA Oncology.

eTable 10: Contribution of hepatitis B, C, alcohol and other causes on absolute liver cancer deaths, 2015, both sexes, by location

Location	Liver Cancer Due To Alcohol Use	Liver Cancer Due To Hepatitis B	Liver Cancer Due To Hepatitis C	Liver Cancer Due To Other Causes
Afghanistan	11%	36%	32%	21%
Albania	20%	23%	41%	16%
Algeria	14%	32%	35%	19%
American Samoa	16%	39%	18%	27%
Andorra	47%	15%	31%	7%
Angola	37%	20%	32%	12%
Antigua and Barbuda	19%	29%	31%	22%
Argentina	44%	6%	39%	10%
Armenia	15%	28%	42%	15%
Australia	38%	9%	40%	13%
Austria	49%	15%	30%	6%
Azerbaijan	15%	32%	40%	14%
Bahrain	17%	39%	28%	16%
Bangladesh	11%	33%	34%	22%
Barbados	24%	25%	31%	19%
Belarus	63%	13%	19%	5%
Belgium	48%	16%	28%	8%
Belize	23%	31%	27%	20%
Benin	25%	47%	11%	18%
Bermuda	27%	25%	30%	18%
Bhutan	19%	34%	28%	19%
Bolivia	18%	48%	13%	22%
Bosnia and Herzegovina	29%	20%	37%	14%
Botswana	43%	32%	15%	10%
Brazil	32%	20%	35%	13%
Brunei	8%	48%	34%	10%
Bulgaria	40%	18%	30%	12%
Burkina Faso	24%	48%	12%	16%
Burundi	47%	20%	23%	10%
Cambodia	34%	25%	19%	22%
Cameroon	47%	32%	11%	11%
Canada	31%	9%	34%	27%
Cape Verde	47%	31%	9%	12%
Central African Republic	24%	20%	40%	15%
Chad	19%	51%	12%	18%
Chile	38%	6%	43%	12%
China	33%	41%	8%	18%
Colombia	26%	15%	42%	17%
Comoros	11%	34%	36%	19%
Congo	27%	21%	39%	14%

©2017 Global Burden of Disease Liver Cancer Collaboration. JAMA Oncology.

Location	Liver Cancer Due To Alcohol Use	Liver Cancer Due To Hepatitis B	Liver Cancer Due To Hepatitis C	Liver Cancer Due To Other Causes
Costa Rica	28%	9%	45%	17%
Cote d'Ivoire	35%	39%	11%	15%
Croatia	54%	19%	17%	10%
Cuba	22%	25%	32%	20%
Cyprus	32%	19%	39%	10%
Czech Republic	51%	16%	23%	10%
Democratic Republic of the Congo	27%	21%	39%	14%
Denmark	49%	15%	29%	6%
Djibouti	13%	33%	36%	18%
Dominica	24%	27%	30%	19%
Dominican Republic	26%	26%	29%	19%
Ecuador	23%	45%	12%	20%
Egypt	12%	13%	63%	12%
El Salvador	17%	11%	50%	23%
England	36%	17%	38%	9%
Equatorial Guinea	47%	17%	27%	9%
Eritrea	20%	30%	32%	18%
Estonia	61%	11%	22%	6%
Ethiopia	32%	26%	29%	13%
Federated States of Micronesia	18%	36%	19%	27%
Fiji	21%	35%	18%	26%
Finland	37%	16%	38%	9%
France	37%	17%	36%	9%
Gabon	45%	15%	31%	9%
Georgia	19%	28%	39%	13%
Germany	44%	8%	33%	14%
Ghana	33%	45%	9%	13%
Greece	31%	33%	27%	9%
Greenland	43%	10%	28%	20%
Grenada	30%	26%	27%	17%
Guam	17%	36%	19%	27%
Guatemala	15%	12%	49%	25%
Guinea	12%	56%	12%	20%
Guinea-Bissau	18%	51%	13%	19%
Guyana	30%	29%	24%	16%
Haiti	24%	29%	28%	19%
Honduras	17%	11%	49%	23%
Hungary	52%	11%	29%	8%
Iceland	26%	19%	42%	12%
India	21%	42%	20%	18%
Indonesia	13%	13%	34%	39%
Iran	6%	44%	24%	26%

©2017 Global Burden of Disease Liver Cancer Collaboration. JAMA Oncology.

Location	Liver Cancer Due To Alcohol Use	Liver Cancer Due To Hepatitis B	Liver Cancer Due To Hepatitis C	Liver Cancer Due To Other Causes
Iraq	12%	37%	32%	19%
Ireland	36%	18%	37%	9%
Israel	15%	20%	49%	17%
Italy	20%	8%	63%	9%
Jamaica	19%	28%	31%	22%
Japan	17%	8%	69%	6%
Jordan	15%	35%	31%	19%
Kazakhstan	36%	23%	30%	10%
Kenya	37%	25%	23%	15%
Kiribati	16%	40%	17%	27%
Kuwait	15%	37%	31%	18%
Kyrgyzstan	11%	34%	40%	15%
Laos	36%	28%	17%	20%
Latvia	34%	19%	36%	11%
Lebanon	17%	28%	40%	15%
Lesotho	32%	34%	21%	13%
Liberia	38%	39%	10%	14%
Libya	15%	33%	34%	18%
Lithuania	59%	13%	22%	6%
Luxembourg	38%	17%	36%	9%
Macedonia	29%	21%	36%	14%
Madagascar	23%	29%	31%	17%
Malawi	19%	36%	28%	17%
Malaysia	12%	37%	14%	37%
Maldives	15%	30%	25%	31%
Mali	13%	47%	17%	23%
Malta	24%	19%	44%	12%
Marshall Islands	15%	39%	18%	28%
Mauritania	11%	53%	14%	22%
Mauritius	29%	23%	22%	26%
Mexico	30%	4%	49%	17%
Moldova	61%	15%	18%	6%
Mongolia	19%	30%	39%	12%
Montenegro	41%	17%	30%	11%
Morocco	14%	31%	36%	19%
Mozambique	23%	26%	36%	15%
Myanmar	15%	28%	24%	33%
Namibia	44%	31%	15%	10%
Nepal	24%	33%	24%	19%
Netherlands	35%	18%	37%	10%
New Zealand	44%	10%	34%	12%
Nicaragua	16%	11%	49%	23%
Niger	13%	52%	14%	22%
Nigeria	39%	42%	11%	9%

©2017 Global Burden of Disease Liver Cancer Collaboration. JAMA Oncology.

Location	Liver Cancer Due To Alcohol Use	Liver Cancer Due To Hepatitis B	Liver Cancer Due To Hepatitis C	Liver Cancer Due To Other Causes
North Korea	17%	42%	17%	24%
Northern Ireland	34%	18%	38%	10%
Northern Mariana Islands	15%	46%	14%	26%
Norway	25%	19%	44%	12%
Oman	17%	39%	28%	16%
Pakistan	7%	16%	54%	23%
Palestine	13%	38%	30%	19%
Panama	34%	9%	42%	15%
Papua New Guinea	16%	39%	18%	27%
Paraguay	28%	21%	37%	15%
Peru	26%	44%	11%	19%
Philippines	35%	29%	16%	20%
Poland	42%	16%	31%	12%
Portugal	37%	18%	36%	9%
Puerto Rico	33%	22%	29%	16%
Qatar	18%	38%	28%	15%
Romania	54%	13%	25%	8%
Russia	53%	15%	24%	8%
Rwanda	39%	17%	34%	10%
Saint Lucia	36%	24%	26%	15%
Saint Vincent and the Grenadines	24%	29%	28%	19%
Samoa	19%	39%	17%	25%
Sao Tome and Principe	45%	33%	9%	12%
Saudi Arabia	17%	41%	17%	25%
Scotland	36%	18%	37%	9%
Senegal	14%	53%	7%	25%
Serbia	38%	11%	42%	9%
Seychelles	35%	25%	19%	21%
Sierra Leone	37%	39%	11%	14%
Singapore	10%	57%	22%	11%
Slovakia	51%	15%	26%	9%
Slovenia	51%	14%	26%	9%
Solomon Islands	13%	44%	16%	27%
Somalia	15%	36%	30%	18%
South Africa	44%	24%	23%	8%
South Korea	21%	54%	20%	5%
South Sudan	16%	30%	37%	17%
Spain	21%	10%	62%	8%
Sri Lanka	26%	24%	22%	28%
Sudan	18%	35%	30%	16%
Suriname	24%	29%	28%	19%
Swaziland	30%	36%	21%	14%

©2017 Global Burden of Disease Liver Cancer Collaboration. JAMA Oncology.

Location	Liver Cancer Due To Alcohol Use	Liver Cancer Due To Hepatitis B	Liver Cancer Due To Hepatitis C	Liver Cancer Due To Other Causes
Sweden	33%	17%	39%	10%
Switzerland	39%	17%	36%	9%
Syria	14%	32%	34%	19%
Taiwan	27%	27%	27%	19%
Tajikistan	10%	37%	38%	15%
Tanzania	46%	21%	23%	10%
Thailand	39%	29%	21%	11%
The Bahamas	19%	30%	30%	21%
The Gambia	14%	60%	9%	16%
Timor-Leste	19%	28%	23%	30%
Togo	25%	47%	11%	17%
Tonga	15%	37%	20%	28%
Trinidad and Tobago	23%	27%	30%	20%
Tunisia	18%	20%	44%	18%
Turkey	19%	26%	44%	11%
Turkmenistan	11%	38%	37%	15%
Uganda	45%	23%	21%	10%
Ukraine	47%	17%	27%	9%
United Arab Emirates	21%	44%	22%	13%
United Kingdom	36%	17%	38%	9%
United States	37%	9%	31%	22%
Uruguay	39%	7%	41%	13%
Uzbekistan	12%	37%	36%	15%
Vanuatu	13%	39%	18%	29%
Venezuela	35%	9%	41%	15%
Vietnam	37%	28%	16%	19%
Virgin Islands, U.S.	36%	22%	27%	15%
Wales	37%	17%	38%	9%
Yemen	8%	44%	35%	12%
Zambia	31%	25%	29%	14%
Zimbabwe	30%	38%	15%	16%

©2017 Global Burden of Disease Liver Cancer Collaboration. JAMA Oncology.

eTable 11: Comparison between liver cancer deaths estimates for GBD and Globocan for the year 2012, both sexes combined

Location	GBD Deaths 2012	Lower 95% uncertainty interval	Upper 95% uncertainty interval	Globocan Deaths 2012 (green if between GBD 95% UI)	Difference between GBD and Globocan (%)
Global	764,745	708,602	810,269	745,533	3%
Afghanistan	1,496	713	2,196	723	107%
Albania	271	226	395	246	10%
Algeria	678	521	795	409	66%
Angola	1,637	574	4,399	490	234%
Argentina	2,085	1,925	2,257	2,012	4%
Armenia	341	274	384	440	-23%
Australia	1,253	1,130	1,371	1,538	-19%
Austria	871	801	941	894	-3%
Azerbaijan	497	424	647	441	13%
Bahrain	19	16	23	22	-13%
Bangladesh	2,964	2,038	3,653	2,877	3%
Barbados	18	16	20	11	63%
Belarus	451	402	514	290	56%
Belgium	816	747	889	761	7%
Belize	17	15	23	15	11%
Benin	966	487	1,752	686	41%
Bhutan	16	11	22	24	-33%
Bolivia	539	402	667	263	105%
Bosnia and Herzegovina	384	264	451	477	-20%
Botswana	124	33	393	73	69%
Brazil	9,310	8,767	10,909	10,070	-8%
Brunei	25	22	31	19	30%
Bulgaria	904	833	978	858	5%
Burkina Faso	1,316	765	2,095	1,219	8%
Burundi	484	279	770	254	91%
Cambodia	661	498	783	2,155	-69%
Cameroon	3,054	1,632	5,168	716	327%
Canada	1,962	1,758	2,153	2,276	-14%
Cape Verde	39	32	44	56	-30%
Central African Republic	552	253	918	169	226%
Chad	1,070	497	1,922	426	151%
Chile	1,211	1,104	1,321	1,218	-1%
China	380,337	352,544	419,744	383,203	-1%
Colombia	1,855	1,711	2,010	1,728	7%

©2017 Global Burden of Disease Liver Cancer Collaboration. JAMA Oncology.

Comoros	36	22	52	15	137%
Congo	371	218	614	225	65%
Costa Rica	252	231	276	197	28%
Cote d'Ivoire	1,296	732	2,042	2,141	-39%
Croatia	477	429	518	447	7%
Cuba	820	754	900	804	2%
Cyprus	48	37	55	47	2%
Czech Republic	857	786	925	697	23%
Democratic Republic of the Congo	4,765	2,831	7,639	3,438	39%
Denmark	341	313	369	303	13%
Djibouti	58	25	114	18	220%
Dominican Republic	556	450	616	810	-31%
Ecuador	961	683	1,107	1,018	-6%
Egypt	8,121	7,197	9,158	16,770	-52%
El Salvador	289	243	324	429	-33%
Equatorial Guinea	69	28	194	20	243%
Eritrea	264	120	493	77	243%
Estonia	84	76	94	92	-9%
Ethiopia	4,436	2,537	7,109	926	379%
Fiji	37	30	44	60	-38%
Finland	440	401	484	484	-9%
France	9,553	8,645	10,434	8,050	19%
Gabon	158	91	274	28	465%
Georgia	389	343	433	421	-8%
Germany	7,920	7,256	8,621	7,725	3%
Ghana	2,160	1,200	3,562	1,856	16%
Greece	2,103	1,849	2,374	1,398	50%
Guam	20	16	23	13	52%
Guatemala	1,394	1,305	1,488	1,476	-6%
Guinea	2,040	1,307	3,018	1,050	94%
Guinea-Bissau	226	72	647	124	82%
Guyana	30	26	41	32	-6%
Haiti	516	343	664	436	18%
Honduras	219	172	281	572	-62%
Hungary	844	776	909	621	36%
Iceland	11	10	12	12	-9%
India	32,726	30,216	37,726	26,763	22%
Indonesia	12,193	9,198	14,699	17,175	-29%
Iran	2,183	1,670	2,809	1,492	46%
Iraq	1,267	950	1,643	645	96%
Ireland	212	192	233	237	-11%
Israel	307	279	336	296	4%
Italy	11,061	9,982	12,212	9,198	20%

©2017 Global Burden of Disease Liver Cancer Collaboration. JAMA Oncology.

Jamaica	118	98	170	130	-10%
Japan	36,056	34,885	37,274	32,518	11%
Jordan	80	69	96	154	-48%
Kazakhstan	1,115	1,033	1,208	1,275	-13%
Kenya	1,253	797	1,524	1,037	21%
Kuwait	59	54	64	61	-3%
Kyrgyzstan	271	244	307	313	-13%
Laos	311	218	395	2,022	-85%
Latvia	128	115	140	135	-5%
Lebanon	149	109	201	109	37%
Lesotho	125	73	190	79	59%
Liberia	366	233	531	373	-2%
Libya	209	140	254	201	4%
Lithuania	168	154	184	181	-7%
Luxembourg	39	35	42	66	-41%
Macedonia	190	170	216	185	3%
Madagascar	945	513	1,478	452	109%
Malawi	520	324	806	203	156%
Malaysia	1,514	1,307	1,828	1,750	-13%
Maldives	5	4	6	6	-12%
Mali	2,249	1,427	3,408	345	552%
Malta	18	16	20	22	-18%
Mauritania	313	169	524	274	14%
Mauritius	49	45	53	70	-30%
Mexico	5,919	5,753	6,099	6,068	-2%
Moldova	403	374	435	503	-20%
Mongolia	1,512	734	1,808	1,345	12%
Montenegro	43	37	55	68	-36%
Morocco	1,629	1,165	2,193	321	408%
Mozambique	2,432	1,318	3,718	606	301%
Myanmar	4,733	2,794	7,164	4,652	2%
Namibia	22	13	35	27	-17%
Nepal	569	395	741	177	221%
Netherlands	807	748	870	678	19%
New Zealand	216	196	236	236	-9%
Nicaragua	283	257	340	418	-32%
Niger	1,339	828	2,076	489	174%
Nigeria	8,628	4,535	15,029	11,663	-26%
North Korea	8,300	6,412	10,730	4,818	72%
Norway	200	183	220	173	16%
Oman	72	58	84	57	26%
Pakistan	5,650	4,416	6,908	4,115	37%
Palestine	115	91	150	70	64%

©2017 Global Burden of Disease Liver Cancer Collaboration. JAMA Oncology.

Panama	149	128	198	170	-12%
Papua New Guinea	384	217	609	521	-26%
Paraguay	155	135	180	159	-2%
Peru	1,515	1,327	1,733	1,726	-12%
Philippines	6,334	5,865	6,793	7,434	-15%
Poland	2,002	1,862	2,155	2,068	-3%
Portugal	990	912	1,081	908	9%
Puerto Rico	365	325	404	272	34%
Qatar	38	22	48	47	-20%
Romania	2,543	2,337	2,742	2,830	-10%
Russia	9,423	8,748	10,165	8,521	11%
Rwanda	445	258	735	694	-36%
Samoa	9	6	16	4	125%
Saudi Arabia	680	482	772	680	0%
Senegal	1,297	706	2,247	696	86%
Serbia	824	701	910	852	-3%
Sierra Leone	596	382	838	513	16%
Singapore	545	493	608	747	-27%
Slovakia	370	331	417	347	7%
Slovenia	166	147	183	189	-12%
Solomon Islands	33	19	53	39	-14%
Somalia	610	181	1,504	178	243%
South Africa	2,316	2,040	2,785	1,877	23%
South Korea	13,404	12,437	14,415	12,275	9%
South Sudan	628	209	1,819	359	75%
Spain	5,004	4,577	5,469	4,536	10%
Sri Lanka	517	449	585	771	-33%
Sudan	1,316	784	2,038	887	48%
Suriname	27	23	40	34	-19%
Swaziland	68	31	114	71	-5%
Sweden	619	571	674	621	0%
Switzerland	712	650	775	690	3%
Syria	276	187	328	537	-49%
Tajikistan	316	234	373	263	20%
Tanzania	2,163	1,209	3,972	565	283%
Thailand	26,821	16,456	31,557	19,442	38%
The Gambia	414	282	600	225	84%
Timor-Leste	49	28	71	41	20%
Togo	616	388	914	471	31%
Trinidad and Tobago	46	41	52	35	31%
Tunisia	533	291	694	107	398%
Turkey	2,289	2,037	2,533	2,117	8%
Turkmenistan	170	151	216	248	-32%

©2017 Global Burden of Disease Liver Cancer Collaboration. JAMA Oncology.

Uganda	1,658	887	2,508	1,292	28%
Ukraine	1,965	1,813	2,141	1,683	17%
United Arab Emirates	155	106	224	67	131%
United Kingdom	4,319	4,137	4,472	4,059	6%
United States	22,252	21,480	23,030	24,312	-8%
Uruguay	133	120	146	99	34%
Uzbekistan	927	808	1,167	1,041	-11%
Vanuatu	15	10	24	26	-41%
Venezuela	997	928	1,074	940	6%
Vietnam	12,359	8,331	18,096	20,920	-41%
Yemen	790	410	1,344	329	140%
Zambia	762	489	1,199	221	245%
Zimbabwe	1,231	770	1,777	520	137%

©2017 Global Burden of Disease Liver Cancer Collaboration. JAMA Oncology.

Country	Liver cancer incidence ranking	Liver cancer mortality ranking
Global	6	4
High SDI	10	6
High-middle SDI	6	2
Middle SDI	4	2
Low-middle SDI	9	4
Low SDI	4	1
**South Asia**	14	8
India	14	8
Pakistan	13	6
Bangladesh	11	9
Nepal	11	7
Bhutan	11	6
**East Asia**	3	2
China	3	2
North Korea	3	2
Taiwan	3	1
**Southeast Asia**	4	2
Indonesia	8	2
Philippines	7	4
Vietnam	5	2
Thailand	3	1
Myanmar	8	6
Malaysia	9	4
Sri Lanka	18	10
Cambodia	8	5
Laos	8	5
Mauritius	14	7
Timor-Leste	8	2
Maldives	11	6
Seychelles	20	11
**North Africa and Middle East**	10	6
Egypt	4	1
Iran	14	9
Turkey	14	9
Sudan	7	4
Algeria	15	9
Iraq	11	5
Morocco	10	7
Afghanistan	6	4
Saudi Arabia	7	3
Yemen	8	4
Syria	12	9
Tunisia	10	6
United Arab Emirates	14	7
Jordan	20	12
Libya	12	4
Lebanon	18	12
Palestine	9	6
Oman	9	3
Kuwait	10	5
Qatar	8	5

©2017 Global Burden of Disease Liver Cancer Collaboration. JAMA Oncology.

Country	Liver cancer incidence ranking	Liver cancer mortality ranking
Bahrain	17	7
**Western Europe**	12	8
Germany	13	8
France	12	6
United Kingdom	15	12
Italy	8	6
England	15	12
Spain	9	8
Netherlands	20	14
Belgium	18	10
Greece	6	4
Portugal	12	8
Sweden	15	13
Austria	14	8
Switzerland	11	8
Israel	18	11
Denmark	17	14
Finland	14	8
Scotland	15	12
Norway	20	15
Ireland	18	12
Wales	15	12
Northern Ireland	15	12
Cyprus	14	9
Luxembourg	14	8
Malta	18	14
Iceland	17	15
Andorra	17	11
**Western SSA**	4	1
Nigeria	5	1
Ghana	5	1
Cameroon	2	1
Cote d'Ivoire	5	3
Niger	4	1
Burkina Faso	4	1
Mali	1	1
Senegal	4	1
Chad	4	1
Guinea	1	1
Benin	4	1
Togo	4	1
Sierra Leone	4	1
Liberia	4	1
Mauritania	4	1
The Gambia	1	1
Guinea-Bissau	4	1
Cape Verde	6	4
Sao Tome and Principe	20	17
**Eastern SSA**	5	2
Ethiopia	3	2
Tanzania	4	2

©2017 Global Burden of Disease Liver Cancer Collaboration. JAMA Oncology.

Country	Liver cancer incidence ranking	Liver cancer mortality ranking
Kenya	7	3
Uganda	7	4
Mozambique	3	1
Madagascar	3	2
Malawi	7	3
Zambia	8	4
South Sudan	4	2
Rwanda	5	2
Burundi	4	2
Somalia	4	2
Eritrea	4	2
Djibouti	5	1
Comoros	3	2
**High-income North America**	14	8
United States	13	8
Canada	17	12
Greenland	14	7
**Central Latin America**	9	6
Mexico	9	6
Colombia	9	7
Venezuela	14	9
Guatemala	4	2
Honduras	12	8
El Salvador	10	8
Nicaragua	7	3
Costa Rica	11	6
Panama	13	8
**Eastern Europe**	15	8
Russia	14	7
Ukraine	17	14
Belarus	21	17
Moldova	6	5
Lithuania	15	14
Latvia	14	12
Estonia	14	12
**Tropical Latin America**	16	8
Brazil	16	8
Paraguay	19	10
**High-income Asia Pacific**	6	4
Japan	6	4
South Korea	4	2
Singapore	5	3
Brunei	8	3
**Central Europe**	15	7
Poland	20	14
Romania	6	6
Czech Republic	14	9
Hungary	17	11
Serbia	13	8
Bulgaria	14	7
Slovakia	17	9

©2017 Global Burden of Disease Liver Cancer Collaboration. JAMA Oncology.

Country	Liver cancer incidence ranking	Liver cancer mortality ranking
Croatia	10	7
Bosnia and Herzegovina	6	6
Albania	6	4
Macedonia	9	6
Slovenia	13	8
Montenegro	12	8
**Central SSA**	3	1
Democratic Republic of Congo	4	1
Angola	3	1
Central African Republic	4	1
Congo	3	1
Gabon	5	1
Equatorial Guinea	3	1
Central Asia	9	3
Uzbekistan	13	7
Kazakhstan	13	6
Azerbaijan	13	6
Tajikistan	4	2
Kyrgyzstan	7	3
Turkmenistan	14	6
Georgia	13	5
Armenia	10	6
Mongolia	1	1
**Southern Sub-Saharan Africa**	9	7
South Africa	14	7
Zimbabwe	5	2
Namibia	16	10
Botswana	10	5
Lesotho	9	6
Swaziland	10	5
**Southern Latin America**	17	11
Argentina	18	12
Chile	14	8
Uruguay	24	18
**Andean Latin America**	10	6
Peru	9	5
Ecuador	10	5
Bolivia	10	7
Caribbean	11	7
Cuba	17	7
Haiti	9	7
Dominican Republic	8	5
Puerto Rico	9	5
Jamaica	13	10
Trinidad and Tobago	15	11
Guyana	11	7
Suriname	9	6
The Bahamas	17	7
Belize	10	6
Barbados	18	12
Saint Lucia	18	10

©2017 Global Burden of Disease Liver Cancer Collaboration. JAMA Oncology.

Country	Liver cancer incidence ranking	Liver cancer mortality ranking
Saint Vincent and the Grenadines	17	10
Grenada	14	10
Virgin Islands, U.S.	20	12
Antigua and Barbuda	16	8
Dominica	14	9
Bermuda	22	13
**Australasia**	15	12
Australia	16	12
New Zealand	14	14
**Oceania**	6	4
Papua New Guinea	6	4
Fiji	11	6
Solomon Islands	6	4
Vanuatu	7	4
Samoa	9	3
Guam	7	3
Northern Mariana Islands	11	3
Kiribati	4	2
Tonga	3	2
Federated States of Micronesia	9	3
American Samoa	7	3
Marshall Islands	8	3

Colors correspond to the ranking, with dark red as the most common cancer and dark green as the least common cancer for the location indicated. Rankings include 31 cancer groups.[13] The numbers inside each box indicate the ranking. Abbreviations: SSA: Sub-Saharan Africa

eFigure 4: Liver cancer ranking by total incidence and mortality based on global level, SDI quintiles, regions, and all countries, both sexes, 2015

©2017 Global Burden of Disease Liver Cancer Collaboration. JAMA Oncology.

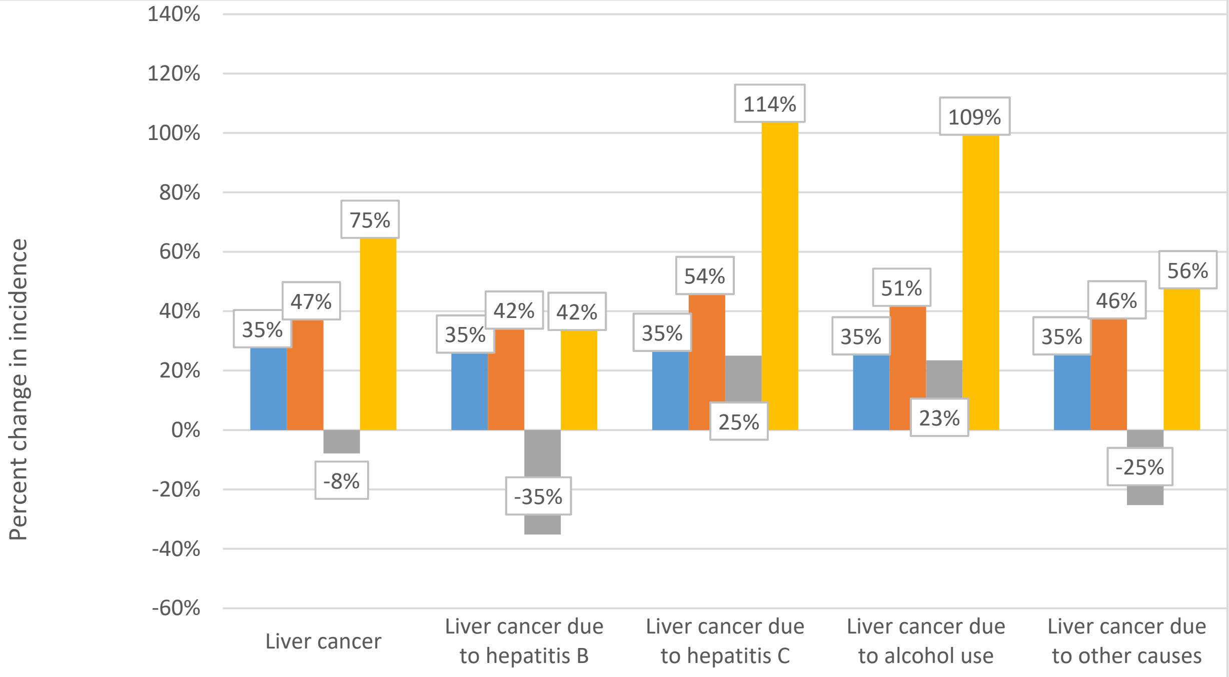

	Liver cancer	Liver cancer due to hepatitis B	Liver cancer due to hepatitis C	Liver cancer due to alcohol use	Liver cancer due to other causes
Population growth	35%	35%	35%	35%	35%
Change in age structure	47%	42%	54%	51%	46%
Change in age-specific incidence rates	-8%	-35%	25%	23%	-25%
Overall change	75%	42%	114%	109%	56%

eFigure 5: Decomposition of changes in liver cancer incident cases 1990 to 2015 by cause group, both sexes. Differences in incident cases between 1990 and 2015 can be explained by three factors: population growth, population age and sex structure, and age- and sex-specific rates. To determine how population growth contributed, two scenarios were calculated: in scenario (1) the 1990 rate, sex, and age structure was applied to the 2015 population. The difference between the 1990 incident cases and scenario (1) is only due to population growth. To estimate the effect of aging, the 1990 age-sex-specific rates were applied to the 2015 age-sex-specific population numbers for scenario (2). The difference between scenario (2) and scenario (1) is due to aging of the population. The contribution of a change in age-specific rates can be determined by taking the difference between 2015 numbers and scenario (2). The effects were calculated as the percent change in the number of cases for each factor compared to 1990.

©2017 Global Burden of Disease Liver Cancer Collaboration. JAMA Oncology.

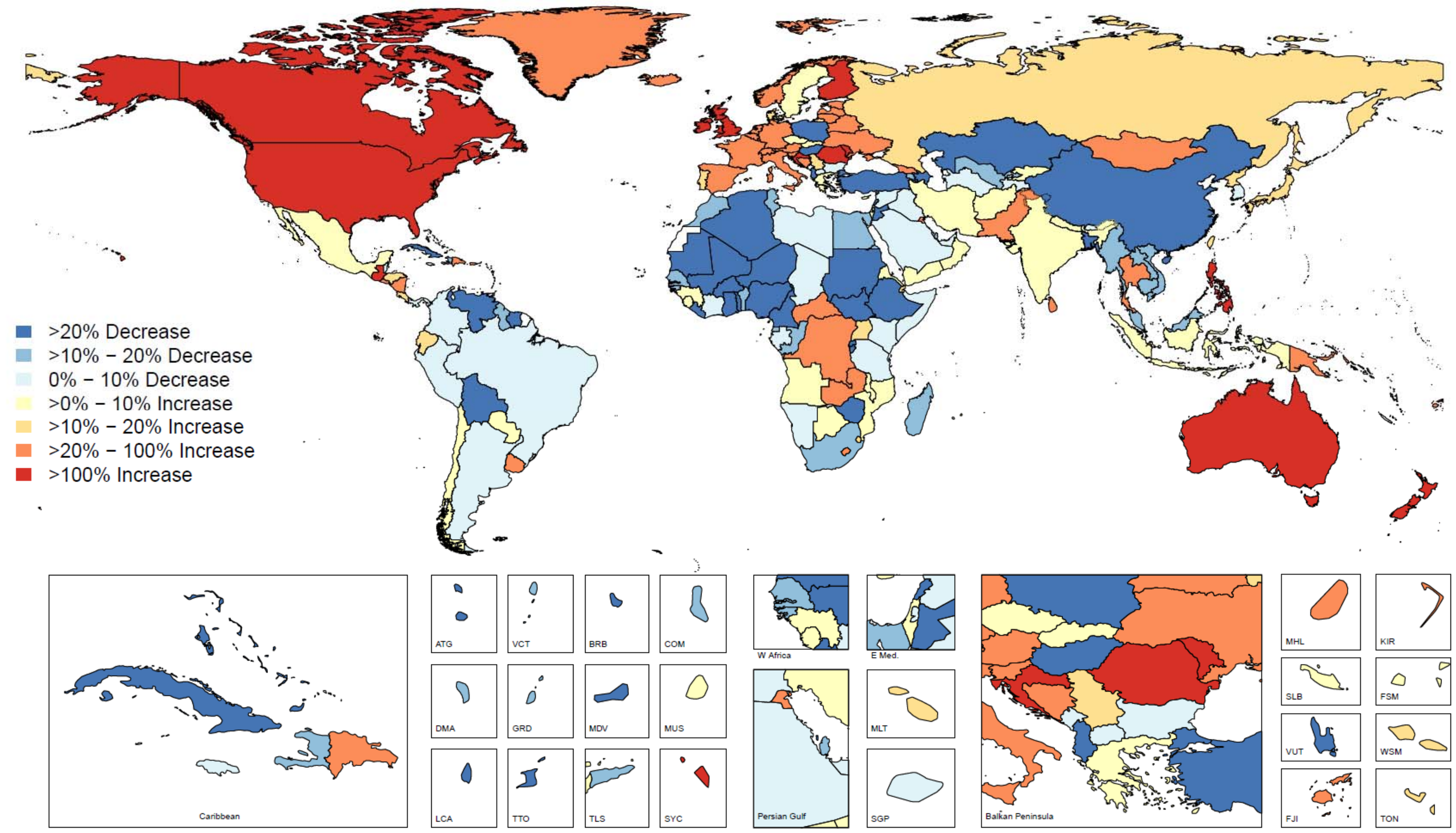

eFigure 6: Relative changes in age-standardized liver cancer incidence between 1990 and 2015 for both sexes in 195 countries and territories

©2017 Global Burden of Disease Liver Cancer Collaboration. JAMA Oncology.
